# Artificial Intelligence for the Diagnosis and Management of Cancers: Potentials and Challenges

**DOI:** 10.1002/mco2.70460

**Published:** 2025-11-05

**Authors:** Man Wang, Wenguang Chang, Yuan Zhang

**Affiliations:** ^1^ Institute For Translational Medicine The Affiliated Hospital of Qingdao University College of Medicine Qingdao University Qingdao China

**Keywords:** artificial intelligence, cancer, cancer diagnosis, prognosis prediction, personalized cancer therapy

## Abstract

Cancer continues to be one of the primary causes of death worldwide. Although there has been substantial progress in clinical cancer care, the outcomes for cancer patients still remain poor. The rapid advancements of artificial intelligence (AI) will revolutionize cancer management by addressing current obstacles in oncology research and practice, ultimately enhancing healthcare accuracy and patient outcomes. Increasing evidence demonstrates that AI‐based models can improve the accuracy and efficiency of cancer diagnosis and treatment by leveraging multilayer data. Cancer patients could greatly benefit from AI's promising prospects, yet few AI models have been authorized for clinical use. A comprehensive understanding of AI's basic principles, applications, and potential impacts is essential to foster its clinical translation. In this review, we provide an overview of fundamental AI techniques, encompassing machine learning and deep learning. Moreover, we summarize recent studies on AI's transformative role in cancer diagnosis, classification, and personalized treatment planning. Furthermore, we discuss the current challenges that hinder the widespread use of AI, propose potential solutions, and outline future directions. Overall, through systematic analysis of existing preclinical and clinical evidence, this review highlights the substantial potential of AI technology and provides valuable guidance for future research in AI‐driven oncology.

## Graphical abstract

Artificial intelligence (AI) is a rising star in the field of medicine, avoiding misdiagnosis by human negligence, reducing the workload of doctors, and aiding clinicians in decision‐making. AI can increase the accuracy and efficiency of cancer identification, classification, treatment assessment, and prognostic prediction, all of which are essential to improve patient outcomes. AI holds immense potential to assist medical experts in developing patient‐tailored treatments for cancer patients. Increasing employment of AI technologies in medical oncology will renew the hopes of individualized patient cancer care achieving clinical reality for various cancers.

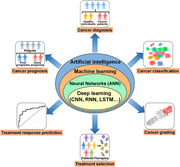

## 1 Introduction

Cancer continues to be a leading cause of death worldwide, responsible for millions of deaths each year [1]. Despite significant advancements in treatment and understanding over the past few decades, several unmet needs persist, which impact patient outcomes, quality of life, and the efficiency of healthcare systems [2]. Specifically, cancer early detection remains crucial but elusive for many cancer types [3]. The lack of accurate biomarkers for early detection and inadequate access to cancer screening programs contribute to delayed diagnosis. Late‐stage diagnosis often leads to higher treatment costs and poor prognosis. Chemotherapy, radiation, and immunotherapy are currently the standard curative treatments for cancer [4]. However, many patients experience severe side effects, such as cardiotoxicity and neuropathy, from treatments, and these issues are often not adequately managed in follow‐up care [5]. Moreover, the high cost of cancer care poses a significant burden on both patients and healthcare systems [6]. Advanced treatments, such as immunotherapy and targeted therapy, are usually expensive, leading to issues of accessibility and equity in treatment options [7]. Furthermore, formulating individualized treatment plans remains challenging due to the heterogeneity of tumors, which causes significant variations in patients’ responses to treatment, even for the same type of cancer [8]. Existing treatment measures usually fail to meet the needs of all patients. For instance, while immunotherapy has revolutionized treatment for some cancers, its effectiveness varies widely across different cancer types and individual patients [9]. In addition, there is an urgent need to improve tumor monitoring and follow‐up methods. Conventional imaging examinations and biomarker tests have limitations in terms of sensitivity and specificity and cannot promptly reflect the dynamic alterations of tumors [10, 11]. Altogether, persistent unmet needs in cancer practice underscore the imperative for ongoing research and comprehensive strategies to address the multifaceted challenges associated with this disease.

**FIGURE 1:**
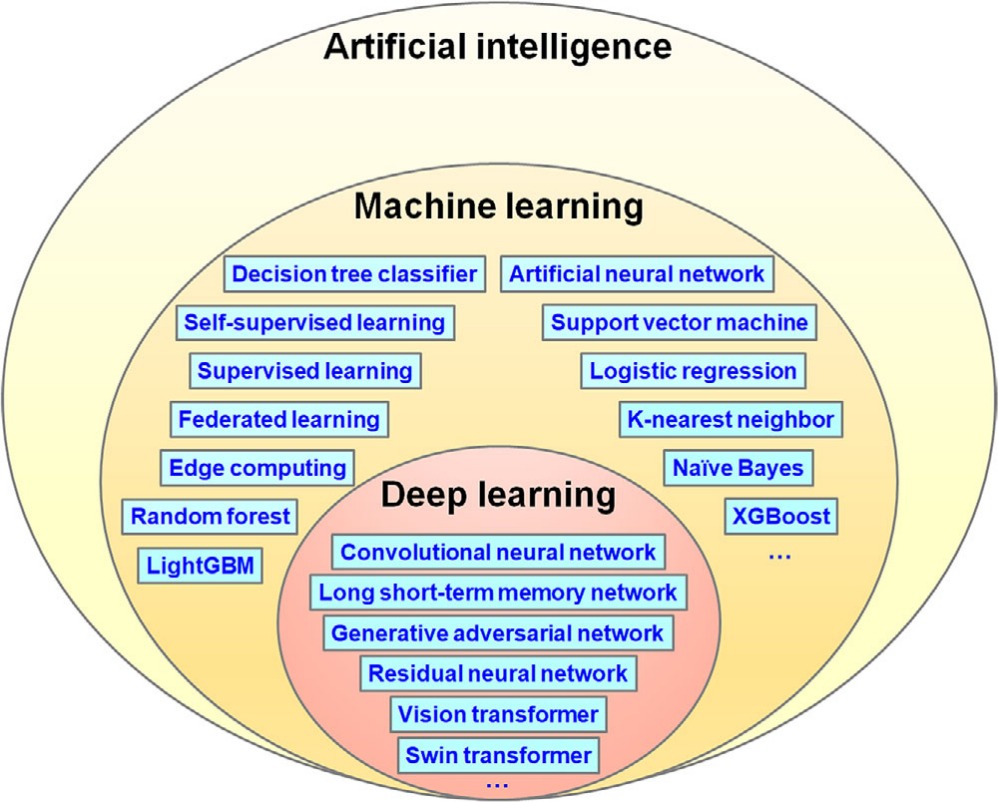
Venn diagram showing the relationship among artificial intelligence, machine learning, deep learning, and commonly used algorithms.

Integration of artificial intelligence (AI) technology into cancer medicine is emerging as a powerful approach to overcome many obstacles where medical experts fail to control and treat cancers in the clinical setting [12]. AI, also known as machine intelligence, represents a new technical subject that explores and develops theories, approaches, techniques, and application programs to imitate and broaden human intelligence in machines [13]. The technical architecture of AI primarily consists of four key modules: human–computer interaction, image recognition, machine learning, and natural language processing (NLP) [14, 15]. Machine learning, a specialized branch of the AI field, leverages statistical techniques to build intelligent systems (Figure 1) [16]. Representative machine learning algorithms include artificial neural network (ANN), deep learning (DL), decision tree, and enhancement algorithms [17]. In recent years, AI has received considerable attention owing to its exemplary advances in computer vision, such as object recognition and image classification [18]. AI is rapidly progressing in many aspects of cancer research and practice, offering transformative opportunities to improve cancer diagnosis, treatment, and prognosis [19]. In diagnostic imaging, AI algorithms can detect tumors with high precision, assisting radiologists and pathologists in interpreting radiological images and tissue samples more accurately and swiftly [20]. Beyond diagnostics, AI leverages patient data, including genetic profiles, medical history, and tumor characteristics, to recommend tailored treatment plans, hence personalizing cancer care [21]. AI systems facilitate matching patients to appropriate clinical trials based on their unique molecular and clinical profiles, accelerating access to cutting‐edge treatments [22]. AI also plays a crucial role in optimizing treatment plans to minimize side effects while maximizing efficacy against cancer, improving the quality of life for patients [23]. Furthermore, AI‐powered tools predict patient responses to various treatments, enabling oncologists to make real‐time adjustments for better outcomes [24]. AI models monitor patients remotely posttreatment, detecting early signs of complications or tumor recurrence, which is essential for timely interventions and improved prognoses [25].

Although AI holds immense potential in cancer diagnosis and management, several challenges and misconceptions need to be addressed to fully realize its benefits. First, there is a common misconception that AI is a “magic bullet” capable of solving all challenges in cancer care. It is crucial to understand that AI programs are designed to augment human expertise, not replace it [26]. Medical professionals remain indispensable in making informed decisions and providing personalized care. Second, many AI models are still in the research phase and have not yet been validated in clinical settings [27]. The transition from theoretical efficacy to practical application warrants rigorous testing and verification to ensure their reliability when used on patients. The accuracy of AI systems is highly dependent on the quality of patient data [28]. High‐quality, annotated datasets are essential for training these models, but accessing such data remains a significant bottleneck in developing efficient AI tools. Investment in better data collection and annotation processes is therefore critical. Third, the regulatory framework for AI in healthcare is still evolving [29]. Ensuring the safety, efficacy, and transparency of AI systems is crucial for building trust among patients and practitioners. Ethical concerns, particularly regarding patient privacy and consent, must be strictly addressed when utilizing AI to analyze sensitive health information [30]. Fourth, infrastructure and expertise pose another challenge. Many healthcare systems lack the necessary infrastructure and skilled personnel to effectively integrate and utilize AI tools [31]. This can lead to disparities in care, with only well‐funded institutions having access to these advancements. In addition, clinicians may be hesitant to adopt AI systems due to a lack of understanding or trust in how these tools function [32]. Education and transparency regarding the decision‐making processes of AI could help alleviate these concerns and foster greater acceptance among medical professionals [33]. Fifth, developing and deploying AI solutions can be quite expensive, which acts as a significant barrier [34]. Expensive development and implementation costs might limit access to AI technologies, potentially widening healthcare disparities between institutions with different financial resources. Last, there is a risk that overreliance on AI could cause medical professionals to overlook their own clinical expertise or patient‐specific factors [35]. AI should be viewed as a decision‐support tool, complementing human judgment rather than replacing it.

Over the past decade, with the ongoing investigation and development of AI, numerous reviews have been conducted [36, 37, 38]. However, there is a lack of systematic analyses on the applications of AI across different types of cancer. This comprehensive review delves into the latest advancements in biomedical application of AI in oncology across various cancer types, including breast, lung, gastric, liver, and prostate cancers. Multiomics, also referred to as integrated omics, involves combining two or more types of omics datasets to enhance data analysis, visualization, and interpretation [39]. By examining cutting‐edge developments in diagnostic imaging, multiomics, and personalized treatment planning, this review aims to illustrate how AI is transforming cancer care. Moreover, we delve into the current challenges and obstacles in implementing AI technology in clinical cancer settings. We also explore possible solutions and future directions. As AI becomes more integrated into medical settings and new AI‐driven therapeutic options emerge, there is growing optimism that personalized cancer treatment for a broad spectrum of cancers will become a reality, accelerating progress in this field.

## 2 AI Foundations for Oncology: Beyond Algorithms

AI technologies are revolutionizing modern medicine by driving breakthroughs in diagnostic accuracy, precision oncology, and predictive analytics [40]. By leveraging large‐scale data analysis, AI enhances disease risk prediction and improves patient outcomes. Predictive analytics empowers effective patient care and optimizes healthcare resource allocation by providing insights into potential future scenarios [41]. Precision medicine tailors healthcare based on an individual's unique biological characteristics, instead of relying on generalized population data [42]. This can be achieved by collecting patient‐specific physiological data, electronic medical records, and other health information, then applying advanced models to customize treatment strategies [43]. A thorough understanding of AI technology forms the essential foundation for healthcare practitioners to effectively integrate and utilize AI tools in clinical practice, significantly elevating their professional capabilities [44]. AI fundamentals cover essential concepts in machine learning, neural networks, and DL (Figure 1).

### 2.1 Machine Learning Paradigms: Supervised versus Self‐Supervised versus Reinforcement Learning

**FIGURE 2:**
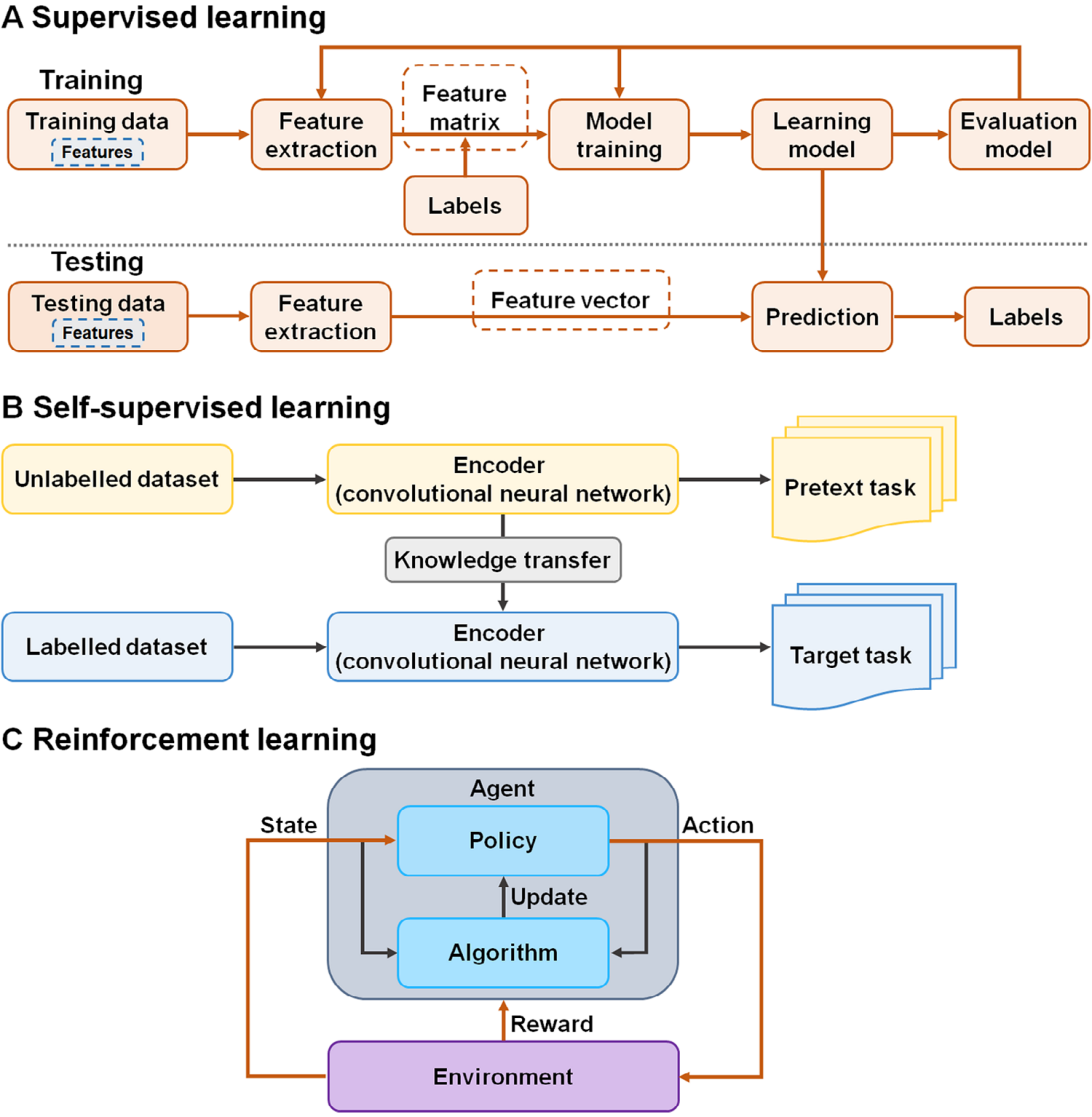
Schematic representations of supervised, self‐supervised, and reinforcement learning models. (A) Supervised learning structure. In this paradigm, the model is trained using labeled datasets, where the model learns to recognize various types of data. After completing the training process, the model is evaluated using a test database. Based on this evaluation, the model then predicts the output for new, unlabeled data. (B) Self‐supervised learning structure. In this paradigm, a deep learning algorithm (e.g., CNN) is trained on unlabeled data using a proxy task. The obtained knowledge is transferred into a downstream task (target task). (C) Reinforcement learning structure. In reinforcement learning, data are not used as input but are accumulated through interactions with the environment. Instead of specifying optimal actions in advance, this system relies on a reward–penalty mechanism. The agent receives feedback immediately after each action, which allows it to learn iteratively. The agent aims to take the optimal action in a given state to maximize the total reward.

Machine learning is a branch of AI that addresses problems by employing self‐improving models that learn from data rather than adhering to predefined rules [45]. Some key concepts form the foundation of machine learning. A dataset is a collection of data arranged in a clear, logical format. Measurable characteristics of the data are called features. A model, the output of a machine learning algorithm trained on datasets, can make predictions or decisions. Labels represent target variables or outputs that the model aims to predict. The loss function is responsible for calculating the disparity between the model's predictions and actual labels. Hyperparameters refer to predefined external configurations that control model training, such as learning rate, neural network hidden layers, and decision tree depth. Training data serve to teach the AI model, and test data are utilized to estimate model performance following training. Overfitting occurs when a model performs well on training data but poorly on new data. To solve this issue, a validation dataset is used to fine‐tune hyperparameters and prevent overfitting. Machine learning is becoming increasingly valuable in clinical oncology, where it is applied to various crucial tasks [46]. These include disease diagnosis through the analysis of medical imaging data, prediction of patient outcomes based on multiple variables, and customization of treatment plans tailored to individual patients. Within machine learning, models are trained to make accurate predictions or classifications based on large datasets. This field encompasses several categories of conceptual models, primarily categorized into three major types of algorithms: supervised learning, unsupervised learning, and reinforcement learning [47]. Supervised learning is a subset of machine learning that relies on labeled input and output data for its learning process [48] (Figure 2A). In supervised learning, the model learns to associate variables from input data, known as features, with the desired output [47]. This process involves creating a labeled dataset, referred to as training data, which often requires manual annotation. The annotated data serve as the ground truth upon which the algorithms’ predictive outcomes are based. There is a wide range of learning models, including basic logistic regression, random forest, neural network, and support vector machine (SVM) [49]. After being trained on associations between input features and an outcome of interest, trained models can precisely predict outcomes in new, unencountered cases. For instance, in the context of cancer imaging analysis, a substantial volume of labeled medical imaging data can be used to train DL models that are capable of automatically identifying the presence and type of cancers [50]. One of the key advantages of this approach is its high accuracy and reproducibility, which can markedly enhance the efficiency of cancer early diagnosis. However, a notable limitation of supervised learning is its heavy dependence on labeled data, and acquiring high‐quality labeled data is often a time‐consuming and costly process [51].

Self‐supervised learning offers a new approach to address such challenge. This paradigm is a type of unsupervised learning that enhances the performance of multiple downstream computer vision tasks, including object detection, image understanding, and image segmentation [51]. By leveraging unstructured and unlabeled data, it enables the development of versatile AI systems at a low cost. Self‐supervised learning acts as a bridge between supervised and unsupervised learning approaches. The self‐supervised learning model trains on one portion of the input data to predict another part, a process known as predictive or pretext learning [52]. Self‐supervised learning exhibits the ability to automatically generate labels for unlabeled data, effectively transforming an unsupervised model into a supervised one. Self‐supervised learning does not rely on manual labeling and are divided into two types: pretext task and downstream target task [53] (Figure 2B). A pretext task utilizes supervised learning to learn representations, with labels generated directly from the data itself [54]. Once this learning is complete, the model utilizes the learned representations to the downstream task. Self‐supervised learning presents an attractive solution to the challenges posed by the difficulties in curating large‐scale annotations. Unlike supervised learning, self‐supervised learning enables versatile models that can be fine‐tuned for multiple downstream tasks without requiring extensive labeled datasets [55]. This strategy not only reduces the reliance on labeled data but also improves the generalization ability of the model. Self‐supervised learning models have shown enhanced performance in diverse cancer classification and survival prognosis tasks when compared with conventional supervised learning models [56, 57].

As a machine learning paradigm, reinforcement learning trains an agent to make optimal decisions in uncertain environment to maximize cumulative rewards [58]. This paradigm involves agent learning via direct interaction with its environment, without relying on labeled examples or fully predefined models (Figure 2C). Reinforcement learning stems from two foundational fields: psychology, which introduces trial‐and‐error learning principles, and optimal control, which contributes value functions and dynamic programming techniques [59]. Through trial‐and‐error, computational algorithms determine the best actions within a certain condition and optimize their performance. The computer receives positive or negative feedback based on its actions and learns how to complete a task. Reinforcement learning consists of several key elements, including an agent, an environment, an action, a reward, a state, and a value [60]. The agent refers to an algorithm that is trained to perform task within an environment. The agent explores and interacts with the environment. It comprises three main components: a policy that refers to the agent's way of behaving in the environment and which strategy is adopted to achieve the goal, a value function that defines the value of each state the agent reaches to evaluate state's effectiveness, and a model that refers to a prediction algorithm that forecasts the next state based on the next immediate reward [60]. The environment is real‐world problems or simulated environment in which an agent operates or takes actions [61]. An action is the move taken by an agent within an environment that causes an alteration in the status. A reward is a feedback given to an agent in response to its action, which may be either positive or negative. Reinforcement learning uses a formal framework to model the interaction between an agent and its environment in terms of states, actions, and rewards [62]. This framework captures the key characteristics of AI‐related issues, incorporating causality, uncertainty, nondeterminism, and explicitly defined goals. Without training datasets, the problem is solved by the agent's actions with input from the environment. Reinforcement learning performs well in unpredictable environments, making it ideal for real‐world applications with uncertain or dynamic conditions. Reinforcement learning has been widely applied in robotics and rare disease diagnosis [63].

Overall, these machine learning algorithms possess their own unique advantages. Combing these approaches may yield a more effective solution for early cancer diagnosis and personalized treatment. Substantial efforts are required to explore the synergistic effects of these learning paradigms.

### 2.2 DL Architectures: Applicability of CNNs, Transformers, and GANs to Oncology Data

DL has revolutionized the field of AI, achieving outstanding performance across various applications [64]. It has proven to be highly effective in handling large volumes of data and performing intricate computations. As a subset of machine learning, DL employs architectures consisting of multiple layers of nodes or neurons, with each layer designed to model increasingly complex patterns in the data [65]. In recent years, DL has demonstrated substantial potential in cancer diagnosis and management, particularly through the application of architectures such as convolutional neural network (CNN), transformer, and generative adversarial network (GAN). This section delves into the foundational architectures that have paved the way for modern DL and explores recent breakthroughs that are broadening the capabilities of neural networks.

#### 2.2.1 Convolutional Neural Network

**FIGURE 3:**
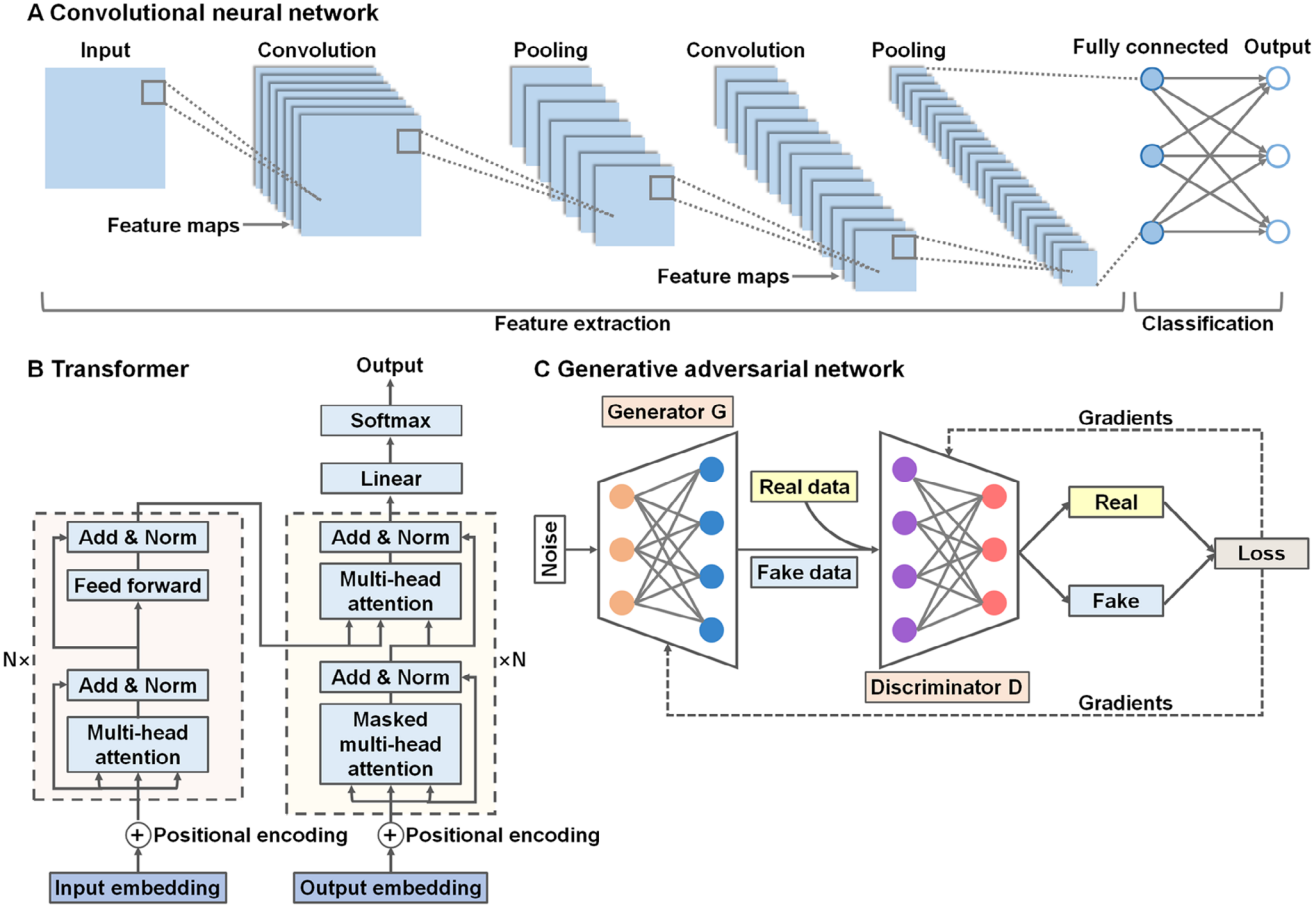
Structure diagrams of different deep learning models. (A) Typical structure of a convolutional neural network. Convolutional neural network is primarily composed of convolution layers, pooling layers, and a fully connected layer. Convolution layers serve as the fundamental building block of convolutional neural network. They bear the main computational burden of the network. Pooling layers aim to decrease the dimensionality of the feature map. This is commonly achieved through average pooling or max pooling operations. The fully connected layer plays a crucial role in mapping the representation between the input and the output. (B) The architecture of a transformer model. The main component of a transformer model is the attention mechanism. The core function of the attention mechanism is to model interactions between different elements in a sequence, capturing dependencies among them regardless of their positions in the sequence. Besides the self‐attention mechanism, another crucial component of the transformer architecture is the integration of positional encoding. Positional encoding incorporates information about the position of elements within the sequence. Adding positional encodings to the model allows it to utilize the sequence's order, which is significant for understanding structured data. (C) The basic architecture of a generative adversarial network. Generative adversarial network consists of two neural networks: a generator and a discriminator. The generator produces synthetic data (e.g., images, videos, or text) that closely resemble real‐world data. The discriminator is responsible for assessing the data and differentiating between real and fake data.

CNN, a subtype of ANN, stands as a cornerstone architecture, transforming the manner in which neural networks extract knowledge from data [66]. CNN is characterized by architectures that incorporate multiple hidden layers to progressively extract higher‐level features from raw input data. It plays a pivotal role in advancing computer vision tasks, including image classification, object detection, and image segmentation. By employing convolutional layers, CNN can automatically learn spatial hierarchies of features from input data, enabling the network to capture patterns such as edges, textures, and complex shapes [67]. A standard CNN architecture consists of several components: convolutional layers, pooling layers, and a fully connected layer, which collectively lead to an ultimate prediction [65] (Figure 3A). In a CNN, convolutional layers utilize a set of learnable filters or kernels to the input data, generating feature maps. Subsequently, pooling layers, commonly max‐pooling, downsample these feature maps to reduce their dimensionality. This reduction not only helps prevent overfitting but also decreases computational complexity. Following several convolutional and pooling layers, the network incorporates a fully connected layer, similar to those in standard neural networks. These layers combine extracted features to make final predictions for classification or regression tasks [66]. Architectures such as AlexNet, LeNet, ResNet, and VGGNet have progressively enhanced CNN's ability to handle increasingly complex visual tasks. Notably, innovations like skip connections in ResNet facilitate efficient gradient flow through deeper networks, addressing issues such as vanishing gradients and enabling the training of exceptionally deep architectures [68]. Nevertheless, CNN is constrained by its reliance on large quantities of labeled data and high computational demands, which have spurred the development of more efficient and flexible architectures [69]. CNN has emerged as the primary tool for medical image analysis due to their excellent performance in image processing. It can effectively identify morphological and structural changes in tumors, thus enhancing the accuracy of cancer diagnosis [70].

#### 2.2.2 Transformer

The transformer architecture excels at processing sequential data and is particularly well suited for analyzing genomic data and clinical records [71]. Unlike conventional methods that depend on recurrent layers, the transformer model is built on an encoder–decoder architecture, with both components consisting of multiple layers that employ self‐attention mechanisms and feed‐forward neural networks [72] (Figure 3B). This design enables parallel processing of input data, making the model highly efficient and effective for tasks involving sequential data. This advancement not only boosts training efficiency but also improves model performance on large datasets. Its self‐attention mechanism allows the model to focus on important parts of the input data, thereby capturing complex biological relationships [72]. This capability makes transformers highly valuable in the development of personalized treatment plans and cancer prognosis assessment. Furthermore, transformers are revolutionizing various fields, particularly in generative tasks and reinforcement learning, due to their ability to model intricate patterns and relationships within data [73]. Transformers have demonstrated remarkable effectiveness and scalability, paving the way for next‐generation models that could provide deeper insights across various domains [74]. This underscores their substantial contribution to advancing both technology and DL methodologies.

#### 2.2.3 Generative Adversarial Network

GAN represents a pioneering category of DL models comprising two interlinked neural networks: the generator and the discriminator [75] (Figure 3C). These networks engage in an ongoing game‐theoretic competition throughout their training phase. The generator's primary goal is to create data that closely resemble real‐world data, effectively fooling the discriminator [76]. Meanwhile, the discriminator strives to differentiate between real data sourced from the dataset and fake data generated by the generator. These training processes occurs concurrently, with both networks competing to outperform the other. It is crucial to note that the generator does not have direct access to real images. Instead, it learns solely through its interaction with the discriminator. This discriminator has access to both synthetic samples produced by the generator and actual images from a dataset [77]. The discriminator is trained using ground truth labels indicating whether an image is real or synthetic. The error signal for the discriminator comes from these labels, allowing it to distinguish between real and fake images effectively. Importantly, this same error signal can be leveraged to train the generator. By receiving feedback from the discriminator about the quality of its generated samples, the generator can iteratively improve, aiming to produce more realistic forgeries. Altogether, the interplay between the generator and the discriminator, with the discriminator providing critical feedback, drives the generator to enhance the quality of its synthetic images [78]. GAN offers a way to perform advanced domain‐specific data augmentation and provide solutions for tasks that necessitate a generative approach, such as image‐to‐image translation. In cancer research, GAN can be employed to generate imaging data of rare cancer types, helping researchers conduct more comprehensive model training [79]. In addition, GAN can be harnessed to create personalized treatment plans for patients, advancing the field of precision medicine.

While DL architectures have demonstrated substantial benefits in cancer diagnosis and management, they still encounter challenges such as data privacy, model interpretability, and transitioning to clinical applications [80]. Consequently, additional study is warranted to address these issues while enhancing model performance, thereby facilitating the broad adoption of AI in oncology research.

### 2.3 Federated Learning and Edge Computing: Addressing Privacy and Real‐Time Processing Constraints

**FIGURE 4:**
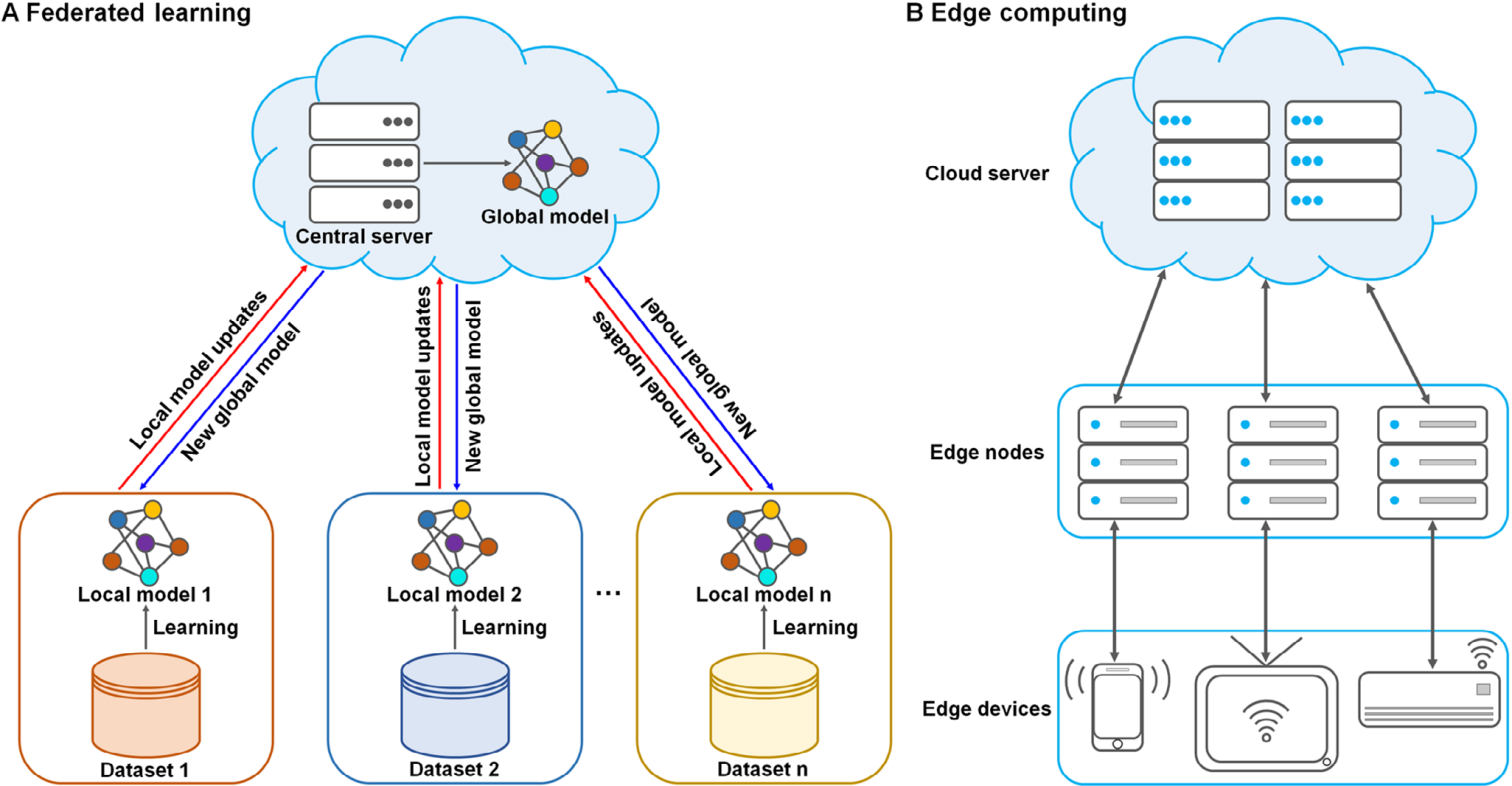
Basic architectures of federated learning and edge computing. (A) A schematic architecture of a federated learning model. Federated learning is a machine learning technique that allows for the training of models on decentralized data, which is distributed across multiple devices. Federated learning functions by distributing computations to local devices or nodes. Each participating device trains the model locally using its private datasets and shares only the model updates with a central server. The central server then aggregates these updates to create a global model. (B) Edge computing architecture. A typical edge computing architecture is composed of three main layers: the cloud layer for secure storage and deep analysis, the edge layer for real‐time data processing, and the device layer for data collection.

Conventional AI algorithms frequently rely on a single, centralized dataset for training within a unified system. Although this approach seems straightforward for developing AI tools, it comes with substantial risks and vulnerabilities. Specifically, the necessity to centralize all data in one location and the potential for data exposure during transmission raise significant concerns. Federated learning steps in to address the growing privacy and security concerns associated with traditional AI models [81]. Federated learning enables multiple medical institutions to collaboratively train machine learning models without sharing patient data [82] (Figure 4A). This approach allows each institution to leverage the diversity of their local data to enhance the model's generalization ability while safeguarding patient privacy. By keeping data localized and exchanging only model updates through the communication network, federated learning ensures that sensitive information remains protected while still allowing for collective learning [81]. Federated learning can be categorized based on data distribution, specifically into vertical and horizontal federated learning [83]. Vertical federated learning is applicable when data are partitioned vertically according to the feature dimension [84]. In this scenario, data feature are distributed among multiple parties, and the goal is to collaboratively train a model using the combined dataset. In horizontal federated learning, similarities exist in the data features distributed across multiple nodes [82]. However, these data differ significantly in their sample spaces. Current federated learning algorithms are mainly designed for many applications involving internet of things (IoT) devices or smart devices [85].

As IoT continues to evolve and its applications become increasingly ubiquitous, cloud computing is encountering numerous limitations and challenges. For instance, processing and storing data from global terminal devices in centralized clouds lead to several issues: low throughput, high latency, bandwidth constraints, data privacy concerns, vulnerabilities associated with centralization, and increased costs (e.g., transmission, energy, storage, and computation expenses) [86]. In IoT applications, especially those involving the internet of vehicles, there is a critical need for high‐speed data processing, analysis, and rapid result delivery [87]. Centralized cloud computing often cannot satisfy these requirements efficiently. To tackle these challenges posed by traditional cloud computing, a novel computing paradigm known as edge computing has emerged and garnered significant attention [88]. Edge computing aims to alleviate the workload on cloud servers by processing and preprocessing device data at the network edge [89]. This makes it well suited for big data analytics and scenarios requiring real‐time responses or applications with time‐critical constraints [90].

The general architecture of edge computing is composed of three layers: the device layer, the edge layer, and the cloud layer [91] (Figure 4B). The device layer serves two primary purposes [92]. First, it perceives the world by observing, obtaining, and digitizing information from the physical environment through various sensors. Second, it receives information or data from the edge or cloud and performs corresponding tasks based on that data. Devices in this layer typically have minimal computing and storage capabilities [93]. The edge layer, situated between the cloud and the end layers, contains computing, storage, and network resources, allowing it to undertake tasks that were traditionally handled by the cloud layer [94]. Its proximity to end devices reduces latency, making it advantageous for real‐time applications. Generally, the edge layer consists of gateways, control units, storage units, and computing units. The cloud layer refers to the cloud servers widely used in practice [91]. Besides their robust computing and storage capabilities, cloud servers also have the ability to macro‐manage the entire edge computing architecture [95]. Edge computing offers several advantages by offloading resources and tasks from the cloud to the edge. The proximity of the edge layer to end‐users and data sources significantly reduces transmission distances, leading to decreased transmission times and improved response speeds for user requests. Additionally, shorter transmission distances help mitigate costs associated with long‐distance data transfer and address data security concerns [96]. From the cloud's perspective, processing large volumes of raw data at the edge layer allows for the filtering out of useless and erroneous data before uploading only essential information or important data to the cloud layer [97]. This approach notably reduces bandwidth pressure, transmission costs, and the risk of user privacy leakage. In the preprocessing phase of raw data within edge computing, confidential information unrelated to the final task can be obscured or deleted prior to sharing with external devices [98]. This approach mitigates the risk of leaks involving sensitive or confidential data. An indirect advantage of the edge computing paradigm is its facilitation of end device abstraction, particularly when dealing with heterogeneous devices that collect complementary information [99]. In such scenarios, an adaptation layer is necessary to standardize diverse data into a unified structure. Edge computing integrates this abstraction layer within each end device, ensuring that only relevant, transformed data are shared for the fusion process. This approach significantly reduces the computational burden on cloud servers that would otherwise be required to integrate raw, heterogeneous data.

### 2.4 Data Cornerstones: Multimodal Data Fusion, Annotation Standards, and Data Governance

Multimodal data fusion has become a transformative approach in clinical oncology, systematically combining multiomics data, medical imaging, laboratory test results, electronic health records (EHRs), and outputs derived from wearable devices [100]. Each data type provides distinct clinical value, yet they may only present a partial perspective. Integrating these data sources enable a more systematic understanding of patient health status. Multimodal models that combine diverse modalities (clinical data and genomic analysis) can improve the timeliness and precision of cancer detection and diagnosis [101]. The synergistic integration of digitalized pathological images with multiomics data facilitate accurate tumor classification [102]. Moreover, multimodal features extracted from histopathological data, spatial, and single‐cell transcriptomics can reveal the composition, spatial structure, and heterogeneity of tumor microenvironment [103]. Furthermore, integrated genomic analysis approaches can identify dysregulated biological functions and molecular pathways, offering novel opportunities for personalized therapy and patient monitoring [104]. The combination of biological information‐driven multimodal imaging techniques allows for precise characterization of tumor heterogeneity and facilitate the selection of optimal treatment [105]. Integrating the features extracted from routine diagnostic approaches, pathology, clinical information, and genomics can enhance the prediction of immunotherapy response [106]. The multimodal approaches also achieve accurate prognostic predictions across diverse cancer types [107]. Although the significant potential of multimodal systems in healthcare, several critical challenges must be overcome to realize its full benefits. Data standardization and privacy protection remain significant issues requiring attention. There is a critical need to improve model interpretability to generate clinically actionable insights. To gain physician trust, it is essential to enhance the transparency of models’ decision‐making processes. Overall, the development of more advanced AI algorithms and data fusion technologies will significantly improve the analysis and interpretation of complex multimodal datasets, enabling more tailored solutions for clinical cancer care [108].

## 3 AI‐Powered Cancer Diagnosis and Molecular Profiling

The application of AI technology in cancer management has shown great promise, particularly in efficient diagnosis. Current diagnosis methods for cancer face several challenges [109]. Early cancer detection is often difficult due to the tumors’ ability to conceal themselves, limitations in imaging technologies, and the difficulty in visualizing small or deeply situated tumors. Accurate differentiation among different tumor types remains challenging in clinical practice [110]. Some diagnostic procedures are invasive and come with associated risks. These approaches are also time consuming, have limited accessibility, and suffer from interpretation variability among experts. Clinicians mainly rely on their expertise and knowledge when evaluating a patient's signs and symptoms. However, given vast amounts of clinical data, it can be difficult for them to make quick and accurate diagnoses. Furthermore, there are challenges related to tumor heterogeneity, individual differences, atypical test results, and false negatives [111]. Heavy clinical workloads increase the risk of missed or incorrect diagnoses among clinicians. There is a clear need for advanced AI‐based diagnostic methods to address these issues. AI possesses remarkable capabilities in efficiently processing large volumes of clinical data [112]. The diagnostic efficacy of AI approaches has been explored in imaging modalities and histopathologic examination [113]. AI is anticipated to advance tumor diagnostics by enhancing accuracy, expediting analytical process, enabling early detection, and improving differentiation between tumor types.

### 3.1 Imaging Diagnostics Revolution

Oncological imaging methods provide a unique and unaltered view of the entire tumor. AI models hold the promise of significantly revolutionizing oncological imaging by leveraging machine learning and DL techniques [114]. Computed tomography (CT) scans are widely used for detecting the presence, size, and location of tumors, as well as whether they have spread to surrounding tissues or other organs. Early cancer diagnosis via nodule detection on CT scans remains crucial for improving patient survival rates. AI algorithms have shown significant potential in CT screening for breast cancer [115, 116], colorectal cancer (CRC) [117], esophageal cancer [118], lung cancer [119, 120, 121, 122, 123, 124, 125, 126, 127, 128, 129, 130, 131, 132, 133, 134, 135, 136, 137, 138, 139, 140, 141, 142, 143, 144], gastric cancer [145], hepatocellular carcinoma (HCC) [146, 147], ovarian cancer [148], pancreatic ductal adenocarcinoma (PDAC) [149, 150], and pancreatic cancer [151, 152, 153, 154, 155] (Table 1). Magnetic resonance imaging (MRI) is a prevalent tool in cancer diagnosis, utilizing strong magnetic fields and radio waves to generate detailed internal body images without radiation exposure [156]. This makes it a safer option, especially for sensitive populations. MRI excels in imaging soft tissues like the brain, spinal cord, joints, and muscles [157]. It aids in detecting tumor size, location, and shape, which is essential for cancer staging and treatment planning. However, MRI scans generally require longer times and are more expensive than other imaging methods. DL methods could improve the detection of breast cancer [158, 159], glioblastoma multiforme [160], glioma [161], intrahepatic cholangiocarcinoma [162], lung cancer [163], and prostate cancer [164, 165, 166, 167, 168] based on conventional MRI. Positron emission tomography (PET) imaging offers detailed images of metabolic processes within the body [169]. This noninvasive technique facilitates early cancer detection and identifies cancers often missed by other methods. AI models enhanced the efficiency of PET screening for lymphoma [170, 171], lung cancer [172, 173], and transformed follicular lymphoma [174].

**TABLE 1:** Summary of AI‐based tools in different cancer diagnosis modalities.

Potential clinical application	Cancer type	AI algorithm	AI task	Algorithm performance	References
Oncological imaging	Breast cancer	Deep learning	Cancer screening using digital mammography and dedicated cone‐beam breast CT	Interquartile range: 0.187; *p* value: 0.862	[115]
Oncological imaging	Breast cancer	Deep learning	Cancer diagnosis based on the Wisconsin Breast Cancer Dataset (WBC)	Recall rate: 98.7%; *F*1‐score: 99.03%; accuracy rate: 99%	[116]
Oncological imaging	Colorectal cancer	Faster region‐convolutional neural network	Cancer detection using CT colonography images	Sensitivities: 0.815 for lesions ≥ 6 mm, 0.738 for lesions = 6–10 mm, and 0.883 for lesions ≥ 10 mm	[117]
Oncological imaging	Esophageal cancer	Convolutional neural network	Cancer diagnosis using cancerous and noncancerous CT images	Accuracy rate: 84.2%; *F* value: 0.742; sensitivity: 71.7%; specificity: 90.0%	[118]
Oncological imaging	Lung cancer	3D convolutional neural network	Cancer screening using CT images	Reduce the risk of patient health information exposure	[119]
Oncological imaging	Lung cancer	Deep learning	Cancer early detection using 3D CT images	Accuracy: 0.959	[120]
Oncological imaging	Lung cancer	Deep learning	Cancer screening using CT images and clinical data	AUC: 0.8021	[121]
Oncological imaging	Lung cancer	Transfer learning	Cancer detection using chest CT images	Accuracy rate: 91%; precision: 92%; recall: 91%; *F*1‐score: 91.3%	[122]
Oncological imaging	Lung cancer	Deep learning	Cancer screening using chest CT images	Effectively measure bone mineral density with an accuracy level comparable to manual measurements	[123]
Oncological imaging	Lung cancer	Densely connected convolutional neural network	Cancer detection using lung CT images	Accuracy rate: 98.17%; specificity: 97.32%; precision: 97.46%; recall: 97.89%	[124]
Oncological imaging	Lung cancer	Machine learning; deep learning	Cancer early screening using radiology, clinical and genomics data	AUC: 0.587–0.910	[125]
Oncological imaging	Lung cancer	Deep learning	Cancer screening using lung imaging data and clinical metadata	AUC: 0.88	[126]
Oncological imaging	Lung cancer	Multimodal integrated feature neural network	Cancer diagnosis using CT images and morphological characteristics of pulmonary nodules	Internal AUC: 0.890; external AUC: 0.843	[127]
Oncological imaging	Lung cancer	Three‐dimensional convolutional neural networks	Cancer early detection using lung CT images	AUC values: 0.801 for 3D‐CNN, 0.802 for MobileNet v2, 0.755 for EfficientNet‐B0, 0.833 for SEResNet18	[128]
Oncological imaging	Lung cancer	Deep learning	Cancer screening using CT images	AUC: 0.8719	[129]
Oncological imaging	Lung cancer	Deep learning	Cancer early detection using single low‐dose CT images	AUC: 0.98	[130]
Oncological imaging	Lung cancer	Deep learning	Cancer screening using low‐dose CT images	AUC: 0.89 ± 0.02	[131]
Oncological imaging	Lung cancer	Machine learning	Cancer screening using modified CT index and serological indices	AUC values: 0.931 in the validation set, 0.99 in the test set; accuracies: 0.857 in the validation set, 0.955 in the test set	[132]
Oncological imaging	Lung cancer	Deep learning	Cancer early detection using low‐dose CT images	AUC: 0.827 ± 0.028	[133]
Oncological imaging	Lung cancer	Deep neural network	Cancer early detection using lung nodule dataset	Accuracy rate: 98.2%	[134]
Oncological imaging	Lung cancer	Machine learning	Cancer early detection using lung CT images	Accuracy rate: 98.21%; precision: 98.71%; recall: 97.46%	[135]
Oncological imaging	Lung cancer	Convolutional neural network	Cancer early detection using CT images	Accuracy rate: 97.56%; specificity: 98.4%	[136]
Oncological imaging	Lung cancer	Deep learning	Cancer early detection using CT images	Accuracy rate: 93.4%; sensitivity: 90.2%; AUC: 94.1%	[137]
Oncological imaging	Lung cancer	Deep learning	Cancer diagnosis using CT images	AUC: 0.9711	[138]
Oncological imaging	Lung cancer	Deep learning; internet of things; convolutional neural network	Cancer early diagnosis using medical data (CT images and sensor information)	Accuracy rate: 98.85%	[139]
Oncological imaging	Lung cancer	Convolutional neural network	Cancer diagnosis using CT images	Accuracy rate: 93.25%; sensitivity: 89.22%; specificity: 95.82%; precision: 92.46%; *F*1‐score: 0.9114; AUC: 0.9629	[140]
Oncological imaging	Lung cancer	Deep learning	Cancer early detection using chest X‐rays and chest CT images	Accuracy rate: 90%; sensitivity: 57.6%	[141]
Oncological imaging	Lung cancer	Deep learning	Cancer screening using low‐dose CT images	Remove noise from CT images and show good detail retention	[142]
Oncological imaging	Lung cancer	Deep learning	Cancer screening using metadata and CT images	Effectively distinguish cancerous cases from benign cases using a combined slice thickness ≥ 2.5 mm	[143]
Oncological imaging	Lung cancer	Deep learning	Lung cancer detection using CT images and chest radiography	AUC: 0.92; sensitivity: 94%; specificity: 73%	[144]
Oncological imaging	Gastric cancer	Deep learning	Automated cancer screening using CT images	AUC: 0.98; accuracy: 0.93; sensitivity: 0.92; specificity: 0.92; *F*1‐score: 0.93	[145]
Oncological imaging	Hepatocellular carcinoma	Deep learning	Cancer detection using solely noncontrast CT images	AUC values: 0.807 on the internal validation cohort, 0.789 on the external testing cohort	[146]
Oncological imaging	Hepatocellular carcinoma	Deep learning	Cancer detection using CT images	Precision: 0.97216; recall: 0.919; accuracy rate: 95.35%; specificity: 95.83%; sensitivity: 94.74%	[147]
Oncological imaging	Ovarian cancer	Deep learning	Automated cancer diagnosis	Accuracy rate: 98.43%	[148]
Oncological imaging	Pancreatic ductal adenocarcinoma	Reinforcement learning; deep neural network	Early cancer diagnosis	Achieve method accuracies of 2.2, 4.9, and 2.6 mm measured in terms of the mean detection error, Hausdorff distance, and root mean squared error	[149]
Oncological imaging	Pancreatic ductal adenocarcinoma	Generative adversarial network	Cancer diagnosis	AUC: 0.79; precision‐recall AUC: 0.87; accuracy: 0.67	[150]
Oncological imaging	Pancreatic cancer	Deep learning	Large‐scale cancer detection	Accuracy: 0.907 ± 0.01; sensitivity: 0.905 ± 0.01; specificity: 0.908 ± 0.02; AUC: 0.903 ± 0.01	[151]
Oncological imaging	Pancreatic cancer	Deep learning	Early cancer detection	AUC: 0.98; sensitivity: 0.94	[152]
Oncological imaging	Pancreatic cancer	Convolutional neural network	Cancer detection	Sensitivity: 0.97; specificity: 1.00; AUC: 0.99	[153]
Oncological imaging	Pancreatic cancer	Convolutional neural network; vision transformer; K‐nearest neighbor; support vector machine; random forest; XGBoost	Cancer diagnosis	Accuracy rate: 97.33%; *F*1‐score: 96.25%	[154]
Oncological imaging	Pancreatic cancer	Convolutional neural network	Cancer diagnosis	Accuracy rate: 99.64%	[155]
Oncological imaging	Breast cancer	Faster region‐convolutional neural network	Cancer detection using breast MRI	Accuracy rate: 94.46%	[158]
Oncological imaging	Breast cancer	Deep learning	Cancer detection using breast dynamic contrast enhanced‐MRI images	Dice and Jaccard coefficients: 0.9468 and 0.8990	[159]
Oncological imaging	Glioma	Convolutional neural network	Cancer diagnosis using MRI images	Accuracy rate: 87%	[160]
Oncological imaging	Glioma	Convolutional neural network; vision transformer; explainable AI	Cancer detection using MRI images	Accuracy rates: 98.4% and 99.3% on two separate datasets	[161]
Oncological imaging	Intrahepatic cholangiocarcinoma	Deep learning	Cancer diagnosis using CT and MRI images	AUC values: 0.994 in the training cohort, 0.937 in the test cohort	[162]
Oncological imaging	Lung cancer	Deep learning	Cancer screening using pulmonary MRI images	Detection rates: ≥96.8%	[163]
Oncological imaging	Prostate cancer	Deep learning	Cancer detection using dense intraslice information and sparse interslice information of the anisotropic bi‐parametric MRI images	AP score: 0.690; AUC: 0.909	[164]
Oncological imaging	Prostate cancer	Support vector machine; XGBoost; deep learning	Cancer identification using bi‐parametric MRI images and clinical variables	AUC values: 0.986 in the training set, 0.965 in the testing set	[165]
Oncological imaging	Prostate cancer	Deep learning	Cancer detection using noncontrast MRI sequences	Simulated contrast‐enhanced MRI images show high similarities (0.69, 0.71, and 0.82) with excellent reader agreement of PI‐RADS scores	[166]
Oncological imaging	Prostate cancer	Deep learning	Cancer detection using clinical information (e.g., prostate MRI)	Positive predictive value: 58% (94 out of 163); specificity: 44% (48 of 108); sensitivity: 96% (93 out of 97); AUC: 0.79	[167]
Oncological imaging	Prostate cancer	Machine learning (XGBoost, random forest, neural network, logistic regression)	Cancer early detection using MRI images and clinical data	Specificities: 0.640, 0.638, 0.634, 0.620	[168]
Oncological imaging	Lymphoma	Deep learning	Cancer diagnosis using PET images	Improve the quality of lymphoma PET with a twofold acceleration	[170]
Oncological imaging	T‐cell lymphoma	Logistic regression	Cancer screening using ^18^F‐FDG PET/CT images	Accuracy: 0.779; AUC: 0.863	[171]
Oncological imaging	Lung cancer	Fully convolutional network	Cancer diagnosis using PET images	Have good performance and time cost	[172]
Oncological imaging	Lung cancer	Adaptive dilated convolution neural network, hybrid attention‐based deep networks	Cancer detection using PET and CT lung images	Demonstrate enhanced effectiveness over traditional detection approaches	[173]
Oncological imaging	Follicular lymphoma	Deep learning, machine learning	Cancer identification using radiomic features extracted from PET/CT images	AUC: 0.820	[174]
Pathology	Breast cancer	Paige Breast Suite	Cancer detection using histopathological images	Undetermined	[175]
Pathology	Breast cancer	Ibex Galen Breast HER2	Cancer detection using breast biopsies	AUC: 0.997	[176]
Pathology	Breast cancer	Mindpeak Breast	Cancer detection using breast biopsies	AUC values: 0.99 for invasive carcinoma, 0.98 for ductal carcinoma in situ	[177]
Pathology	Breast cancer	PathAI: AIM‐HER2	Cancer detection using histopathological images	Enhance diagnostic precision and streamline workflow efficiency	[178]
Pathology	Breast cancer	Visiopharm	Cancer metastasis diagnosis using histopathological images	Sensitivity: 100%; specificity: 41.5%; positive predictive value: 29.5%	[179]
Pathology	Prostate cancer	Paige prostate	Cancer diagnosis using prostatic biopsies	Maintain high diagnostic accuracy; lead to a significant decrease in immunochemistry studies, second opinion requests, and time for reporting	[180]
Pathology	Gastric cancer	Convolutional neural network algorithms	Cancer diagnosis using gastrointestinal endoscopic images	Improve the accuracy and efficiency of current diagnostic methods	[181]
Pathology	Skin cancer	DermAI	Early cancer detection using dermatoscopic images	Mean accuracy rate: 99.60%	[182]

### 3.2 Digital Pathology Breakthroughs

Pathology is essential for confirming the presence of cancer, identifying its type, and assessing its grade [183]. Digital pathology is a groundbreaking development in the field of pathology, involving the scanning of glass slides to create high‐resolution digital slides for analysis [184]. The integration of AI can assist in the rapid and accurate analysis of pathology slides, helping to identify patterns and anomalies that might be missed by human eyes [185]. In recent years, AI tools designed to aid in tumor detection have emerged and are being integrated into routine clinical practice, particularly in the diagnosis of breast and prostate cancers. For instance, several AI‐based tools have been developed and evaluated for accurate diagnosis in breast cancer pathology, such as Paige Breast Suite, Ibex Galen Breast, Mindpeak Breast, PathAI: AIM‐HER2, and Visiopharm (Table 1). Paige Breast Suite assisted pathologists in identifying breast cancer, thus enhancing diagnostic accuracy and effectiveness [175]. Ibex Galen Breast HER2 [176], Mindpeak Breast [177], and PathAI: AIM‐HER2 [178] improved the identification and categorization of various subtypes of breast cancer by accurately analyzing histopathological images. Visiopharm facilitated the precise quantification of ER, PR, HER2, and Ki‐67 biomarkers for breast cancer diagnosis in a digital pathology workflow [179]. Paige Prostate, a clinically validated AI tool, categorized prostate core biopsy whole‐slide images as either “suspicious” or “not suspicious” for prostate cancer [180]. The implementation of Paige Prostate notably reduced reporting time and resource consumption, including IHC studies and second opinion requests. AI tools are also being developed and applied to improve the diagnosis for other cancer types. AI‐aided endoscopic lesion detection systems were able to detect and characterize lesions in the gastrointestinal tract through real‐time image analysis [181]. This enhancement increased diagnostic accuracy and minimized the risk of overlooking lesions. In addition, DermAI served as a useful AI platform for diagnosing skin cancers based on image input [182]. These AI applications provide a comprehensive tumor landscape, facilitating better‐informed decision‐making.

### 3.3 Integrated Multiomics Diagnostics

Multimodal data fusion is a process of combining various data sources and types, such as medical images, pathological, genomic, transcriptomic, proteomic, and clinical data, to gain a thorough understanding of disease complexity [186, 187]. By leveraging the synergies among varied data types, medical professionals can make more informed decisions, improving diagnostic accuracy and enabling the selection of personalized treatment options for better patient care. DL‐based multimodal networks achieve good performance in the detection of breast cancer [188, 189, 190], glioma [191], lung cancer [192], and oral cancer [193] (Table 2). Machine learning‐driven multimodal feature models improved the detection of HCC [194] and bladder cancer [195]. These studies also demonstrated that multimodal fusion modeling of biomedical data achieve better performance than single‐modality approaches. Accordingly, multimodal fusion methods hold great promise for cancer management by providing comprehensive and accurate insights [196]. To fully harness the potential of multimodal fusion approaches, several key improvements are imperative, including resolution of data heterogeneity issues, refinement of feature fusion strategies, and optimization of network architectures.

**TABLE 2:** Summary of AI‐based multimodal models for cancer diagnosis.

Cancer type	AI algorithm	AI task	Algorithm performance	References
Breast cancer	Transformer	Cancer diagnosis using MRI images, lesion characteristics, and clinical information	AUC: 0.928 ± 0.027	[188]
Breast cancer	Vision transformer	Cancer malignancy classification using imaging histopathological features and clinical parameters	AUC values: 0.942 and 0.945 for two independent testing cohorts; *F*1‐scores: 0.872 and 0.857 for the two testing cohorts	[189]
Breast cancer	Convolutional neural network	Cancer segmentation and classification using multimodal ultrasound images	Dice score: 78.23%; intersection over union: 68.60%; precision: 82.21%; recall: 80.58%; accuracy rate: 98.46%	[190]
Glioma	Convolutional neural network, transformer, LightGBM, random forest	Cancer diagnosis using histopathology images and clinical data	Accuracy: 0.936; AUC: 0967	[191]
Lung cancer	Convolutional neural network, ResNet, transformer	Cancer diagnosis using multimodal spectral data and text features	Accuracy rates: 95.83, 97.92, and 100% for three test sets	[192]
Oral squamous cell carcinoma	Convolutional neural network	Cancer detection using histopathological images and complex cancerous patterns	Accuracy rates: 90.98 and 98.24% for two testing datasets; dice similarity coefficients: 86.14 and 94.09% for two testing datasets; mean intersection over unions: 77.10 and 88.84% for two testing datasets	[193]
Hepatocellular carcinoma	XGBoost	Cancer detection using clinical, radiological, and peripheral immunological features	AUC values: 0.985 and 0.915 for the internal and external validation cohorts	[194]
Bladder cancer	Logistic regression, random forest, support vector machine, decision tree	Cancer detection using clinical and biological information	AUC values: 0.82 on the training set, 0.83 on the test set	[195]

### 3.4 Classification and Molecular Subtyping

Recent advancements in AI techniques have the potential to improve tumor characterization, grading, classification, and molecular subtyping, ultimately leading to more accurate and reliable diagnosis and management [197]. A machine learning classifier, known as the “Heidelberg brain tumor classifier,” was developed to achieve accurate, affordable, and fast classification of brain tumors based on genome‐wide DNA methylation profiles [198, 199] (Table 3). The random forest algorithm‐based NEP100 model improved the subtyping of neuroendocrine prostate cancer through multiomics analysis [200]. DL models, such as UNet and CCN, demonstrated high accuracy in the automated segmentation of oral carcinoma [201], brain tumors [202, 203, 204, 205, 206, 207, 208, 209, 210, 211], breast cancer [212], lung cancer [213, 214], prostate cancer [215], renal tumors [216], and thyroid cancer [217] due to their ability to learn complex patterns from medical images . DL algorithms also show promising performance in cancer classification and molecular subtyping using single‐ or multiomics data (e.g., epigenomics, proteomics, radiomics, and single‐cell omics) [218, 219, 220, 221, 222, 223, 224].

**TABLE 3:** Summary of recent studies exploring the potential of artificial intelligence techniques in cancer classification, treatment, and prognostic prediction.

Potential application	Cancer type	Data used for analysis	AI algorithm	Algorithm performance	References
Cancer classification	Central nervous system tumor	Genome‐wide DNA methylation profiles	Random forest	AUC: 0.99; sensitivity: 0.989; specificity: 0.999	[198]
Cancer classification	Brain cancer	Genome‐wide DNA methylation profiles	Random forest	Accurately classify cancer	[199]
Cancer classification	Neuroendocrine prostate cancer	Multiomics data (transcriptomics, single‐cell transcriptomics, and spatial transcriptomics)	Random forest	AUC values: 0.924, 0.986, 0.989, 0.994, 0.995, and 1.000 for validation sets	[200]
Cancer classification	Oral carcinoma	Medical images	Convolutional bidirectional long short‐term memory network	Accuracy rate: 98.47%	[201]
Cancer classification	Brain tumor	MRI images	UNet, Bayesian	Accuracy rate: 97.75%	[202]
Cancer classification	Brain tumor	MRI images	Convolutional neural network	Accuracy rate: 99.16%	[203]
Cancer classification	Brain tumor	Medical images	Convolutional neural network	Accuracy rate: 98.47%; mean intersection over union: 0.8185; average dice coefficient: 0.7; average Hausdorff 95 score: 1.66; precision: 98.55%; sensitivity: 98.40%; specificity: 99.52%	[204]
Cancer classification	Brain tumor	MRI images	Convolutional neural network	Negative predictive value: 89.91%; true negative rate: 92.26%; true positive rate: 93.78%; positive predictive value: 93.60%	[205]
Cancer classification	Brain tumor	MRI images	Convolutional neural network	Accuracy: 0.962; *F*1‐score: 0.965; precision: 0.965; recall: 0.965	[206]
Cancer classification	Brain tumor	MRI images	Convolutional neural network	Accuracy rate: 98.5%	[207]
Cancer classification	Brain tumor	MRI images	Residual neural network; convolutional neural network	Accuracy rate: 98.4%; AUC: 0.999	[208]
Cancer classification	Brain tumor	MRI images	Convolutional neural network	Accuracy rate: 96.01%	[209]
Cancer classification	Brain tumor	MRI images	Deep neural network	Segment and categorize brain tumors more accurately than the existing state‐of‐the‐art mechanisms	[210]
Cancer classification	Glioma	MRI images	Deep learning	Sensitivity: 98.58%; specificity: 99.09%; accuracy rate: 99.1%; dice similarity coefficient: 98.96%	[211]
Cancer classification	Breast cancer	Whole mount slide images, microscopic biopsy images	Convolutional neural network, long short‐term memory	Accuracy rates: 99.17 and 99.90% on the respective datasets	[212]
Cancer classification	Lung cancer	Chest CT images	Convolutional neural network	Accuracy rate: 97.7%; sensitivity: 98.1%; specificity: 97.4%	[213]
Cancer classification	Non‐small‐cell lung cancer	Liquid‐based cytology images	Convolutional neural network	Sensitivity: 0.73; specificity: 0.82; accuracy: 0.78	[214]
Cancer classification	Prostate cancer	MRI images	Deep learning	Accuracy rate: 99.31%; sensitivity: 98.24%; specificity: 98.46%	[215]
Cancer classification	Renal tumor	Contrast‐enhanced CT images	Convolutional neural network	Dice similarity coefficients: 0.83 for tumors larger than 4 cm, 0.65 for tumors smaller than 4 cm; accuracies: 0.77 for tumors larger than 4 cm and 0.68 for tumors smaller than 4 cm	[216]
Cancer classification	Thyroid cancer	Pathological tissue slide images, genetic and protein data	Convolutional neural network, recurrent neural network	Accuracy rate: ∼90%	[217]
Cancer classification	Breast cancer	Epigenomic data	Deep learning	Accuracy rate: 95.85%; recall: 95.96%; precision: 95.85%; *F*1‐score: 95.90%; false positive rate: 1.03%; false negative rate: 4.12%	[218]
Cancer classification	Stomach cancer	Multilayer omics data (Exon, mRNA, mRNA expression data, and DNA methylation profiling)	Recurrent neural network	AUC values: 0.990 for TCGA‐Stomach Adenocarcinoma project, 0.994 for TCGA‐Liver Hepatocellular Carcinoma project	[219]
Cancer classification	Brain tumor	MRI images, radiomics features	Convolutional neural network	Accuracy rate: 95.0%; AUC: 0.92; sensitivity: 88%; specificity: 90%	[220]
Cancer classification	Glioma	Radiomics and clinical features	Deep learning	AUC: 0.98 in an externally validated cohort	[221]
Cancer classification	Brain tumor, leukemia, lung cancer	Gene microarray data	Generative adversarial network	Accuracies: 0.9519 and 0.9644 for brain tumor datasets; 0.9896 and 0.9867 for leukemia datasets; 0.9524 for the lung cancer dataset	[222]
Cancer classification	Clear cell renal cell carcinoma	Radiomics features	Deep learning, support vector machine, K‐nearest neighbor, neural network classifiers	Accuracy rate: 90.9%	[223]
Cancer classification	Lung adenocarcinoma	Radiomics data, MRI images	Convolutional neural networks	AUC: 0.880	[224]
Therapeutic decision optimization	Gastrointestinal stromal tumor	Clinical characteristics	Random forest	Determine the patients who should receive treatment, the optimal duration and treatment outcomes with an AUC of 0.77	[225]
Therapeutic decision optimization	Bladder cancer	Transcriptomic data	Machine learning	Efficiently optimize risk stratification and decision‐making	[226]
Therapeutic decision optimization	Colorectal cancer	Demographic data and staging examination results	ChatGPT	Show high concordance (72.5%) with recommendations posed by traditional multidisciplinary team	[227]
Therapeutic decision optimization	Renal cell carcinoma	Clinicopathological data	Machine learning	Predict the probability of a late recurrence 5 years after surgery with a sensitivity of 0.673, a specificity of 0.807, an accuracy of 0.799, an AUC of 0.740 and a *F*1‐score of 0.609	[228]
Therapeutic decision optimization	Renal cell carcinoma	Clinical data	Gradient boosting machine	Recommend personalized treatment for various clinical situations with an accuracy score of 95%	[229]
Therapeutic decision optimization	Breast cancer	Demographic data, anatomopathological data, and MRI images	Support vector machine	Improve decision‐making processes with a test accuracy of 0.933	[230]
Therapeutic decision optimization	Hepatocellular carcinoma	Pretreatment demographic, clinical, and imaging variables	Random forest	Show good performance in treatment decision with 81% of accuracy for radiofrequency ablation or resection versus not, 88.4% for radiofrequency ablation versus resection, and 76.8% for transarterial chemoembolization or not	[231]
Therapeutic decision optimization	Non‐small cell lung cancer	Clinical characteristics, next‐generation sequencing data	Artificial neural network	Assess the efficacy of first‐line EGFR‐TKI treatment with an accuracy of 0.82 and an AUC of 0.82	[232]
Therapeutic decision optimization	Lung cancer, oropharyngeal cancer	Anatomical information, medical records	Machine learning	Have the potential to guide therapy and reduce the time needed to reach an acceptable plan	[233]
Dynamic treatment monitoring	Breast cancer	MRI images	Deep learning	Achieve AUC values of 0.975 and 0.976 for classifying residual cancer burden scores in the primary cohort and AUC values of 0.923 and 0.910 in external validation cohorts	[234]
Dynamic treatment monitoring	Breast cancer	MRI images	Convolutional neural network	Assess changes in tumor load with an AUC of 0.76	[235]
Dynamic treatment monitoring	Melanoma, lung cancer	Plasma whole genomic data	Support vector machine	Monitor cancer progression following immunotherapy with an AUC of 0.998	[236]
Dynamic treatment monitoring	Lung cancer	Clinical data	Explainable artificial intelligence, convolutional neural network	Efficiently track tumor progression following radiotherapy	[237]
Dynamic treatment monitoring	Breast cancer	Longitudinal MRI spatial habitat radiomics, transcriptomics, single‐cell transcriptomics	XGBoost	Predict pathological complete response to immunotherapy with a specificity of 88% and a positive predictive value of 93%	[238]
Dynamic treatment monitoring	Breast cancer	Clinical and imaging data	Vision transformer	Monitor pathological complete response to chemotherapy with an AUC of 0.813	[239]
Dynamic treatment monitoring	Breast cancer	MRI images, radiomics data	XGBoost	Predict pathological complete response to chemotherapy with an AUC of 0.89	[240]
Dynamic treatment monitoring	Esophageal squamous cell carcinoma	CT images, radiomics data	Transformer	Predict pathological complete response to immunotherapy combined with chemotherapy with accuracies of 0.83–0.91 and AUC values of 0.83–0.92	[241]
Dynamic treatment monitoring	Rectal cancer	Radiology images	Deep learning	Predict pathological complete response to chemoradiotherapy with an AUC of 0.90	[242]
Dynamic treatment monitoring	Rectal cancer	MRI images	Gradient boosting machine	Identify complete response to radiation with a sensitivity of 90% and a specificity of 86%	[243]
Dynamic treatment monitoring	Rectal cancer	Laboratory data	Machine learning	Predict pathological complete response to chemoradiotherapy with AUC values of 0.86 and 0.83 in the internal and external validations set	[244]
Dynamic treatment monitoring	Esophageal cancer	CT radiomics, dosimetric characteristics	XGBoost	Predict the response to chemoradiation with an accuracy of 0.708 and an AUC of 0.541	[245]
Dynamic treatment monitoring	Cervical cancer	Multimodal data	Support vector machine	Predict chemoradiotherapy response with an AUC value of 0.86	[246]
Dynamic treatment monitoring	Gynecologic cancer	Multimodal data	Machine learning	Predict complete response to radiotherapy	[247]
Dynamic treatment monitoring	Prostate cancer	MRI images	Random forest, decision tree, support vector machine	Predict radiotherapy response with AUC values of 0.722, 0.685, and 0.5	[248]
Dynamic treatment monitoring	Melanoma	Histology data, clinicodemographic characteristics	Machine learning	Predict immunotherapy response with AUC values of 0.800 and 0.805	[249]
Dynamic treatment monitoring	Gastric cancer	Radiomics, clinicopathological, and imaging data	Convolutional neural network	Predict immunotherapy response with an AUC of 0.783	[250]
Dynamic treatment monitoring	Gastric cancer	Transcriptomic and digital pathology data	ResNet	Predict immunotherapy response	[251]
Dynamic treatment monitoring	Hepatocellular carcinoma	CT images, radiomics, clinical data	ResNet, convolutional neural network	Predict immunotherapy response with AUC values of 0.96 and 0.88	[252]
Dynamic treatment monitoring	Non‐small cell lung cancer	Radiology text reports	Deep learning	Predict immunotherapy response	[253]
Overcoming treatment resistance	Brain cancer, colon cancer, ovarian cancer, breast cancer	Gene expression profiles	Machine learning	Identify drug resistance signatures	[254]
Overcoming treatment resistance	Breast cancer	Drug response data	Artificial neural network	Identify genetic mutations associated with drug resistance	[255]
Overcoming treatment resistance	Bladder cancer	Multiomics data	Machine learning	Identify drug resistance signatures	[256]
Overcoming treatment resistance	Lung cancer	CT images	Convolutional neural network	Predict *EGFR* mutation relevant to drug resistance	[257]
Overcoming treatment resistance	Nasopharyngeal carcinoma	Radiomics, plasma metabolomics profiles	XGBoost	Reveal the relationship between dysregulation of plasma lipoprotein and chemotherapy resistance	[258]
Overcoming treatment resistance	Breast cancer	Gene expression profiles	Transformer	Identify key genes (*SNHG25* and *SNCG*) associated with chemotherapy resistance	[259]
Overcoming treatment resistance	Colorectal cancer	Gene expression profiles	Machine learning	Identify the key gene (*EIF5A*) associated with radioresistance	[260]
Overcoming treatment resistance	Gastric cancer	Gene expression data	Machine learning	Identify key genes (*CDH6*, *EGFLAM*, and *RASGRF2*) associated with immunotherapy resistance	[261]
Overcoming treatment resistance	Esophageal squamous cell carcinoma	Proteomic data	Support vector machine, random forest	Identify critical spatial protein features linked to immunotherapy resistance	[262]
Overcoming treatment resistance	Breast cancer	Transcriptomic data	K‐nearest neighbor	Identify the association between RTK and ER oncogenic pathways and endocrine resistance	[263]
Overcoming treatment resistance	Melanoma	Transcriptomic data	Machine learning	Identify the involvement of chemotaxis‐associated ligand–receptor interactions in immunotherapy resistance	[264]
Overcoming treatment resistance	Gastric cancer	Multiomics data	Machine learning	Unveil the specific cellular interplay (TGFB1–HSPB1 and LTF–S100A14) underlying chemotherapy resistance	[265]
Overcoming treatment resistance	Ovarian cancer	Histopathological data	Machine learning	Reveal the relationship between intratumor heterogeneity and chemotherapy resistance	[266]
Overcoming treatment resistance	Gastric cancer	Transcriptomic data	Support vector machine, random forest, neural network, XGBoost, decision tree, K‐nearest neighbor	Reveal the relationship between tumor microenvironment cellular heterogeneity and chemotherapy resistance	[267]
Overcoming treatment resistance	Hepatocellular carcinoma	Transcriptomic data	Graph neural network	Identify potential therapeutic candidates	[268]
Overcoming treatment resistance	Melanoma, lung cancer	Genomic and transcriptomic data	Machine learning	Identify immune inhibitory receptors as potential therapeutic targets	[269]
Overcoming treatment resistance	Lung adenocarcinoma	Transcriptomic data	Deep neural network	Identify ABCC2 as a promising therapeutic target	[270]
Overcoming treatment resistance	Colorectal cancer	Metabolomics data	Machine learning	Identify hexokinase as a promising therapeutic target	[271]
Overcoming treatment resistance	Acute myeloid leukemia	Transcriptomics data, single‐agent response profiles	XGBoost	Identify personalized regimens to overcome treatment resistance	[272]
Overcoming treatment resistance	Gastric cancer	Multiomics data	Transformer	Accurately predict treatment response	[273]
Toxicity management and survivorship	Different types of cancer	Clinical, laboratory, and echocardiographic data	Machine learning	Predict chemotherapy‐induced cardiotoxicity	[274]
Toxicity management and survivorship	Breast cancer	Clinical and demographic data	Cox regression, XGBoost	Predict chemotherapy‐induced toxicity	[275]
Toxicity management and survivorship	Renal cell carcinoma, colorectal cancer, gastrointestinal stromal tumor, thyroid cancer, soft tissue sarcoma	Clinical data	ResNet, XGBoost	Predict chemotherapy‐induced hand‐foot skin reaction	[276]
Toxicity management and survivorship	Colorectal cancer	Clinical data	Random forest	Predict chemotherapy‐induced toxicity	[277]
Toxicity management and survivorship	Lung cancer, liver cancer	Medical records	XGBoost	Predict immunotherapy‐induced hypothyroidism	[278]
Toxicity management and survivorship	Melanoma, lung cancer, genitourinary cancer	CT images	Convolutional neural network, transformer	Predict immunotherapy‐induced pneumonitis	[279]
Toxicity management and survivorship	Lung cancer	CT and radiation dose images	ResNet	Predict radiation pneumonitis	[280]
Toxicity management and survivorship	Nasopharyngeal carcinoma	Clinical data	Machine learning	Predict radiation‐induced oral mucositis	[281]
Toxicity management and survivorship	Breast cancer	Clinical data	Neural network, random forest, XGBoost, logistic regression	Predict radiation dermatitis	[282]
Toxicity management and survivorship	Rectal cancer	MRI images	Vision transformer, convolutional neural network	Predict overall survival with a concordance index of 0.82 and a hazard ratio of 2.3 in the external test set	[283]
Toxicity management and survivorship	Colorectal cancer	Histopathology images	Convolutional neural network	Predict 5‐year survival with AUCs of 0.70 and 0.69 for two validation datasets	[284]
Toxicity management and survivorship	Colorectal cancer	Histopathology images	Long short‐term memory	Achieve a hazard ratio of 2.3 and an AUC of 0.69 for outcome prediction	[285]
Toxicity management and survivorship	Head and neck cancer, colorectal cancer	Histopathology images	Graph neural network	Show high accuracy in predicting patient outcome	[286]
Toxicity management and survivorship	Colorectal cancer	Immune cell patterns	Deep learning	Show high prognostic capabilities	[287]
Toxicity management and survivorship	Ocular cancer, rectal cancer	Electronic health records	Explainable artificial intelligence	Determine the prognostic contribution of clinical markers	[288]
Toxicity management and survivorship	Breast cancer	Clinicopathological and MRI characteristics	XGBoost	Achieve AUC values of 0.805, 0.803, and 0.818 for prognostic prediction in three cohorts	[289]
Toxicity management and survivorship	Breast cancer	Mitochondrial and lysosomal gene expression data	Coxboost, support vector machine	Achieve AUC values exceeding 0.647 for prognostic prediction in various datasets	[290]
Toxicity management and survivorship	Breast cancer	TCGA, METABRIC and GEO datasets	Machine learning (random survival forest, elastic network, CoxBoost, and support vector machine)	Reach an average concordance index of 0.79 for prognostic prediction	[291]
Toxicity management and survivorship	Melanoma	Single cell transcriptome data and medical variables	Machine learning (LightGBM, CatBoost, random forest, and ensemble learning)	Achieve AUC values of 0.9957, 0.9939, 0.9681, and 0.9975 for outcome prediction in the testing cohort	[292]
Toxicity management and survivorship	Lung adenocarcinoma	CT and histopathology images	Support vector machine	Achieve concordance indices of 0.744, 0.719, and 0.711 for the prediction of disease‐free survival in one training dataset and two testing datasets	[293]
Toxicity management and survivorship	Lung cancer	Radiomics data and clinical parameters	Machine learning	Demonstrate superior performance in overall survival prediction, with time‐dependent AUC values above 0.75 and concordance indices of 0.87 and 0.76 for the training and testing sets	[294]
Toxicity management and survivorship	Hepatocellular carcinoma	Demographic, clinical, pathological, and laboratory data	Machine learning (K‐nearest neighbor and support vector machine)	Reach accuracy rates above 87% for 3‐year overall survival prediction	[295]
Toxicity management and survivorship	Pancreatic ductal adenocarcinoma	Serum biomarker data and prognosis information	Deep learning	Excel in prognosis prediction with concordance indices of 0.738 and 0.724 on training and validation sets	[296]
Toxicity management and survivorship	Bladder cancer	Clinical and histopathology data	LightGBM	Show good performance in predicting 5‐year cancer‐specific mortality with concordance indices of 0.723 and 0.791 in internal and external validation sets	[297]
Toxicity management and survivorship	Bladder cancer	Clinical data	Random survival forest, elastic network	Achieve concordance indices of 0.683, 0.688, and 0.666 for prognostic prediction in training, internal and external test sets	[298]
Toxicity management and survivorship	Breast cancer	Pathomics data	Weakly supervised learning	Achieve a concordance index of 0.710 for survival prediction	[299]
Toxicity management and survivorship	Hepatocellular carcinoma	Contrast‐enhanced MRI and pathologic images	Convolutional neural network, densely connected convolutional network, vision transformer, swin transformer	Achieve time‐dependent AUC values of 0.83, 0.81, and 0.78 for 3‐year progression‐free survival in training, internal, and external test sets	[300]
Toxicity management and survivorship	Papillary thyroid carcinoma	Preoperative ultrasound images	Deep learning	Efficiently predict disease‐free survival with a hazard ratio of 16.49	[301]
Toxicity management and survivorship	Castration‐resistant prostate cancer	Digital histopathological images and clinical parameters	Deep learning	Achieve significant correlations between AI‐based risk score and shorter metastasis‐free survival (hazard ratio, 1.72; *p* < 0.005) and overall survival (hazard ratio, 1.41; *p *= 0.02)	[302]
Toxicity management and survivorship	Prostate cancer	Digitized histopathology images and clinical variables	Multimodal artificial intelligence	Achieve a hazard ratio of 6.46 and a 95% confidence interval of 1.44–28.9 for overall survival prediction	[303]
Toxicity management and survivorship	Breast cancer	Gene expression and survival data, RNA‐binding protein information	Machine learning	Show better prognostic performance than 106 established signatures	[304]
Toxicity management and survivorship	Esophageal cancer	Transcriptome data	Machine learning	Exhibit good prognostic performance with AUC values exceeding 0.7 in two external validation sets	[305]
Toxicity management and survivorship	Colorectal cancer	Single cell RNA sequencing data, spatial transcriptome data	Machine learning (LASSO regression and random survival forest)	Exhibit good prognostic performance with an average concordance index of 0.729	[306]
Toxicity management and survivorship	Colorectal cancer	Gene expression data	Machine learning	Achieve AUC values exceeding 0.6132 in predicting 1‐, 2‐, and 3‐year survival	[307]
Toxicity management and survivorship	Gastric cancer	Transcriptomic and clinical characteristic data	Machine learning	Show good prognostic performance with a hazard ratio of 1.145	[308]
Toxicity management and survivorship	Hepatocellular carcinoma	Transcriptomic and clinical data	Machine learning	Outperform 22 existing models in predicting patient outcomes	[309]
Toxicity management and survivorship	Pancreatic cancer	Gene expression data	Machine learning	Achieve AUC values exceeding 0.566 in predicting 1‐, 3‐, and 5‐year survival	[310]
Toxicity management and survivorship	Ovarian cancer	Transcriptomic and clinical data	Machine learning	Exhibit reliable predictive potential in predicting 1‐ (AUC = 0.702), 3‐ (AUC = 0.640), and 5‐year survival (AUC = 0.618)	[311]
Toxicity management and survivorship	Prostate cancer	mRNA microarray data	Machine learning	Exhibit excellent predictive performance in predicting 1‐ (AUC = 0.754), 3‐ (AUC = 0.776), and 5‐year overall survival (AUC = 0.706)	[312]

Accurate tumor classification is essential for reliably and reproducibly tracking tumor size and volume over time. AI‐assisted automated cancer segmentation can be integrated into clinical oncological imaging workflows, overcoming the time constraints associated with manual size comparisons [313, 314]. AI techniques help identify new imaging biomarkers that serve as indicators for cancer subtypes and disease progression [315]. Furthermore, AI models can incorporate multimodal data to enhance cancer classification and subtyping [316].

## 4 AI‐Driven Precision Therapy and Dynamic Management

### 4.1 Therapeutic Decision Optimization

AI has the ability to optimize the decision‐making process. For instance, optimal policy trees, an interpretable AI‐based method, could identify patients with gastrointestinal stromal tumor who should receive adjuvant imatinib, determine the optimal treatment duration, and assess the benefits of this treatment [225]. Machine learning‐based autophagy‐related prognostic signature was correlated with decreased sensitivity to immunotherapy in breast cancer and thus represented a practical tool to optimize decision‐making [226]. ChatGPT‐generated treatment recommendations showed strong concordance (72.5%) with those posed by traditional multidisciplinary team in CRC [227]. The clinical decision support system (CDSS) utilizes AI‐driven data integration to deliver evidence‐based, real‐time clinical recommendations in oncology [317]. A machine learning‐based CDSS showed outstanding performance in identifying patients with renal cell carcinoma (RCC) who were at high risk of late recurrence, thus emphasizing the need for long‐term follow‐up and the development of patient‐specific treatment strategies [228]. RCC‐Supporter, another machine learning‐driven CDSS system, recommended individualized therapy for RCC patients, taking into account their diverse clinical statuses [229]. SVM algorithm‐based CDSS (CureMate) demonstrated high accuracy and effectiveness in selecting the optimal first treatment strategy in breast cancer [230]. A machine learning‐based HCC‐CDSS model demonstrated promising performance in recommending initial treatment options and predicting overall survival in HCC patients [231]. A CDSS based on ANN was developed to support clinical decisions in non‐small cell lung cancer (NSCLC) patients by distinguishing the efficacy of EGFR‐TKI therapy [232]. Valdes et al. [233] developed a CDSS system that helped clinicians efficiently match new patients with historically approved treatment plans, facilitating the selection of radiotherapy in lung and oropharyngeal cancers.

### 4.2 Dynamic Treatment Monitoring

AI has become an important tool for longitudinal tracking of tumor progression following treatment. A multitask AI system was developed to predict residual cancer burden scores in breast cancer, thereby supporting clinical decision‐making during neoadjuvant chemotherapy [234]. A CNN‐based DL model that integrated MRI data showed good performance in assessing changes in tumor load in breast cancer after neoadjuvant chemotherapy treatment [235]. A machine learning‐based detection platform monitored tumor burden in advanced melanoma and lung cancer following neoadjuvant immunotherapy by tracking changing dynamics of circulating tumor DNAs [236]. Explainable AI (XAI) methods, such as Guided Backpropagation and DeepLIFT, could assess the reliability of a DL‐based tumor tracking model for lung cancer patients during radiation delivery [237]. AI helps identify changes in tumor size, volume, or growth, allowing clinicians to define whether the tumor shrinks due to treatment efficacy or develops because of treatment resistance.

Accurate prediction of tumor response to therapeutic regimens enables personalized treatment for cancer. With numerous radiomics/radiopathomics data, AI algorithms, such as XGBoost, boosted decision tree, Vision Transformer, and 3D‐ResNet, showed potential in predicting pathological complete response to neoadjuvant chemotherapy in breast cancer [238, 239, 240] and esophageal squamous cell carcinoma (ESCC) [241]. AI techniques showed favorable performance for predicting pathological complete response to chemoradiotherapy in rectal cancer [242, 243, 244], esophageal cancer [245], and cervical cancer [246]. Image‐based machine learning could efficiently predict radiotherapy response in oligometastatic gynecologic cancer [247] and prostate cancer [248]. DL models were used to predict clinical response to checkpoint blockade immunotherapy in advanced melanoma [249], gastric cancer [250, 251], HCC [252], and NSCLC [253]. AI‐based algorithms hold significant promise for real‐time monitoring of treatment response, leading to improved clinical outcomes in cancer patients.

### 4.3 Overcoming Treatment Resistance

Increasing evidence indicates that AI can analyze genetic mutations, drug metabolism, and drug sensitivity to identify patients at risk of developing resistance to specific treatments, facilitating timely and effective interventions [254, 255, 256, 257]. Moreover, AI techniques can decipher the mechanisms responsible for treatment resistance in cancer patients. For instance, AI algorithms (e.g., transformer, SVM, and random forest) identified key genes/proteins associated with treatment resistance in nasopharyngeal carcinoma (NPC) [258], breast cancer [259], CRC [260], gastric cancer [261], and ESCC [262]. Machine learning algorithms provided new insights into cell pathways underlying treatment resistance in breast cancer [263], melanoma [264], and gastric cancer [265]. The correlation between tumor heterogeneity and cancer treatment resistance was also explored through employment of machine learning‐based platforms [266, 267]. Furthermore, AI techniques have the potential to discover novel therapeutic targets to overcome treatment resistance. Graph neural network was used to predict the interactions between genes and chemotherapeutic agents and identify potential therapeutic candidates for HCC intervention [268]. A machine learning‐based bioinformatics pipeline identified immune inhibitory receptors as prospective therapeutic targets for cancer immunotherapy [269]. Integrating transcriptomics data with machine learning identified the ferroptosis regulator ABCC2 as a potential therapeutic target to overcome cisplatin resistance in lung adenocarcinoma [270]. A machine learning‐based metabolic modeling approach screened hexokinase as a contributing factor to drug resistance in CRC and as a promising target [271]. In addition, AI offers a rational means to customize appropriate regimens to reduce treatment resistance. A machine learning‐based computation model identified personalized drug combination strategies that effectively targeted treatment‐resistant cancer cells in patients with acute myeloid leukemia [272]. A transformer‐based DL model that incorporated multimodal data was likely to improve the management of patients with HER2‐positive gastric cancer by assessing treatment response [273]. Collectively, AI techniques are expected to revolutionize cancer drug resistance research through prediction of resistance patterns, interpretation of underlying molecular mechanisms, discovery of novel therapeutic targets, and optimization of anticancer treatment strategies.

### 4.4 Toxicity Management and Survivorship

AI technology is advancing toxicity assessments in anticancer therapies. Machine learning algorithms predicted chemotherapy‐induced toxicity, such as cardiotoxicity and hand‐foot skin reaction, and guided treatment decisions for cancer patients [274, 275, 276, 277]. AI algorithms, including XGBoost and CNN, predicted the likelihood of immune checkpoint inhibitor‐induced hypothyroidism and pneumonitis in cancer patients, providing guidance for risk management and individualized therapy [278, 279]. AI techniques could also predict the occurrence of radiation‐induced oral mucositis, pneumonitis, and dermatitis in cancer patients [280, 281, 282]. AI‐powered timely identification of patients experiencing adverse events enables clinicians to customize treatment strategies and reduce the risk of toxicity.

AI can assess and interpret “multifactor” data from patient evaluations, offering more accurate insights into patient survival and prognosis [318]. Prognostic AI models for cancer have been developed across multiple data modalities, encompassing multiplex imaging, histopathology, and clinical characteristics [283, 284, 285, 286, 287]. XAI decoded the outcome of solid cancer patients based on clinical markers derived from multimodal real‐word data including clinical records, image‐derived body composition, and mutational tumor profiles [288]. XAI determined the prognostic contribution of each marker at the patient level and revealed prognostic interactions between markers. Machine learning platforms, including XGBoost, survival‐SVM, random survival forest, and CoxBoost, have shown prognostic potential for patients with breast cancer [289, 290, 291], melanoma [292], lung cancer [293, 294], HCC [295], and PDAC [296]. Particularly, two machine learning models (LightGBM and RSF+Enet[alpha = 0.8] model) integrating clinical data exhibited superior performance in prognostic prediction compared with existing models in bladder cancer [297, 298]. DL models incorporating radiomics/radiopathomics could effectively forecast patient outcomes in breast cancer [299], HCC [300], papillary thyroid carcinoma (PTC) [301], and prostate cancer [302, 303], enabling data‐driven postoperative treatment planning. Further work is necessary to assess the clinical utility of these AI‐assisted prognostic models. The features used by AI models to infer clinical outcomes should be clearly interpreted.

In addition to directly predicting prognosis, many studies have explored AI's ability to identify predictive biomarkers for patient outcomes. Reportedly, machine learning‐assisted prognostic models integrating cancer‐associated genes/noncoding RNAs were established for outcome prediction in breast cancer [304], esophageal cancer [305], CRC [306, 307], gastric cancer [308], HCC [309], pancreatic cancer [310], ovarian cancer [311], and prostate cancer [312]. Collectively, AI may be a promising option for improving the prognosis and quality of life of cancer patients. Integration of AI techniques into cancer prognosis would contribute to more effective cancer treatments. However, clinical trials comparing AI programs with standard prognostic models are imperative to assess their real‐world value in cancer care.

## 5 Translational Evidence: From Bench to Real‐World Impact

AI approaches are transforming the integration and interpretation of real‐world oncological data and are starting to influence various aspects of clinical cancer management [319]. Image‐based AI frameworks facilitated the early diagnosis of breast cancer in the mammography screening [320], brain tumors using MRI images [321], skin cancer using dermoscopy [322], and colon cancer through histopathological images [323]. Notably, AI‐based diagnostic tools assist in alleviating the workload of screen‐reading without increasing the number of false positives. Previous studies demonstrated the high accuracy of AI prediction models in automated cancer classification in the real‐world context [324, 325]. Importantly, AI tools could improve decision‐making processes and symptom management for cancer patients [326, 327]. They also showed outstanding performance in predicting treatment response and prognosis in cancer patients by leveraging digital histopathological data [328, 329, 330, 331]. In the aforementioned studies, ChatGPT, CNN, and ensemble models were used for data analysis. ChatGPT can streamline the detection workflows, reduce errors, and improve consistency in pathology assessments [332]. However, its ability to handle images and other nontext data are limited. Since images and sound data are critical inputs in clinical settings, GhatGPT cannot directly process these medical data to generate diagnostic recommendations. CNN shows human‐level performance in computer vision tasks; however, it cannot encode spatial information in the inputs, such as orientation, position, and size [65]. Moreover, CNN requires large training data and exhibits low performance in overlapped images. Ensemble machine learning methods leverage computational functions to combine diverse learning models, which mitigates the limitations of individual algorithms [333]. The drawbacks of ensemble models include high computational costs, high bias, vulnerability to overfitting, and difficulty in model interpretability.

AI has the potential to revolutionize the way by which cancer diagnoses and treatment recommendations are delivered to patients. Nevertheless, implementing AI in clinical oncology faces numerous challenges [334]. It is uncertain whether patients prefer AI‐based diagnoses to those from doctors. In automated diagnostic settings, it remains unclear whether patients seek comfort and clarification from nonexpert operators. Developing oncological AI platforms requires accessing patient data, which raises questions about data security and privacy protection. Furthermore, patients may lack trust in AI systems to handle their diagnosis and treatment information appropriately. All these issues should be resolved before the widespread application of AI in clinical oncology.

### 5.1 Key Preclinical Validation Studies

It is well established that successful clinical trials are built upon thorough preclinical validation studies. The efficacy and reliability of AI algorithms in cancer care are evaluated in preclinical studies that involve large‐scale biomedical datasets, medical imaging, and clinical information. For instance, AIEgen‐Deep, a DL algorithm‐driven model, showed significant accuracy in identifying cancer cell morphology and differentiating between healthy cells and cancer cells [335]. This tool might facilitate early cancer diagnosis and improve clinical outcomes in cancer patients. DL‐based semantic segmentation models could detected head and neck tumor from the multimodal data with high sensitivity and specificity in a preclinical study [336]. A novel semi‐automatic machine learning approach accurately detected brain metastatic lesions and quantified metastatic burden in preclinical models [337]. Machine learning algorithms (e.g., SVM and mRMR) were efficient in assessing breast cancer progression through photoacoustic spectroscopy in a mouse model [338]. A transcriptome‐based tool (Pancreas‐View) was developed using preclinical models and random forest approach [339]. This tool could accurately predict patient sensitivity to adjuvant chemotherapy in PDAC patients. Machine learning algorithms, including ANN, SVM, and random forest, efficiently evaluated the predictive potential of circulating biomarkers for metastatic outcomes in breast cancer animal models following neoadjuvant treatment [340]. These preclinical models may help identify treatment failures that should be avoided in clinical practice.

Preclinical AI studies not only pave the way for subsequent clinical trials but also foster the effective integration of AI into clinical oncology workflows. It should be noted that preclinical studies confront many challenges. The quality and integrity of data remarkably affect the performance of AI models. The availability of high‐quality datasets are essential to maximize data value. Moreover, the inherent “black‐box” nature of AI algorithms makes their decision‐making process opaque, raising concerns about reliability in clinical settings. Consequently, it is necessary to gain a better understanding of the decision‐making basis of AI models and increase clinicians’ trust in AI tools. By overcoming current challenges, AI is expected to play an increasingly significant role in the field of clinical cancer.

### 5.2 Landmark Clinical Trials

Clinical trials serve to assess the efficacy of AI technologies in cancer detection and explore their potential integration into clinical decision‐making processes (Table 4). In randomized controlled trials, endoscopy‐guided AI detection systems (e.g., CADe, CADopt, EW10‐EC02, LCA, and CNN) improved the diagnosis of adenoma [341, 342, 343, 344, 345, 346] and esophageal cancer [347, 348]. AI‐powered image analysis tools efficiently detected glioma [349], breast cancer [350], and prostate cancer [351, 352] using MRI data. An AI‐based clinical decision support tool achieved high diagnostic accuracy in detecting cutaneous melanoma through dermoscopic image analysis, assisting physicians in detecting skin lesions for melanoma [353]. Although many AI tools show promising utility, most of them remain insufficiently developed for clinical use. Notably, several AI systems did not result in improved cancer detection [354, 355, 356]. Therefore, AI‐driven clinical tools must undergo thorough training and rigorous validation of their universality and robustness before being adopted into patient clinical care.

**TABLE 4:** Summary of clinical trials evaluating the effect and performance of artificial intelligence technologies in oncology.

Type of study	Clinical trial ID	Phase	Status	Cancer type	Number of subjects	Objectives	AI task	AI method	Preliminary findings	Reference
Single‐center pragmatic randomized controlled trial	NCT05963724	Not applicable	Completed	Colorectal cancer	1100	Evaluate the sensitivity of computer‐aided detection compared to standard colonoscopy in detecting colon polyps	Cancer detection using histopathologic findings	CADe	Have a higher adenoma detection rate (42.5%) than the traditional colonoscopy (34.4%)	[341]
Randomized controlled trial	NCT05236790	Phase 2	Completed	Colorectal cancer	467	Assess the performance of the AI system for detection and classification of polyp histology	Cancer detection using histopathologic findings	CADe	Have a higher adenoma detection rate (49.3%) than the standard colonoscopy (38.2%)	[342]
Three‐arm prospective randomized colonoscopy study	NCT05133544	Not applicable	Completed	Colorectal cancer	682	Compare the adenoma detection rates among endocuff‐AI‐assisted colonoscopy, AI‐assisted colonscopy, and conventional colonoscopy	Cancer detection using histopathologic findings	Endocuff‐AI	Have a higher adenoma detection rate (58.7%) than AI alone (53.8%)	[343]
Multicenter, prospective randomized trial	NCT04979962	Not applicable	Completed	Colorectal cancer	1031	Investigate the superiority of colorectal polyp detection using computer‐assisted colonoscopy compared to conventional colonoscopy	Cancer detection using histopathologic findings	CAD‐EYE	Lead to a higher adenoma per colonoscopy (0.99) compared with conventional colonoscopy (0.85)	[344]
Multicenter, randomized, noninferiority tandem study	NCT05323279	Not applicable	Completed	Colorectal cancer	685	Assess the effects of an AI system on colonoscopy quality of novice endoscopists	Cancer detection using histopathologic findings	AI‐assisted system	Have a lower adenoma miss rate (18.82%) than the control novice group (43.69%)	[345]
Randomized trial	Not applicable	Not applicable	Completed	Colorectal cancer	800	Compare adenoma detection rate between linked‐color imaging (LCI) with AI and LCI alone	Cancer detection using histopathologic findings	LCA	Show a higher adenoma detection rate (58.8%) than the linked‐color imaging method (43.5%)	[346]
Multicenter, tandem, double‐blind, randomized controlled trial	ChiCTR2100052116	Not applicable	Completed	Oesophageal squamous cell carcinoma	5934	Assess the auxiliary diagnostic performance of the AI system in a real clinical setting	Cancer detection using histopathologic findings	AI‐assisted endoscopy	Show a lower per‐patient miss rate (1.9%) than the routine method using a computerized random number generator	[347]
Prospective, randomized controlled trial	ChiCTR2100044126	Not applicable	Completed	Esophageal squamous cell carcinoma	3117	Assess the efficacy of CNN‐based system in improving cancer detection rate in clinical practice	Cancer detection using histopathologic findings	CNN‐assisted endoscopy	Have a higher cancer detection rate (1.8%) than unassisted endoscopy (0.9%)	[348]
Randomized controlled trial	Not applicable	Not applicable	Completed	Glioma	146	Investigate the application of machine learning‐based MRI radiomics in predicting lower‐grade glioma	Cancer detection using MRI radiomics	Machine learning	Show an excellent performance with an AUC value of 0.925 and accuracy of 0.882 in the training cohort and an AUC value of 0.886 and accuracy of 0.864 in the testing cohort	[349]
Randomized controlled trial	NCT04832594	Not applicable	Completed	Breast cancer	559	Determine the ability of an AI pipeline to identify patients who would benefit from supplemental MRI	Cancer detection using MRI images	AISmartDensity	Show higher efficiency in terms of cancers detected per 1,000 MRI examinations compared with traditional breast density measures (64 versus 16.5)	[350]
International, paired, non‐inferiority, confirmatory study	NCT05489341	Not applicable	Completed	Prostate cancer	9,129	Investigate the performance of the AI system at detecting clinically significant prostate cancer on MRI in comparison with radiologists	Cancer detection using MRI images	PI‐CAI	Show better predictive performance (AUC=0.91) than the pool of 62 radiologists (AUC=0.86)	[351]
Randomized controlled trial	NCT06362291	Not applicable	Completed	Prostate cancer	380	Compare the cancer detection rates of AI‐cTB and routine cTB	Cancer detection using MRI images	AI‐cTB	Have a higher cancer detection rate (58.64%) than the cognitive fusion MRI‐guided targeted biopsy (46.56%)	[352]
Prospective real‐life clinical trial	NCT05172232	Not applicable	Completed	Cutaneous melanoma	228	Determine the diagnostic performance of an AI‐based clinical decision support tool for cutaneous melanoma detection	Cancer detection using histopathologic findings	AI‐based clinical decision support tool	Achieve an AUC of 0.960, a sensitivity of 95.2% and a specificity of 84.5%	[353]
International, multicenter, randomized controlled trial	NCT04909671	Not applicable	Completed	Colorectal cancer	456	Evaluate the performance of AI‐assisted colonoscopy in cancer detection	Cancer detection using histopathologic findings	CADe	Achieve comparable efficiency to white light endoscopy	[354]
Prospective, single‐center, exploratory, and randomized controlled trial	Not applicable	Not applicable	Completed	Esophageal squamous cell carcinoma	320	Determine the ability of AI in improving cancer detection in a clinical setting	Cancer detection using histopathologic findings	AI diagnostic support system	Achieve a cancer detection rate (47%) comparable to that of endoscopists (45%)	[355]
Prospective, randomized controlled trial	NCT05178095	Not applicable	Completed	Colorectal cancer	286	Evaluate adenoma and polyp detection rate of AI‐aided colonoscopy	Cancer detection using histopathologic findings	AI‐C	Achieve a cancer detection rate (41%) comparable to that of conventional colonoscopy (42%)	[356]
Randomized controlled trial	Not applicable	Not applicable	Completed	Breast cancer	341	Assess the performance of a deep learning framework in predicting lymphovascular invasion	Cancer progression prediction using MRI images and histopathologic findings	MM‐Net, PCMM‐Net	Achieve AUC values of 0.774 and 0.843	[357]
Non‐randomized, single‐center clinical trial	14323711	Not applicable	Completed	Breast cancer	190	Assess the efficacy of an AI‐assisted workflow for detecting cancer metastases in sentinel lymph nodes	Cancer progression prediction using histopathologic findings	Visiopharm	Lead to cost and time savings and up to 30% improved sensitivity in the detection of cancer metastases	[358]
Multi‐center, randomized crossover, multi‐reader evaluation study	Not applicable	Not applicable	Completed	Breast cancer, lung cancer, melanoma, colorectal cancer	50	Evaluate the performance of an AI system for brain metastasis segmentation	Cancer progression prediction using MRI images	BMSS	Yield a median Dice similarity coefficient of 0.91 in brain metastasis delineation	[359]
Randomized controlled trial	Not applicable	Not applicable	Completed	Lung cancer	141	Assess the efficacy of a machine learning model in differentiating between bone metastases and benign bone lesions	Cancer progression prediction using SPECT/CT images	LASSO regression, support vector machine	Achieve AUC values of 0.939 and 0.925 in differentiating between bone metastases and benign bone lesions for the training and testing set	[360]
Randomized controlled trial	Not applicable	Not applicable	Completed	Prostate cancer	211	Evaluate the efficacy of a deep learning‐based model in early diagnosis of bone metastasis	Cancer progression prediction using MRI images and pathological features	Deep transfer learning	Yield AUC values of 0.89 and 0.85 in predicting the risk of bone metastases for the training and validation sets	[361]
Prospective multicenter study	ChiCTR1900025592	Not applicable	Completed	Papillary thyroid carcinoma	488	Determine the performance of an AI‐assisted method for predicting cervical lymph node metastasis	Cancer progression prediction using ultrasound videos	MMD‐DL	Achieve AUC values of 0.85 and 0.81 in the test and validation cohorts; improve the average diagnostic accuracy and sensitivity in predicting cervical lymph node metastasis	[362]
Randomized controlled trial	Not applicable	Not applicable	Completed	Ovarian cancer	849	Assess the accuracy of a deep learning radiomics nomogram for predicting the malignant risk of cancer	Cancer progression prediction using ultrasound imaging	DLR_Nomogram	Show superior performance in predicting the risk of cancer malignancy, with AUC values of 0.985 and 0.928 for the training and testing sets	[363]
Randomized controlled trial	Not applicable	Not applicable	Completed	Nasopharyngeal carcinoma	219	Assess the performance of a machine learning approach in predicting radiation‐induced hypothyroidism	Treatment response prediction using clinical features, dose‐volume histograms, radiomics, and dosiomics features	LASSO regression, XGBoost	Achieve an AUC value of 0.842 in the test cohort; have superior clinical utility within the threshold probability range of 1% to 79%	[364]
Phase 2 clinical trial	Not applicable	Not applicable	Completed	Non‐small cell lung cancer	45	Evaluate the effectiveness of a deep learning model in predicting therapeutic response to neoadjuvant immunotherapy	Treatment response prediction using CT images	Deep learning	Show superior predictive performance with an AUC value of 0.820	[365]
Randomized controlled trial	Not applicable	Not applicable	Completed	Hepatocellular carcinoma	114	Explore the performance of deep learning (convolutional neural networks) in predicting overall survival	Survival outcome prediction using CT images and clinical parameters	Clinical cox‐regression model	Show excellent performance in overall survival prediction with a concordance index of 0.74	[366]
TPExtreme clinical trial	NCT02268695	Phase 2	Completed	Head and neck squamous cell carcinoma	526	Compare the predictive power of machine learning models versus conventional survival models	Survival outcome prediction using on‐treatment tumor kinetics and clinical parameters	Random survival forest	Exhibit superior predictive capability with a concordance index of 0.63	[367]

The clinical utility of AI algorithms in monitoring cancer progression has been explored. For example, PCMM‐Net was a DL framework that integrated prior clinic and radiological features of accurate lymphovascular invasion (LVI) prediction in breast cancer [357]. In a study involving 341 breast cancer patients, this approach improved the accuracy of LVI prediction. Nonrandomized clinical trials showed the efficiency of DL‐aided workflows for detecting breast cancer metastases by analyzing digital pathological images [358] and contrast‐enhanced MRI [359]. Radiomics‐based AI tools could accurately predict the malignant risk of ovarian tumors [363], and identify bone metastases in lung cancer [360] and prostate cancer [361]. AI trained using thyroid ultrasound (US) offered precise and reproducible predictions of cervical lymph node metastasis in PTC patients, potentially serving as a valuable assisting tool to improve the diagnostic performance of US radiologists [362]. Moreover, AI‐based models have also been applied to evaluate the efficacy of anticancer treatments and predict the prognosis of cancer patients. A machine learning‐based predictive model that integrated clinical features, dose‐volume histograms, radiomics, and dosiomics features was developed to predict radiation‐induced hypothyroidism (RIHT) in NPC patients undergoing tomotherapy [364]. As a result, the combined model showed superior performance in identifying potential RIHT patients and could aid in implementing preventative measures. An integrated model that combined CT‐based DL scores, blood‐based tumor mutational burden, and clinical parameters had the potential to predict tumor response to neoadjuvant chemoimmunotherapy in NSCLC patients, thus helping modify therapeutic regimens for patients [365]. CNN algorithms demonstrated greater prognostic potential for predicting overall survival in HCC patients compared with conventional radiomics approaches [366]. A machine learning model leveraging tumor kinetics demonstrated superior performance in predicting overall survival for patients with head and neck squamous cell carcinoma [367]. AI‐based systems have the potential to improve the evaluation of cancer treatment effectiveness.

Collectively, these trials not only verify the accuracy of AI‐assisted diagnosis but also demonstrate its impact on patient management, including treatment plan selection, prognosis assessment, and follow‐up strategies. Compared with conventional diagnostic methods, AI provides superior efficiency, reduced misdiagnosis rates, and improved treatment strategies. Nevertheless, despite the considerable potential of AI in cancer management, clinical trials still face many challenges, including data privacy concerns, algorithm interpretability, and clinician trust barriers in AI‐assisted decision‐making. Therefore, further study should strike a balance between advancing AI clinical applications and safeguarding patient data.

### 5.3 Real‐World Evidence: Effectiveness Validation in EHRs/Registry Databases

Real‐world evidence (RWE) studies mainly focus on gathering and analyzing observational data, providing insights into cancer treatment outcomes in routine practice. RWE resources include EHRs and patient registries. EHRs compile a wide array of real clinical data from patients, covering medical histories and treatment workflows. AI techniques, such as NLP and machine learning, have been applied to identify and retrieve important information from unstructured EHR data for scalable and efficient generation of RWE [368]. Based on unstructured EHR data, NLP‐based oncological frameworks identified treatment‐associated adverse events [369], optimized patient clinical management [370, 371], predicted treatment outcomes [372, 373, 374], and classified cancer recurrence status in clinical practice [375]. Machine learning models evaluated optimal treatment regimens and classified postoperative status in patients with lung cancer by analyzing real‐world patient data [376, 377]. Patient registry databases collected from various institutions become a value resource for generating RWE. Machine learning‐based models, including Bayesian and XGBoost, accurately predicted the effects of different treatments on patient survival outcomes [378], identified typical treatment sequences [379], and assessed the effects of pre‐existing chronic conditions on cancer‐directed treatments based on cancer registry databases [380]. RWE provides a robust foundation for advancing cancer care and guiding population‐level decision making. High‐quality clinical data are critical for generating credible RWE. AI techniques have the ability to handle large‐scale, heterogeneous real‐world data. To ensure generalizability and avoid overfitting, cross‐database validation and longitudinal assessments are urgently needed [381]. With increasing use of RWE in clinical decision‐making, cross‐sector collaboration is essential to establish data quality standards, address ethical issues, and ensure the long‐term efficiency of AI models.

### 5.4 Health Economic Evaluation: Cost Effectiveness of AI in Reducing Misdiagnosis/Diagnostic Delays

The employment of AI technology not only improves the efficiency of clinical decision‐making but may also significantly impact health economic evaluations [382]. AI systems can provide more accurate diagnoses and personalized treatment plans by analyzing a large amount of medical data, thereby reducing misdiagnosis rates and treatment costs. By detecting cancer rapidly, AI can facilitate early‐stage treatment for patients, thereby improving survival rates and reducing the economic burden associated with more advanced treatments [383]. However, the widespread application of AI in cancer care also faces some economic challenges. The development and implementation of AI technology necessitate substantial investment, including hardware facilities, software development, and the training of relevant personnel [384]. These initial investments may put pressure on the financial situation of medical institutions, especially in resource‐limited areas. The effectiveness and safety of AI systems need to be verified through strict clinical trials to ensure their reliability in actual applications, and this process requires a great deal of time and money.

In addition, the widespread adoption of AI in healthcare could result in the redistribution of medical resources and affect the economic model of traditional medical services. Medical insurance policies should be adjusted to adapt to the new challenges and opportunities presented by AI technology. To ensure the sustainability and effectiveness of AI technology in cancer management, it is necessary to comprehensively evaluate its cost effectiveness, long‐term economic impact, and its ability to improve patient health outcomes.

## 6 Clinical Implementation: Roadblocks and Pathways

### 6.1 Technical Limitations

The application of AI in clinical oncology faces two major technical limitations: data scarcity and algorithmic bias [385]. The data scarcity issue is primarily manifested by the insufficiency of high‐quality labeled data. The diversity and complexity of cancer pose significant challenges in obtaining sufficient clinical data, especially for rare cancer types [386]. The insufficient sample size not only hampers the training efficacy of AI models but may also lead to inadequate generalization in practical applications, thereby compromising the accuracy of cancer diagnosis and the effectiveness of cancer management. Algorithmic bias occurs when AI models are affected by inherent biases present in the training data during the learning process [387]. These biases can originate from various sources, including data collection methods, sample selection bias, or inadequate feature selection. For instance, if the training data mainly originate from a specific population, the performance of AI algorithms may significantly deteriorate when applied to other populations [388]. Moreover, the design and selection of the AI algorithm itself may also introduce bias, causing an over‐reliance on or neglect of certain features, which in turn affects the final decision‐making outcome [389]. Hence, addressing the issues of data scarcity and algorithmic bias is the key to enhancing the effectiveness of AI in cancer diagnosis and management. Future research should concentrate on building more comprehensive and diverse datasets, along with developing more robust algorithms to minimize the impact of bias on model performance. Through these endeavors, the full potential of AI technology can be better realized.

### 6.2 Clinical Integration Barriers

The gradual integration of AI technology into clinical cancer management faces numerous obstacles. Among these, workflow integration and physicians’ acceptance being particularly crucial [390]. The successful implementation of AI systems in healthcare requires their smooth integration into current medical workflows. Many hospitals and clinics have established multidepartmental workflows that involve collaboration and information sharing. Introducing AI technology may need significant adjustments to existing workflows. This involves not only technical integration but also considerations such as personal training and system compatibility. If effective integration is not achieved, it will be challenging to fully harness the potential of AI, and it may even lead to a decline in medical efficiency. Physicians’ acceptance of AI technology directly affects its clinical application [27]. While AI excels in data analysis and decision‐support, many physicians remain skeptical about its reliability and accuracy, especially regarding diagnostic accuracy and the potential to overlook nuanced patient symptoms. Some physicians are concerned that the growing dependence on AI could undermine their professional judgment, resulting in a reduced sense of professional security as their role in patient care evolves [35]. Some, on the other hand, see AI as a valuable tool that can augment human capabilities and lead to better patient outcomes. Education and training programs could help address these concerns by emphasizing the complementary roles of AI and human physicians. Additionally, physicians’ limited familiarity with AI technology hinders its practical application [391]. To overcome this, it is essential to enhance their awareness, build trust, and provide comprehensive training, thereby boosting their confidence in AI. In summary, addressing the challenges of workflow integration and gaining physicians’ acceptance are crucial steps in AI‐driven oncology. Through collaborative efforts from all stakeholders, we can ensure that AI technology fulfills its intended role in clinical practice.

### 6.3 Ethical and Regulatory Quandaries

In the era of rapid AI technology advancement, cancer diagnosis and management are experiencing unprecedented opportunities, yet a series of ethical and regulatory issues have also been triggered. First, the transparency and interpretability of AI platforms present significant challenges [392]. Many DL models are considered “black boxes” due to their complex internal mechanisms [393]. These models operate as an ambiguous system where their internal workings are not easily accessible or interpretable. They make predictions according to input data, but the systems cannot offer any suitable explanations involved behind their decision‐making processes. The lack of transparency may lead to decreased trust from physicians and patients in AI‐generated recommendations. Therefore, improving the interpretability of AI models is crucial for physicians to comprehend and validate their diagnostic outcomes, addressing an urgent need in the field. XAI has emerged as a solution to enhance the transparency of AI models. XAI can provide clinicians with clear insights into how AI models arrive at their predictions by clarifying AI decision‐making processes [394]. This bridges the gap between complex, opaque algorithms, and human comprehension, thus fostering greater trust in AI‐assisted clinical decisions. The widespread application of XAI faces multiple challenges, with expanding XAI technology and adapting to everchanging regulations being key obstacles to overcome. Generative pretrained transformers (GPTs) can also be used to demystify black‐box AI models. By training GPTs on extensive explanations of diverse problems and models, they can explain the fundamental algorithms, techniques, inputs and outputs of AI models, as well as how they are employed in decision making [395]. These explanations can shed light on the inner workings of black‐box models and provide insight into how they make predictions. The quality and accuracy of the training data, as well as the complexity and feature of black‐box models, may affect the precision of GPT‐generated explanations [396]. Combining GPT‐produced explanations with other techniques, including model‐specific interpretability techniques and model‐agnostic approaches, may further improve the interpretation of black‐box AI models. Second, ensuring data privacy and security is a critical ethical consideration in the application of AI for cancer patients [397]. Patients’ medical data often contain sensitive information, and maintaining their privacy during data collection and usage is a substantial challenge. With its clinical application in big data analysis, AI is expected to transform the future of oncology from diagnosis to treatment. However, it is necessary to address the ethical concerns regarding patient data usage. Furthermore, issues of data diversity and representativeness must be settled [398]. Biases in training data can lead to poor performance of AI in certain populations, thereby exacerbating health inequalities [399]. Last, the issues of liability attribution is equally intricate [400]. In the context of AI‐assisted diagnosis, determining liability in cases of misdiagnosis or missed diagnosis is a complex issue [401]. It is imperative to clarify who should be held accountable. Whether it is the company that develops the AI system, the medical institution that utilizes the system, or the physicians themselves. Real‐time feedback systems should be implemented to offer clinicians positive or negative feedback on AI‐assisted diagnostic decisions. This may enable clinicians to quickly make new and accurate diagnoses. Although AI holds great promise for cancer care, it is critically important to address its regulatory and ethical challenges thoroughly. This ensures the safe, effective, and fair application of AI technology in clinical oncology, thereby protecting patients’ rights and interests within the legal and ethical framework.

## 7 Future Frontiers: Next‐Generation AI Oncology

### 7.1 Technological Breakthroughs

In the realm of clinical oncology, causal inference and XAI have emerged as prominent research areas [402, 403]. Unlike correlation‐based approaches, causal inference aims to uncover direct relationships between variables, which facilitates the understanding of cancer initiation and progression [404]. By developing causal models, researchers can identify potential biomarkers and therapeutic targets, thereby paving the way for personalized medicine. For example, causal inference methods can be used to evaluate the effects of different treatment strategies on patient outcomes, assisting physicians in devising more effective treatment plans. Concurrently, XAI provides clinicians with more transparent decision‐support tools [405]. XAI technologies provide visualizations and explanations of the model's internal workings, thereby boosting physicians’ trust in AI systems [406]. This is particularly important in the context of early cancer diagnosis and treatment planning, where swift and accurate decisions are imperative in complex clinical settings. Nevertheless, implementing causal inference and XAI comes with its own set of challenges. Causal inference heavily depends on large amounts of high‐quality data, which can be difficult to acquire and integrate in cancer research [407]. Moreover, while XAI improves model transparency, striking a balance between model complexity and interpretability remains a pressing concern [408]. Therefore, these obstacles must be overcome to enable wider AI application in cancer care.

### 7.2 Clinical Translation Accelerators

For AI technology, establishing a global cooperation framework is of great significance [409]. With the rapid advancement of AI technology, international collaboration in cancer research and management has become increasingly necessary. By sharing data, technology, and best practices, international cooperation can accelerate the application of AI in cancer, improving diagnostic accuracy and treatment effectiveness [410]. First, establishing transnational research networks can facilitate data integration and sharing [409]. Cancer patients in different regions exhibit significant differences in genetic backgrounds, environmental factors, and lifestyles. Global data sharing contributes to training AI algorithms, enabling them to have stronger generalization abilities [411]. Moreover, international cooperation can facilitate multicenter clinical trials to validate the applicability and efficacy of AI technology across diverse populations. Second, the global cooperation framework plays a pivotal role in fostering the standardization and regularization of AI technologies in cancer management [410]. Currently, there exists a spectrum of differences among nations regarding the application standards, ethical norms, and regulatory policies for AI technology. These disparities can lead to inconsistencies in the safety and efficacy of AI applications across borders, which may hinder the seamless integration and adoption of these technologies in healthcare systems worldwide [412]. Through coordinated efforts by international organizations, it is possible to formulate unified standards that ensure the safe and effectiveness of AI technology. Such standardized approaches not only enhance public trust in AI but also provide researchers with clear guidance, facilitating rapid development and wide acceptance of innovative solutions. Moreover, establishing standardized ethical norms is essential for addressing concerns related to data privacy, algorithmic bias, and the responsible use of AI in healthcare [389]. By promoting consensus on these issues, international cooperation can build a robust framework that safeguards both patients and researchers. Therefore, harmonizing AI standards through global collaboration is essential for advancing clinical cancer care. Finally, global cooperation can facilitate the widespread adoption of education and training in AI for cancer management [413]. Organizing international conferences, offering online courses, and establishing exchange programs facilitate knowledge sharing and learning among researchers and clinicians from various countries [414]. This exchange enhances their understanding and application of AI technology, which in turn strengthens the foundation for advancements in global cancer management.

### 7.3 Policy and Equitable Implementation

Ensuring the equitable implementation of AI technology in oncology practice is of utmost importance [415]. To achieve this, policymakers must prioritize the accessibility of AI technology, especially in areas with limited resources. By investing in essential infrastructure and offering technical support, it can be ensured that all patients, regardless of their economic status, can benefit from AI‐driven medical services [416]. Moreover, governments and medical institutions need to enhance their oversight of AI technology to guarantee its transparency and fairness in clinical settings [417]. This is crucial to prevent diagnostic disparities arising from algorithmic biases. Education and training are also key to achieving equitable implementation [418]. Medical professionals must be trained in AI technology to effectively collaborate with these tools, understand their limitations, and recognize potential risks. Simultaneously, public education plays a vital role in raising awareness about the role of AI in cancer management and fostering patients’ trust in these new technologies [419]. Furthermore, policy recommendations should encompass financial support for research and development in AI technology, with a particular focus on ethnic minorities and low‐income groups [420]. This will facilitate the development of AI algorithms that are diverse and representative, thereby improving their applicability across different populations. By comprehensively considering the aforementioned factors, policymakers can promote the equitable implementation of AI technology in cancer management, ultimately leading to higher‐quality medical services and better patient outcomes.

## 8 Conclusions

AI has the potential to revolutionize cancer care by enhancing the accuracy of early detection, optimizing personalized treatment plans, and improving patient outcomes. However, the clinical application of AI still faces several challenges. The quality and diversity of data are pivotal factors affecting AI model performance. Insufficient high‐quality labeled data can lead to inadequate generalization of AI models. The transparency and interpretability of AI systems need urgent attention. Understanding the decision‐making process of AI by physicians and patients directly affects its clinical application. Moreover, ethical and privacy issues cannot be ignored, and achieving a balance between data utilization and patient privacy protection is crucial for future research. Collectively, further efforts should concentrate on refining algorithms, strengthening data‐sharing mechanisms, and establishing appropriate ethical frameworks to promote the deep integration of AI technology into clinical oncology.

## Author Contributions

Man Wang: conceptualization, supervision, investigation, writing—original draft preparation, and funding acquisition. Wenguang Chang: investigation and visualization. Yuan Zhang: writing—reviewing and editing. All authors have read and approved the final manuscript.

## Conflicts of Interest

The authors declare no conflicts of interest.

## Ethics Statement

The authors have nothing to report.

## Data Availability

Data sharing not applicable to this article as no datasets were generated or analyzed during the current study.

## References

[1] F. Bray , M. Laversanne , H. Sung , et al., “Global Cancer Statistics 2022: GLOBOCAN Estimates of Incidence and Mortality Worldwide for 36 Cancers in 185 Countries,” CA: A Cancer Journal for Clinicians 74, no. 3 (2024): 229–263. 38572751 10.3322/caac.21834

[2] A. E. Yuzhalin , “Redefining Cancer Research for Therapeutic Breakthroughs,” British Journal of Cancer 130, no. 7 (2024): 1078–1082. 38424166 10.1038/s41416-024-02634-6 PMC10991368

[3] R. C. Fitzgerald , A. C. Antoniou , L. Fruk , and N. Rosenfeld , “The Future of Early Cancer Detection,” Nature Medicine 28, no. 4 (2022): 666–677. 10.1038/s41591-022-01746-x 35440720

[4] M. Arruebo , N. Vilaboa , B. Saez‐Gutierrez , et al., “Assessment of the Evolution of Cancer Treatment Therapies,” Cancers (Basel) 3, no. 3 (2011): 3279–3330. 24212956 10.3390/cancers3033279 PMC3759197

[5] Y. Q. Liu , X. L. Wang , D. H. He , and Y. X. Cheng , “Protection Against Chemotherapy‐ and Radiotherapy‐induced Side Effects: A Review Based on the Mechanisms and Therapeutic Opportunities of Phytochemicals,” Phytomedicine 80 (2021): 153402. 33203590 10.1016/j.phymed.2020.153402

[6] A. H. Gunn , C. Sorenson , and R. A. Greenup , “Navigating the High Costs of Cancer Care: Opportunities for Patient Engagement,” Future Oncology 17, no. 28 (2021): 3729–3742. 34296620 10.2217/fon-2021-0341

[7] K. Shahid , M. Khalife , R. Dabney , and A. T. Phan , “Immunotherapy and Targeted Therapy‐the New Roadmap in Cancer Treatment,” Annals of Translational Medicine 7, no. 20 (2019): 595. 31807576 10.21037/atm.2019.05.58 PMC6861781

[8] L. Zhu , M. Jiang , H. Wang , et al., “A Narrative Review of Tumor Heterogeneity and Challenges to Tumor Drug Therapy,” Annals of Translational Medicine 9, no. 16 (2021): 1351. 34532488 10.21037/atm-21-1948 PMC8422119

[9] Y. Zhang and Z. Zhang , “The History and Advances in Cancer Immunotherapy: Understanding the Characteristics of Tumor‐infiltrating Immune Cells and Their Therapeutic Implications,” Cellular & Molecular Immunology 17, no. 8 (2020): 807–821. 32612154 10.1038/s41423-020-0488-6 PMC7395159

[10] S. Hussain , I. Mubeen , N. Ullah , et al., “Modern Diagnostic Imaging Technique Applications and Risk Factors in the Medical Field: A Review,” BioMed Research International 2022 (2022): 5164970. 35707373 10.1155/2022/5164970 PMC9192206

[11] S. Das , M. K. Dey , R. Devireddy , and M. R. Gartia , “Biomarkers in Cancer Detection, Diagnosis, and Prognosis,” Sensors (Basel) 24, no. 1 (2023): 37. 38202898 10.3390/s24010037 PMC10780704

[12] A. M. Sebastian and D. Peter , “Artificial Intelligence in Cancer Research: Trends, Challenges and Future Directions,” Life (Basel) 12, no. 12 (2022): 1991. 36556356 10.3390/life12121991 PMC9786074

[13] J. Guo , J. Hu , Y. Zheng , S. Zhao , and J. Ma , “Artificial Intelligence: Opportunities and Challenges in the Clinical Applications of Triple‐negative Breast Cancer,” British Journal of Cancer 128, no. 12 (2023): 2141–2149. 36871044 10.1038/s41416-023-02215-z PMC10241896

[14] V. Bellini , M. Cascella , F. Cutugno , et al., “Understanding Basic Principles of Artificial Intelligence: A Practical Guide for Intensivists,” Acta Biomedica 93, no. 5 (2022): e2022297. 36300214 10.23750/abm.v93i5.13626 PMC9686179

[15] Q. Pei , Y. Luo , Y. Chen , J. Li , D. Xie , and T. Ye , “Artificial Intelligence in Clinical Applications for Lung Cancer: Diagnosis, Treatment and Prognosis,” Clinical Chemistry and Laboratory Medicine 60, no. 12 (2022): 1974–1983. 35771735 10.1515/cclm-2022-0291

[16] C. D. Naylor , “On the Prospects for a (deep) Learning Health Care System,” Jama 320, no. 11 (2018): 1099–1100. 30178068 10.1001/jama.2018.11103

[17] F. Deng , J. Huang , X. Yuan , C. Cheng , and L. Zhang , “Performance and Efficiency of Machine Learning Algorithms for Analyzing Rectangular Biomedical Data,” Laboratory Investigation 101, no. 4 (2021): 430–441. 33574440 10.1038/s41374-020-00525-x

[18] Y. LeCun , Y. Bengio , and G. Hinton , “Deep Learning,” Nature 521, no. 7553 (2015): 436–444. 26017442 10.1038/nature14539

[19] B. Bhinder , C. Gilvary , N. S. Madhukar , and O. Elemento , “Artificial Intelligence in Cancer Research and Precision Medicine,” Cancer Discovery 11, no. 4 (2021): 900–915. 33811123 10.1158/2159-8290.CD-21-0090 PMC8034385

[20] H. Silva , G. N. M. Santos , A. F. Leite , et al., “The Use of Artificial Intelligence Tools in Cancer Detection Compared to the Traditional Diagnostic Imaging Methods: An Overview of the Systematic Reviews,” PLoS ONE 18, no. 10 (2023): e0292063. 37796946 10.1371/journal.pone.0292063 PMC10553229

[21] A. Aamir , A. Iqbal , F. Jawed , et al., “Exploring the Current and Prospective Role of Artificial Intelligence in Disease Diagnosis,” Annals of Medicine and Surgery (London) 86, no. 2 (2024): 943–949. 10.1097/MS9.0000000000001700 PMC10849462 38333305

[22] S. Askin , D. Burkhalter , G. Calado , and S. El Dakrouni , “Artificial Intelligence Applied to Clinical Trials: Opportunities and Challenges,” Health and Technology (Berlin) 13, no. 2 (2023): 203–213. 10.1007/s12553-023-00738-2 PMC9974218 36923325

[23] S. Maleki Varnosfaderani and M. Forouzanfar , “The Role of AI in Hospitals and Clinics: Transforming Healthcare in the 21st Century,” Bioengineering (Basel) 11, no. 4 (2024): 337. 38671759 10.3390/bioengineering11040337 PMC11047988

[24] D. Dixon , H. Sattar , N. Moros , et al., “Unveiling the Influence of AI Predictive Analytics on Patient Outcomes: A Comprehensive Narrative Review,” Cureus 16, no. 5 (2024): e59954. 38854327 10.7759/cureus.59954 PMC11161909

[25] A. Dubey and A. Tiwari , “Artificial Intelligence and Remote Patient Monitoring in US Healthcare Market: A Literature Review,” Journal of Market Access & Health Policy 11, no. 1 (2023): 2205618. 37151736 10.1080/20016689.2023.2205618 PMC10158563

[26] E. Sezgin , “Artificial Intelligence in Healthcare: Complementing, Not Replacing, Doctors and Healthcare Providers,” Digital Health 9 (2023): 20552076231186520. 37426593 10.1177/20552076231186520 PMC10328041

[27] D. Henzler , S. Schmidt , A. Kocar , et al., “Healthcare Professionals' perspectives on Artificial Intelligence in Patient Care: A Systematic Review of Hindering and Facilitating Factors on Different Levels,” BMC Health Services Research [Electronic Resource] 25, no. 1 (2025): 633. 40312413 10.1186/s12913-025-12664-2 PMC12046968

[28] Y. Balagurunathan , R. Mitchell , and I. El Naqa , “Requirements and Reliability of AI in the Medical Context,” Physical Medicine 83 (2021): 72–78. 10.1016/j.ejmp.2021.02.024 PMC8915137 33721700

[29] K. Palaniappan , E. Y. T. Lin , and S. Vogel , “Global Regulatory Frameworks for the Use of Artificial Intelligence (AI) in the,” Healthcare Services Sector Healthcare (Basel) 12, no. 5 (2024): 562. 38470673 10.3390/healthcare12050562 PMC10930608

[30] D. D. Farhud and S. Zokaei , “Ethical Issues of Artificial Intelligence in Medicine and Healthcare,” Iranian Journal of Public Health 50, no. 11 (2021): i–v. 10.18502/ijph.v50i11.7600 PMC8826344 35223619

[31] N. Bienefeld , E. Keller , and G. Grote , “AI Interventions to Alleviate Healthcare Shortages and Enhance Work Conditions in Critical Care: Qualitative Analysis,” Journal of Medical Internet Research [Electronic Resource] 27 (2025): e50852. 39805110 10.2196/50852 PMC11773285

[32] H. Shamszare and A. Choudhury , “Clinicians' Perceptions of Artificial Intelligence: Focus on Workload, Risk, Trust, Clinical Decision Making, and Clinical Integration,” Healthcare (Basel) 11, no. 16 (2023): 2308. 37628506 10.3390/healthcare11162308 PMC10454426

[33] T. Lysaght , H. Y. Lim , V. Xafis , and K. Y. Ngiam , “AI‐Assisted Decision‐making in Healthcare: The Application of an Ethics Framework for Big Data in Health and Research,” Asian Bioethics Review 11, no. 3 (2019): 299–314. 33717318 10.1007/s41649-019-00096-0 PMC7747260

[34] P. Esmaeilzadeh , “Challenges and Strategies for Wide‐scale Artificial Intelligence (AI) Deployment in Healthcare Practices: A Perspective for Healthcare Organizations,” Artificial Intelligence in Medicine 151 (2024): 102861. 38555850 10.1016/j.artmed.2024.102861

[35] T. P. Quinn , M. Senadeera , S. Jacobs , S. Coghlan , and V. Le , “Trust and Medical AI: The Challenges We Face and the Expertise Needed to Overcome Them,” Journal of the American Medical Informatics Association 28, no. 4 (2021): 890–894. 33340404 10.1093/jamia/ocaa268 PMC7973477

[36] S. Huang , J. Yang , N. Shen , Q. Xu , and Q. Zhao , “Artificial Intelligence in Lung Cancer Diagnosis and Prognosis: Current Application and Future Perspective,” Seminars in Cancer Biology 89 (2023): 30–37. 36682439 10.1016/j.semcancer.2023.01.006

[37] J. Calderaro , L. Zigutyte , D. Truhn , A. Jaffe , and J. N. Kather , “Artificial Intelligence in Liver Cancer—new Tools for Research and Patient Management,” Nature Reviews Gastroenterology & Hepatology 21, no. 8 (2024): 585–599. 38627537 10.1038/s41575-024-00919-y

[38] A. D. Haue , J. X. Hjaltelin , P. C. Holm , D. Placido , and S. R. Brunak , “Artificial Intelligence‐aided Data Mining of Medical Records for Cancer Detection and Screening,” The Lancet Oncology 25, no. 12 (2024): e694–e703. 39637906 10.1016/S1470-2045(24)00277-8

[39] M. Krassowski , V. Das , S. K. Sahu , and B. B. Misra , “State of the Field in Multi‐Omics Research: From Computational Needs to Data Mining and Sharing,” Frontiers in Genetics 11 (2020): 610798. 33362867 10.3389/fgene.2020.610798 PMC7758509

[40] Y. M. Chen , T. H. Hsiao , C. H. Lin , and Y. C. Fann , “Unlocking Precision Medicine: Clinical Applications of Integrating Health Records, Genetics, and Immunology Through Artificial Intelligence,” Journal of Biomedical Science 32, no. 1 (2025): 16. 39915780 10.1186/s12929-024-01110-w PMC11804102

[41] B. Van Calster , L. Wynants , D. Timmerman , E. W. Steyerberg , and G. S. Collins , “Predictive Analytics in Health Care: How Can We Know It Works?,” Journal of the American Medical Informatics Association 26, no. 12 (2019): 1651–1654. 31373357 10.1093/jamia/ocz130 PMC6857503

[42] N. Akhoon , “Precision Medicine: A New Paradigm in Therapeutics,” International Journal of Preventive Medicine 12 (2021): 12. 34084309 10.4103/ijpvm.IJPVM_375_19 PMC8106271

[43] N. Afzal , S. Sohn , S. Abram , et al., “Mining Peripheral Arterial Disease Cases From Narrative Clinical Notes Using Natural Language Processing,” Journal of Vascular Surgery 65, no. 6 (2017): 1753–1761. 28189359 10.1016/j.jvs.2016.11.031 PMC5438905

[44] J. Gazquez‐Garcia , C. L. Sanchez‐Bocanegra , and J. L. Sevillano , “AI in the Health Sector: Systematic Review of Key Skills for Future Health Professionals,” JMIR Medical Education 11 (2025): e58161. 39912237 10.2196/58161 PMC11822726

[45] S. Badillo , B. Banfai , F. Birzele , et al., “An Introduction to Machine Learning,” Clinical Pharmacology & Therapeutics 107, no. 4 (2020): 871–885. 32128792 10.1002/cpt.1796 PMC7189875

[46] H. Habehh and S. Gohel , “Machine Learning in Healthcare,” Current Genomics 22, no. 4 (2021): 291–300. 35273459 10.2174/1389202922666210705124359 PMC8822225

[47] N. Murali , A. Kucukkaya , A. Petukhova , J. Onofrey , and J. Chapiro , “Supervised Machine Learning in Oncology: A Clinician's Guide,” Digestive Disease Interventions 4, no. 1 (2020): 73–81. 32869010 10.1055/s-0040-1705097 PMC7456427

[48] T. Jiang , J. L. Gradus , and A. J. Rosellini , “Supervised Machine Learning: A Brief Primer,” Behavior Therapy 51, no. 5 (2020): 675–687. 32800297 10.1016/j.beth.2020.05.002 PMC7431677

[49] E. Christodoulou , J. Ma , G. S. Collins , E. W. Steyerberg , J. Y. Verbakel , and B. Van Calster , “A Systematic Review Shows no Performance Benefit of Machine Learning Over Logistic Regression for Clinical Prediction Models,” Journal of Clinical Epidemiology 110 (2019): 12–22. 30763612 10.1016/j.jclinepi.2019.02.004

[50] M. Kim , J. Yun , Y. Cho , et al., “Deep Learning in Medical Imaging,” Neurospine 16, no. 4 (2019): 657–668. 31905454 10.14245/ns.1938396.198 PMC6945006

[51] V. Rani , S. T. Nabi , M. Kumar , A. Mittal , and K. Kumar , “Self‐supervised Learning: A Succinct Review,” Archives of Computational Methods in Engineering 30, no. 4 (2023): 2761–2775. 36713767 10.1007/s11831-023-09884-2 PMC9857922

[52] Y. Xie , Z. Xu , J. Zhang , Z. Wang , and S. Ji , “Self‐Supervised Learning of Graph Neural Networks: A Unified Review,” Ieee Transactions on Pattern Analysis and Machine Intelligence 45, no. 2 (2023): 2412–2429. 35476575 10.1109/TPAMI.2022.3170559 PMC9902037

[53] S. Albelwi , “Survey on Self‐Supervised Learning: Auxiliary Pretext Tasks and Contrastive Learning Methods in Imaging,” Entropy (Basel) 24, no. 4 (2022): 551. 35455214 10.3390/e24040551 PMC9029566

[54] F. Navarro , C. Watanabe , S. Shit , et al., “Self‐Supervised Pretext Tasks in Model Robustness & Generalizability: A Revisit From Medical Imaging Perspective,” Annual International Conference of the IEEE Engineering in Medicine and Biology Society 2022 (2022): 5074–5079. 36086344 10.1109/EMBC48229.2022.9870911

[55] H. Pan , Y. Guo , Q. Deng , H. Yang , J. Chen , and Y. Chen , “Improving Fine‐tuning of Self‐supervised Models With Contrastive Initialization,” Neural Networks 159 (2023): 198–207. 36584625 10.1016/j.neunet.2022.12.012

[56] S. J. Wagner , D. Reisenbuchler , N. P. West , et al., “Transformer‐based Biomarker Prediction From Colorectal Cancer Histology: A Large‐scale Multicentric Study,” Cancer Cell 41, no. 9 (2023): 1650–1661. e4. 37652006 10.1016/j.ccell.2023.08.002 PMC10507381

[57] A. Claudio Quiros , N. Coudray , A. Yeaton , et al., “Mapping the Landscape of Histomorphological Cancer Phenotypes Using Self‐supervised Learning on Unannotated Pathology Slides,” Nature Communications 15, no. 1 (2024): 4596. 10.1038/s41467-024-48666-7 PMC11525555 38862472

[58] N. Akalin and A. Loutfi , “Reinforcement Learning Approaches in Social Robotics,” Sensors (Basel) 21, no. 4 (2021): 1292. 33670257 10.3390/s21041292 PMC7918897

[59] R. Nian , J. Liu , and B. Huang , “A Review on Reinforcement Learning: Introduction and Applications in Industrial Process Control,” Computers & Chemical Engineering 139 (2020): 106886.

[60] M. N. A. Al‐Hamadani , M. A. Fadhel , L. Alzubaidi , and H. Balazs , “Reinforcement Learning Algorithms and Applications in Healthcare and Robotics: A Comprehensive and Systematic Review,” Sensors (Basel) 24, no. 8 (2024): 2461. 38676080 10.3390/s24082461 PMC11053800

[61] H. Elmekki , S. Islam , A. Alagha , et al., “Comprehensive Review of Reinforcement Learning for Medical Ultrasound Imaging,” Artificial Intelligence Review 58, no. 9 (2025): 284. 40567264 10.1007/s10462-025-11268-w PMC12185671

[62] G. Molinaro and A. G. E. Collins , “Intrinsic Rewards Explain Context‐sensitive Valuation in Reinforcement Learning,” Plos Biology 21, no. 7 (2023): e3002201. 37459394 10.1371/journal.pbio.3002201 PMC10374061

[63] T. C. Frommeyer , M. M. Gilbert , R. M. Fursmidt , et al., “Reinforcement Learning and Its Clinical Applications within Healthcare: A Systematic Review of Precision Medicine and Dynamic Treatment Regimes,” Healthcare (Basel) 13, no. 14 (2025): 1752. 40724777 10.3390/healthcare13141752 PMC12295150

[64] T. Qamar and N. Z. Bawany , “Understanding the Black‐box: Towards Interpretable and Reliable Deep Learning Models,” PeerJ Computer Science 9 (2023): e1629. 10.7717/peerj-cs.1629 PMC10702969 38077598

[65] L. Alzubaidi , J. Zhang , A. J. Humaidi , et al., “Review of Deep Learning: Concepts, CNN Architectures, Challenges, Applications, Future Directions,” Journal of Big Data 8, no. 1 (2021): 53. 33816053 10.1186/s40537-021-00444-8 PMC8010506

[66] J. M. Vaz and S. Balaji , “Convolutional Neural Networks (CNNs): Concepts and Applications in Pharmacogenomics,” Molecular Diversity 25, no. 3 (2021): 1569–1584. 34031788 10.1007/s11030-021-10225-3 PMC8342355

[67] T. Ersavas , M. A. Smith , and J. S. Mattick , “Novel Applications of Convolutional Neural Networks in the Age of Transformers,” Scientific Reports 14, no. 1 (2024): 10000. 38693215 10.1038/s41598-024-60709-z PMC11063149

[68] A. B , M. Kaur , D. Singh , S. Roy , and M. Amoon , “Efficient Skip Connections‐Based Residual Network (ESRNet) for Brain Tumor Classification,” Diagnostics (Basel) 13, no. 20 (2023): 3234. 37892055 10.3390/diagnostics13203234 PMC10606037

[69] Z. Zhou , J. Shin , L. Zhang , S. Gurudu , M. Gotway , and J. Liang , “Fine‐tuning Convolutional Neural Networks for Biomedical Image Analysis: Actively and Incrementally,” Proceedings of the IEEE Conference on Computer Vision and Pattern Recognition 2017 (2017): 4761–4772. 30337799 10.1109/CVPR.2017.506 PMC6191179

[70] J. Noorbakhsh , S. Farahmand , A. Foroughi Pour , et al., “Deep Learning‐based Cross‐classifications Reveal Conserved Spatial Behaviors Within Tumor Histological Images,” Nature Communications 11, no. 1 (2020): 6367. 10.1038/s41467-020-20030-5 PMC7733499 33311458

[71] S. Nerella , S. Bandyopadhyay , J. Zhang , et al., “Transformers and Large Language Models in Healthcare: A Review,” Artificial Intelligence in Medicine 154 (2024): 102900. 38878555 10.1016/j.artmed.2024.102900 PMC11638972

[72] S. R. Choi and M. Lee , “Transformer Architecture and Attention Mechanisms in Genome Data Analysis: A Comprehensive Review,” Biology (Basel) 12, no. 7 (2023): 1033. 37508462 10.3390/biology12071033 PMC10376273

[73] G. Tucudean , M. Bucos , B. Dragulescu , and C. D. Caleanu , “Natural Language Processing With Transformers: A Review,” PeerJ Computer Science 10 (2024): e2222. 10.7717/peerj-cs.2222 PMC11322986 39145251

[74] X. Lin , L. Yu , K. T. Cheng , and Z. Yan , “The Lighter the Better: Rethinking Transformers in Medical Image Segmentation through Adaptive Pruning,” IEEE Transactions on Medical Imaging 42, no. 8 (2023): 2325–2337. 37027664 10.1109/TMI.2023.3247814

[75] R. Wang , V. Bashyam , Z. Yang , et al., “Applications of Generative Adversarial Networks in Neuroimaging and Clinical Neuroscience,” Neuroimage 269 (2023): 119898. 36702211 10.1016/j.neuroimage.2023.119898 PMC9992336

[76] M. H. Akpinar , A. Sengur , M. Salvi , et al., “Synthetic Data Generation via Generative Adversarial Networks in Healthcare: A Systematic Review of Image‐ and Signal‐Based Studies,” IEEE Open Journal of Engineering in Medicine and Biology 6 (2025): 183–192. 39698120 10.1109/OJEMB.2024.3508472 PMC11655107

[77] S. P. Porkodi , V. Sarada , V. Maik , and K. Gurushankar , “Generic Image Application Using GANs (Generative Adversarial Networks): A Review,” Evolving Systems (Berlin) (2022): 1–15. 10.1007/s12530-022-09464-y PMC9523650 40479410

[78] Y. J. Yeo , Y. G. Shin , S. Park , and S. J. Ko , “Simple yet Effective Way for Improving the Performance of GAN,” IEEE Transactions on Neural Networks and Learning Systems 33, no. 4 (2022): 1811–1818. 33385312 10.1109/TNNLS.2020.3045000

[79] J. J. Jeong , A. Tariq , T. Adejumo , H. Trivedi , J. W. Gichoya , and I. Banerjee , “Systematic Review of Generative Adversarial Networks (GANs) for Medical Image Classification and Segmentation,” Journal of Digital Imaging 35, no. 2 (2022): 137–152. 35022924 10.1007/s10278-021-00556-w PMC8921387

[80] Y. Zhang and J. Hong , “Challenges of Deep Learning in Cancers,” Technology in Cancer Research & Treatment 22 (2023): 15330338231173495. 37113071 10.1177/15330338231173495 PMC10150420

[81] S. Pati , S. Kumar , A. Varma , et al., “Privacy Preservation for Federated Learning in Health Care,” Patterns (N Y) 5, no. 7 (2024): 100974. 39081567 10.1016/j.patter.2024.100974 PMC11284498

[82] B. Yurdem , M. Kuzlu , M. K. Gullu , F. O. Catak , and M. Tabassum , “Federated Learning: Overview, Strategies, Applications, Tools and Future Directions,” Heliyon 10, no. 19 (2024): e38137. 39391509 10.1016/j.heliyon.2024.e38137 PMC11466570

[83] L. Che , J. Wang , Y. Zhou , and F. Ma , “Multimodal Federated Learning: A Survey,” Sensors (Basel) 23, no. 15 (2023): 6986. 37571768 10.3390/s23156986 PMC10422520

[84] Y. Shi , J. Zhang , M. Xue , et al., “Vertical Federated Learning Based on Data Subset Representation for Healthcare Application,” Computer Methods and Programs in Biomedicine 263 (2025): 108623. 39954511 10.1016/j.cmpb.2025.108623

[85] I. Kholod , E. Yanaki , D. Fomichev , et al., “Open‐Source Federated Learning Frameworks for IoT: A Comparative Review and Analysis,” Sensors (Basel) 21, no. 1 (2020): 167. 33383803 10.3390/s21010167 PMC7794892

[86] A. Tahir , F. Chen , H. U. Khan , Z. Ming , A. Ahmad , and S. Nazir , “A Systematic Review on Cloud Storage Mechanisms Concerning e‐Healthcare Systems,” Sensors (Basel) 20, no. 18 (2020): 5392. 32967094 10.3390/s20185392 PMC7570508

[87] E. Alalwany and I. Mahgoub , “Security and Trust Management in the Internet of Vehicles (IoV): Challenges and Machine Learning Solutions,” Sensors (Basel) 24, no. 2 (2024): 368. 38257461 10.3390/s24020368 PMC10819911

[88] A. Bourechak , O. Zedadra , M. N. Kouahla , A. Guerrieri , H. Seridi , and G. Fortino , “At the Confluence of Artificial Intelligence and Edge Computing in IoT‐Based Applications: A Review and New Perspectives,” Sensors (Basel) 23, no. 3 (2023): 1639. 36772680 10.3390/s23031639 PMC9920982

[89] R. Vano , I. Lacalle , P. Sowinski , R. S‐Julián , and C. E. Palau , “Cloud‐Native Workload Orchestration at the Edge: A Deployment Review and Future Directions,” Sensors (Basel) 23, no. 4 (2023): 2215. 36850813 10.3390/s23042215 PMC9967903

[90] M. Babar , M. Ahmad Jan , X. He , M. Usman Tariq , S. Mastorakis , and R. Alturki , “An Optimized IoT‐enabled Big Data Analytics Architecture for Edge‐Cloud Computing,” IEEE Internet of Things Journal 10, no. 5 (2023): 3995–4005. 38046398 10.1109/jiot.2022.3157552 PMC10691823

[91] S. Hamdan , M. Ayyash , and S. Almajali , “Edge‐Computing Architectures for Internet of Things Applications: A Survey,” Sensors (Basel) 20, no. 22 (2020): 6441. 33187267 10.3390/s20226441 PMC7696529

[92] A. Garcia‐Perez , R. Minon , A. I. Torre‐Bastida , and E. Zulueta‐Guerrero , “Analysing Edge Computing Devices for the Deployment of Embedded AI,” Sensors (Basel) 23, no. 23 (2023): 9495. 38067868 10.3390/s23239495 PMC10708856

[93] O. Ali , M. K. Ishak , M. K. L. Bhatti , I. Khan , and K. I. Kim , “A Comprehensive Review of Internet of Things: Technology Stack, Middlewares, and Fog/Edge Computing Interface,” Sensors (Basel) 22, no. 3 (2022): 995. 35161740 10.3390/s22030995 PMC8840251

[94] N. A. Angel , D. Ravindran , P. Vincent , K. Srinivasan , and Y. C. Hu , “Recent Advances in Evolving Computing Paradigms: Cloud, Edge, and Fog Technologies,” Sensors (Basel) 22, no. 1 (2021): 196. 35009740 10.3390/s22010196 PMC8749780

[95] D. C. Klonoff , “Fog Computing and Edge Computing Architectures for Processing Data from Diabetes Devices Connected to the Medical Internet of Things,” Journal of Diabetes Science and Technology 11, no. 4 (2017): 647–652. 28745086 10.1177/1932296817717007 PMC5588847

[96] G. Carvalho , B. Cabral , V. Pereira , and J. Bernardino , “Edge Computing: Current Trends, Research Challenges and Future Directions,” Computing 103, no. 5 (2021): 993–1023.

[97] L. Belcastro , J. Carretero , and D. Talia , “Edge‐Cloud Solutions for Big Data Analysis and Distributed Machine Learning ‐ 1,” Future Generation Computer Systems 159 (2024): 323–326.

[98] A. M. Alwakeel , “An Overview of Fog Computing and Edge Computing Security and Privacy Issues,” Sensors (Basel) 21, no. 24 (2021): 8226. 34960320 10.3390/s21248226 PMC8708798

[99] Y. Mansouri and M. A. Babar , “A Review of Edge Computing: Features and Resource Virtualization,” Journal of Parallel and Distributed Computing 150 (2021): 155–183.

[100] S. Dash , S. K. Shakyawar , M. Sharma , and S. Kaushik , “Big Data in Healthcare: Management, Analysis and Future Prospects,” Journal of Big Data 6, no. 1 (2019): 54.

[101] Y. Gao , D. Cao , M. Li , et al., “Integration of Multiomics Features for Blood‐based Early Detection of Colorectal Cancer,” Molecular Cancer 23, no. 1 (2024): 173. 39175001 10.1186/s12943-024-01959-3 PMC11340186

[102] T. Liu , J. Huang , T. Liao , R. Pu , S. Liu , and Y. Peng , “A Hybrid Deep Learning Model for Predicting Molecular Subtypes of Human Breast Cancer Using Multimodal Data,” IRBM 43, no. 1 (2022): 62–74.

[103] O. Lapuente‐Santana , G. Sturm , J. Kant , et al., “Multimodal Analysis Unveils Tumor Microenvironment Heterogeneity Linked to Immune Activity and Evasion,” Iscience 27, no. 8 (2024): 110529. 39161957 10.1016/j.isci.2024.110529 PMC11331718

[104] Z. Liu and S. Zhang , “Tumor Characterization and Stratification by Integrated Molecular Profiles Reveals Essential Pan‐cancer Features,” BMC Genomics [Electronic Resource] 16, no. 1 (2015): 503. 26148869 10.1186/s12864-015-1687-x PMC4491878

[105] W. G. Breen , M. P. Aryal , Y. Cao , and M. M. Kim , “Integrating Multi‐modal Imaging in Radiation Treatments for Glioblastoma,” Neuro‐Oncology 26, no. 12 Suppl 2 (2024): S17–S25. 38437666 10.1093/neuonc/noad187 PMC10911793

[106] R. S. Vanguri , J. Luo , A. T. Aukerman , et al., “Multimodal Integration of Radiology, Pathology and Genomics for Prediction of Response to PD‐(L)1 Blockade in Patients With Non‐small Cell Lung Cancer,” Nature Cancer 3, no. 10 (2022): 1151–1164. 36038778 10.1038/s43018-022-00416-8 PMC9586871

[107] K. Tan , W. Huang , X. Liu , J. Hu , and S. Dong , “A Multi‐modal Fusion Framework Based on Multi‐task Correlation Learning for Cancer Prognosis Prediction,” Artificial Intelligence in Medicine 126 (2022): 102260. 35346442 10.1016/j.artmed.2022.102260

[108] Y. Hao , C. Cheng , J. Li , et al., “Multimodal Integration in Health Care: Development with Applications in Disease Management,” Journal of Medical Internet Research [Electronic Resource] 27 (2025): e76557. 40840463 10.2196/76557 PMC12370271

[109] S. Khalighi , K. Reddy , A. Midya , K. B. Pandav , A. Madabhushi , and M. Abedalthagafi , “Artificial Intelligence in Neuro‐oncology: Advances and Challenges in Brain Tumor Diagnosis, Prognosis, and Precision Treatment,” NPJ Precision Oncology 8, no. 1 (2024): 80. 38553633 10.1038/s41698-024-00575-0 PMC10980741

[110] S. N. Histed , M. L. Lindenberg , E. Mena , B. Turkbey , P. L. Choyke , and K. A. Kurdziel , “Review of Functional/Anatomical Imaging in Oncology,” Nuclear Medicine Communications 33, no. 4 (2012): 349–361. 22314804 10.1097/MNM.0b013e32834ec8a5 PMC3295905

[111] C. Zhang , J. Xu , R. Tang , et al., “Novel Research and Future Prospects of Artificial Intelligence in Cancer Diagnosis and Treatment,” Journal of Hematology & Oncology 16, no. 1 (2023): 114. 38012673 10.1186/s13045-023-01514-5 PMC10680201

[112] S. Yelne , M. Chaudhary , K. Dod , A. Sayyad , and R. Sharma , “Harnessing the Power of AI: A Comprehensive Review of Its Impact and Challenges in Nursing Science and Healthcare,” Cureus 15, no. 11 (2023): e49252. 38143615 10.7759/cureus.49252 PMC10744168

[113] Y. Kumar , A. Koul , R. Singla , and M. F. Ijaz , “Artificial Intelligence in Disease Diagnosis: A Systematic Literature Review, Synthesizing Framework and Future Research Agenda,” Journal of Ambient Intelligence and Humanized Computing 14, no. 7 (2023): 8459–8486. 35039756 10.1007/s12652-021-03612-z PMC8754556

[114] R. Paudyal , A. D. Shah , O. Akin , et al., “Artificial Intelligence in CT and MR Imaging for Oncological Applications,” Cancers (Basel) 15, no. 9 (2023): 2573. 37174039 10.3390/cancers15092573 PMC10177423

[115] S. Vedantham , H. W. Tseng , Z. Fu , and H. S. Chow , “Dedicated Cone‐Beam Breast CT: Reproducibility of Volumetric Glandular Fraction With Advanced Image Reconstruction Methods,” Tomography 9, no. 6 (2023): 2039–2051. 37987346 10.3390/tomography9060160 PMC10661286

[116] S. P. Jakkaladiki and F. Maly , “Integrating Hybrid Transfer Learning With Attention‐enhanced Deep Learning Models to Improve Breast Cancer Diagnosis,” PeerJ Computer Science 10 (2024): e1850. 10.7717/peerj-cs.1850 PMC10909230 38435578

[117] S. Endo , K. Nagata , K. Utano , et al., “Development and Validation of Computer‐aided Detection for Colorectal Neoplasms Using Deep Learning Incorporated With Computed Tomography Colonography,” BMC Gastroenterology [Electronic Resource] 25, no. 1 (2025): 149. 40055612 10.1186/s12876-025-03742-0 PMC11889859

[118] M. Takeuchi , T. Seto , M. Hashimoto , et al., “Performance of a Deep Learning‐based Identification System for Esophageal Cancer From CT Images,” Esophagus 18, no. 3 (2021): 612–620. 33635412 10.1007/s10388-021-00826-0

[119] C. Dong , T. Z. Li , K. Xu , et al., “Characterizing Browser‐based Medical Imaging AI With Serverless Edge Computing: Towards Addressing Clinical Data Security Constraints,” Proceedings of Spie the International Society for Optical Engineering 12469 (2023): 1246907. 37063644 10.1117/12.2653626 PMC10099365

[120] R. Zahari , J. Cox , and B. Obara , “Uncertainty‐aware Image Classification on 3D CT Lung,” Computers in Biology and Medicine 172 (2024): 108324. 38508053 10.1016/j.compbiomed.2024.108324

[121] J. V. Sousa , P. Matos , F. Silva , P. Freitas , H. P. Oliveira , and T. Pereira , “Single Modality vs. Multimodality: What Works Best for Lung Cancer Screening?,” Sensors (Basel) 23, no. 12 (2023): 5597. 37420765 10.3390/s23125597 PMC10301640

[122] A. Saha , S. M. Ganie , P. K. D. Pramanik , R. K. Yadav , S. Mallik , and Z. Zhao , “VER‐Net: A Hybrid Transfer Learning Model for Lung Cancer Detection Using CT Scan Images,” BMC Medical Imaging 24, no. 1 (2024): 120. 38789925 10.1186/s12880-024-01238-z PMC11127393

[123] M. Naghavi , I. De Oliveira , S. S. Mao , et al., “Opportunistic AI‐enabled Automated Bone Mineral Density Measurements in Lung Cancer Screening and Coronary Calcium Scoring CT Scans Are Equivalent,” European Journal of Radiology Open 10 (2023): 100492. 37214544 10.1016/j.ejro.2023.100492 PMC10196960

[124] S. Karimullah , M. Khan , F. Shaik , B. Alabduallah , and A. Almjally , “An Integrated Method for Detecting Lung Cancer via CT Scanning via Optimization, Deep Learning, and IoT Data Transmission,” Frontiers in Oncology 14 (2024): 1435041. 39435294 10.3389/fonc.2024.1435041 PMC11491319

[125] U. Batra , S. Nathany , S. K. Nath , et al., “AI‐based Pipeline for Early Screening of Lung Cancer: Integrating Radiology, Clinical, and Genomics Data,” Lancet Regional Health – Southeast Asia 24 (2024): 100352. 38756151 10.1016/j.lansea.2024.100352 PMC11096686

[126] S. Aslani , P. Alluri , E. Gudmundsson , et al., “Enhancing Cancer Prediction in Challenging Screen‐detected Incident Lung Nodules Using Time‐series Deep Learning,” Computerized Medical Imaging and Graphics 116 (2024): 102399. 38833895 10.1016/j.compmedimag.2024.102399

[127] R. Yang , Y. Zhang , W. Li , et al., “Development and External Validation of a Multimodal Integrated Feature Neural Network (MIFNN) for the Diagnosis of Malignancy in Small Pulmonary Nodules (</= 10 mm),” Biomedical Physics & Engineering Express 10, no. 4 (2024): 5008. 10.1088/2057-1976/ad449a 38684143

[128] S. Yang , S. H. Lim , J. H. Hong , J. S. Park , J. Kim , and H. W. Kim , “Deep Learning‐based Lung Cancer Risk Assessment Using Chest Computed Tomography Images Without Pulmonary Nodules >/= 8 Mm,” Translational Lung Cancer Research 14, no. 1 (2025): 150–162. 39958220 10.21037/tlcr-24-882 PMC11826273

[129] Y. Jiang and V. S. K. Manem , “Data Augmented Lung Cancer Prediction Framework Using the Nested Case Control NLST Cohort,” Frontiers in Oncology 15 (2025): 1492758. 40071099 10.3389/fonc.2025.1492758 PMC11893409

[130] J. Simon , P. Mikhael , A. Graur , et al., “Significance of Image Reconstruction Parameters for Future Lung Cancer Risk Prediction Using Low‐Dose Chest Computed Tomography and the Open‐Access Sybil Algorithm,” Investigative Radiology 60, no. 5 (2025): 311–318. 39437009 10.1097/RLI.0000000000001131 PMC12129392

[131] Y. Wang , A. Gupta , F. I. Tushar , et al., “Concordance‐based Predictive Uncertainty (CPU)‐Index: Proof‐of‐concept With Application towards Improved Specificity of Lung Cancers on Low Dose Screening CT,” Artificial Intelligence in Medicine 160 (2025): 103055. 39721356 10.1016/j.artmed.2024.103055 PMC12139530

[132] L. Meng , P. Zhu , and K. Xia , “Application Value of the Automated Machine Learning Model Based on Modified CT Index Combined With Serological Indices in the Early Prediction of Lung Cancer,” Frontiers in Public Health 12 (2024): 1368217. 38645446 10.3389/fpubh.2024.1368217 PMC11027066

[133] Y. Wang , C. Zhou , L. Ying , et al., “Enhancing Early Lung Cancer Diagnosis: Predicting Lung Nodule Progression in Follow‐Up Low‐Dose CT Scan With Deep Generative Model,” Cancers (Basel) 16, no. 12 (2024): 2229. 38927934 10.3390/cancers16122229 PMC11201561

[134] I. Bhatia , A. S. I. Aarti , F. Amin , and A. Alabrah , “Lightweight Advanced Deep Neural Network (DNN) Model for Early‐Stage Lung Cancer Detection,” Diagnostics (Basel) 14, no. 21 (2024): 2356. 39518324 10.3390/diagnostics14212356 PMC11545829

[135] I. Bhatia , Aarti , S. I. Ansarullah , F. Amin , and A. Alabrah , “An Advanced Lung Carcinoma Prediction and Risk Screening Model Using Transfer Learning,” Diagnostics (Basel) 14, no. 13 (2024): 1378. 39001268 10.3390/diagnostics14131378 PMC11241604

[136] R. R. Shivwanshi and N. Nirala , “Hyperparameter Optimization and Development of an Advanced CNN‐based Technique for Lung Nodule Assessment,” Physics in Medicine and Biology 68, no. 17 (2023): 175038. 10.1088/1361-6560/acef8c 37567211

[137] M. Wang , Z. Yang , and R. Zhao , “Advancing Lung Cancer Diagnosis: Combining 3D Auto‐encoders and Attention Mechanisms for CT Scan Analysis,” Journal of X‐Ray Science and Technology 33, no. 2 (2025): 376–392. 39973792 10.1177/08953996241313120

[138] H. H. Chang , C. Z. Wu , and A. H. Gallogly , “Pulmonary Nodule Classification Using a Multiview Residual Selective Kernel Network,” Journal of Imaging Informatics in Medicine 37, no. 1 (2024): 347–362. 38343233 10.1007/s10278-023-00928-4 PMC10976931

[139] Y. Hussain Ali , V. Sabu Chooralil , K. Balasubramanian , et al., “Optimization System Based on Convolutional Neural Network and Internet of Medical Things for Early Diagnosis of Lung Cancer,” Bioengineering (Basel) 10, no. 3 (2023): 320. 36978711 10.3390/bioengineering10030320 PMC10045046

[140] L. Ma , C. Wan , K. Hao , A. Cai , and L. Liu , “A Novel Fusion Algorithm for Benign‐malignant Lung Nodule Classification on CT Images,” BMC Pulmonary Medicine 23, no. 1 (2023): 474. 38012620 10.1186/s12890-023-02708-w PMC10683224

[141] J. R. Tugwell‐Allsup , B. W. Owen , R. Hibbs , and A. England , “The Use of Artificial Intelligence to Aid the Diagnosis of Lung Cancer—A Retrospective‐cohort Study,” Radiography (London) 31, no. 2 (2025): 102876. 10.1016/j.radi.2025.01.011 39890480

[142] R. Hu , Y. Xie , L. Zhang , et al., “A Two‐stage Deep‐learning Framework for CT Denoising Based on a Clinically Structure‐unaligned Paired Data Set,” Quantitative Imaging in Medicine and Surgery 14, no. 1 (2024): 335–351. 38223072 10.21037/qims-23-403 PMC10784028

[143] S. Lam , M. W. Wynes , C. Connolly , et al., “The International Association for the Study of Lung Cancer Early Lung Imaging Confederation Open‐Source Deep Learning and Quantitative Measurement Initiative,” Journal of Thoracic Oncology 19, no. 1 (2024): 94–105. 37595684 10.1016/j.jtho.2023.08.016

[144] F. I. Tushar , L. Vancoillie , C. McCabe , et al., “Virtual Lung Screening Trial (VLST): An in Silico Study Inspired by the National Lung Screening Trial for Lung Cancer Detection,” Medical Image Analysis 103 (2025): 103576. 40209556 10.1016/j.media.2025.103576 PMC12147717

[145] Z. Gao , Z. Yu , X. Zhang , et al., “Development of a Deep Learning Model for Early Gastric Cancer Diagnosis Using Preoperative Computed Tomography Images,” Frontiers in Oncology 13 (2023): 1265366. 37869090 10.3389/fonc.2023.1265366 PMC10587601

[146] C. Peng , P. L. H. Yu , J. Lu , et al., “Opportunistic Detection of Hepatocellular Carcinoma Using Noncontrast CT and Deep Learning Artificial Intelligence,” Journal of the American College of Radiology 22, no. 3 (2025): 249–259. 40044303 10.1016/j.jacr.2024.12.011

[147] E. Sahin , O. C. Tatar , M. E. Ulutas , et al., “Diagnostic Performance of Deep Learning Applications in Hepatocellular Carcinoma Detection Using Computed Tomography Imaging,” The Turkish Journal of Gastroenterology: The Official Journal of Turkish Society of Gastroenterology 36, no. 2 (2024): 124–130. 39760649 10.5152/tjg.2024.24538 PMC11851832

[148] L. Li , T. Liu , P. Wang , et al., “Multiple Perception Contrastive Learning for Automated Ovarian Tumor Classification in CT Images,” Abdominal Radiology (NY) 50, no. 9 (2025): 4342–4358. 10.1007/s00261-025-04879-y 40074925

[149] S. Amiri , R. Karimzadeh , T. Vrtovec , et al., “Centerline‐guided Reinforcement Learning Model for Pancreatic Duct Identifications,” Journal of Medical Imaging (Bellingham) 11, no. 6 (2024): 064002. 10.1117/1.JMI.11.6.064002 PMC11543826 39525832

[150] Y. Shi , H. Tang , M. J. Baine , et al., “3DGAUnet: 3D Generative Adversarial Networks With a 3D U‐Net Based Generator to Achieve the Accurate and Effective Synthesis of Clinical Tumor Image Data for Pancreatic Cancer,” Cancers (Basel) 15, no. 23 (2023): 5496. 38067200 10.3390/cancers15235496 PMC10705188

[151] S. Mandal , K. Balraj , H. Kodamana , et al., “Weakly Supervised Large‐scale Pancreatic Cancer Detection Using Multi‐instance Learning,” Frontiers in Oncology 14 (2024): 1362850. 39267824 10.3389/fonc.2024.1362850 PMC11390448

[152] C. Abi Nader , R. Vetil , L. K. Wood , et al., “Automatic Detection of Pancreatic Lesions and Main Pancreatic Duct Dilatation on Portal Venous CT Scans Using Deep Learning,” Investigative Radiology 58, no. 11 (2023): 791–798. 37289274 10.1097/RLI.0000000000000992

[153] M. Ramaekers , C. G. A. Viviers , T. A. E. Hellstrom , et al., “Improved Pancreatic Cancer Detection and Localization on CT Scans: A Computer‐Aided Detection Model Utilizing Secondary Features,” Cancers (Basel) 16, no. 13 (2024): 2403. 39001465 10.3390/cancers16132403 PMC11240790

[154] Y. Alaca , “Machine Learning via DARTS‐Optimized MobileViT Models for Pancreatic Cancer Diagnosis With Graph‐based Deep Learning,” BMC Medical Informatics and Decision Making [Electronic Resource] 25, no. 1 (2025): 81. 39955532 10.1186/s12911-025-02923-x PMC11830204

[155] A. Nadeem , R. Ashraf , T. Mahmood , and S. Parveen , “Automated CAD System for Early Detection and Classification of Pancreatic Cancer Using Deep Learning Model,” PLoS ONE 20, no. 1 (2025): e0307900. 39752442 10.1371/journal.pone.0307900 PMC11698441

[156] A. S. Minhas and R. Oliver , “Magnetic Resonance Imaging Basics,” Advances in Experimental Medicine and Biology 1380 (2022): 47–82. 36306094 10.1007/978-3-031-03873-0_3

[157] M. C. Florkow , K. Willemsen , V. V. Mascarenhas , E. H. G. Oei , M. van Stralen , and P. R. Seevinck , “Magnetic Resonance Imaging versus Computed Tomography for Three‐Dimensional Bone Imaging of Musculoskeletal Pathologies: A Review,” Journal of Magnetic Resonance Imaging 56, no. 1 (2022): 11–34. 35044717 10.1002/jmri.28067 PMC9305220

[158] J. N. C. Raimundo , J. P. P. Fontes , L. Gonzaga Mendes Magalhaes , and M. A. Guevara Lopez , “An Innovative Faster R‐CNN‐Based Framework for Breast Cancer Detection in MRI,” Journal of Imaging 9, no. 9 (2023): 169. 37754933 10.3390/jimaging9090169 PMC10532017

[159] C. Sahaya Pushpa Sarmila Star , T. M. Inbamalar , and A. Milton , “Segmentation of Breast Lesion Using Fuzzy Thresholding and Deep Learning,” Computers in Biology and Medicine 184 (2025): 109406. 39531925 10.1016/j.compbiomed.2024.109406

[160] J. Ouyang , K. T. Chen , R. Duarte Armindo , G. A. Davidzon , K. E. Hawk , and F. Moradi , “Predicting FDG‐PET Images from Multi‐Contrast MRI Using Deep Learning in Patients with Brain Neoplasms,” Journal of Magnetic Resonance Imaging 59, no. 3 (2024): 1010–1020. 37259967 10.1002/jmri.28837 PMC10689577

[161] M. Ariful Islam , M. F. Mridha , M. Safran , S. Alfarhood , and M. Mohsin Kabir , “Revolutionizing Brain Tumor Detection Using Explainable AI in MRI Images,” NMR in Biomedicine 38, no. 3 (2025): e70001. 39948696 10.1002/nbm.70001

[162] M. Cheng , H. Zhang , Y. Guo , et al., “Comparison of MRI and CT Based Deep Learning Radiomics Analyses and Their Combination for Diagnosing Intrahepatic Cholangiocarcinoma,” Scientific Reports 15, no. 1 (2025): 9629. 40113926 10.1038/s41598-025-92263-7 PMC11926170

[163] S. Ziegelmayer , A. W. Marka , M. Strenzke , et al., “Speed and Efficiency: Evaluating Pulmonary Nodule Detection With AI‐enhanced 3D Gradient Echo Imaging,” European Radiology 35, no. 4 (2025): 2237–2244. 39154315 10.1007/s00330-024-11027-5 PMC11914225

[164] Y. Yuan , E. Ahn , D. Feng , M. Khadra , and J. Kim , “Z‐SSMNet: Zonal‐aware Self‐supervised Mesh Network for Prostate Cancer Detection and Diagnosis With Bi‐parametric MRI,” Computerized Medical Imaging and Graphics 122 (2025): 102510. 40010011 10.1016/j.compmedimag.2025.102510

[165] T. Chen , W. Hu , Y. Zhang , et al., “A Multimodal Deep Learning Nomogram for the Identification of Clinically Significant Prostate Cancer in Patients With Gray‐Zone PSA Levels: Comparison With Clinical and Radiomics Models,” Academic Radiology 32, no. 2 (2025): 864–876. 39496535 10.1016/j.acra.2024.10.009

[166] H. Huang , J. Mo , Z. Ding , et al., “Deep Learning to Simulate Contrast‐Enhanced MRI for Evaluating Suspected Prostate Cancer,” Radiology 314, no. 1 (2025): e240238. 39807983 10.1148/radiol.240238

[167] Y. J. Lee , H. W. Moon , M. H. Choi , S. Eun Jung , Y. H. Park , and J. Y. Lee , “MRI‐based Deep Learning Algorithm for Assisting Clinically Significant Prostate Cancer Detection: A Bicenter Prospective Study,” Radiology 314, no. 3 (2025): e232788. 40067105 10.1148/radiol.232788

[168] L. M. Esteban , A. Borque‐Fernando , M. E. Escorihuela , J. Esteban‐Escano , J. M. Abascal , and P. Servian , “Integrating Radiological and Clinical Data for Clinically Significant Prostate Cancer Detection With Machine Learning Techniques,” Scientific Reports 15, no. 1 (2025): 4261. 39905119 10.1038/s41598-025-88297-6 PMC11794621

[169] M. Chondronikola and S. Sarkar , “Total‐body PET Imaging: A New Frontier for the Assessment of Metabolic Disease and Obesity,” PET Clinics 16, no. 1 (2021): 75–87. 33160928 10.1016/j.cpet.2020.09.001

[170] X. Li , B. Pan , C. Chen , et al., “Clinical Evaluation of Deep Learning‐enhanced Lymphoma Pet Imaging With Accelerated Acquisition,” Journal of Applied Clinical Medical Physics [Electronic Resource] 25, no. 9 (2024): e14390. 38812107 10.1002/acm2.14390 PMC11492391

[171] T. Yang , D. Liu , Z. Zhang , R. Sa , and F. Guan , “Predicting T‐Cell Lymphoma in Children from (18)F‐FDG PET‐CT Imaging with Multiple Machine Learning Models,” Journal of Imaging Informatics in Medicine 37, no. 3 (2024): 952–964. 38321311 10.1007/s10278-024-01007-y PMC11169166

[172] P. Guan , K. Yu , W. Wei , Y. Tan , and J. Wu , “Big Data Analytics on Lung Cancer Diagnosis Framework with Deep Learning,” IEEE Transactions on Computational Biology and Bioinformatics 21, no. 4 (2024): 757–768. 37256795 10.1109/TCBB.2023.3281638

[173] P. Shyamala Bharathi and C. Shalini , “Advanced Hybrid Attention‐based Deep Learning Network With Heuristic Algorithm for Adaptive CT and PET Image Fusion in Lung Cancer Detection,” Medical Engineering & Physics 126 (2024): 104138. 38621836 10.1016/j.medengphy.2024.104138

[174] C. Jiang , C. Qian , Q. Jiang , et al., “Virtual Biopsy for Non‐invasive Identification of Follicular Lymphoma Histologic Transformation Using Radiomics‐based Imaging Biomarker From PET/CT,” BMC Medicine [Electronic Resource] 23, no. 1 (2025): 49. 39875864 10.1186/s12916-025-03893-7 PMC11776338

[175] A. Soliman , Z. Li , and A. V. Parwani , “Artificial Intelligence's Impact on Breast Cancer Pathology: A Literature Review,” Diagnostic Pathology 19, no. 1 (2024): 38. 38388367 10.1186/s13000-024-01453-w PMC10882736

[176] K. Lami , H. S. Yoon , A. V. Parwani , et al., “Validation of Prostate and Breast Cancer Detection Artificial Intelligence Algorithms for Accurate Histopathological Diagnosis and Grading: A Retrospective Study With a Japanese Cohort,” Pathology 56, no. 5 (2024): 633–642. 38719771 10.1016/j.pathol.2024.02.009

[177] J. Sandbank , G. Bataillon , A. Nudelman , et al., “Validation and Real‐world Clinical Application of an Artificial Intelligence Algorithm for Breast Cancer Detection in Biopsies,” NPJ Breast Cancer 8, no. 1 (2022): 129. 36473870 10.1038/s41523-022-00496-w PMC9723672

[178] S. Ahuja and S. Zaheer , “Advancements in Pathology: Digital Transformation, Precision Medicine, and Beyond,” Journal of Pathology Informatics 16 (2025): 100408. 40094037 10.1016/j.jpi.2024.100408 PMC11910332

[179] B. Challa , M. Tahir , Y. Hu , et al., “Artificial Intelligence‐Aided Diagnosis of Breast Cancer Lymph Node Metastasis on Histologic Slides in a Digital Workflow,” Modern Pathology 36, no. 8 (2023): 100216. 37178923 10.1016/j.modpat.2023.100216

[180] C. Eloy , A. Marques , J. Pinto , et al., “Artificial Intelligence‐assisted Cancer Diagnosis Improves the Efficiency of Pathologists in Prostatic Biopsies,” Virchows Archiv 482, no. 3 (2023): 595–604. 36809483 10.1007/s00428-023-03518-5 PMC10033575

[181] R. Pannala , K. Krishnan , J. Melson , et al., “Artificial Intelligence in Gastrointestinal Endoscopy,” VideoGIE 5, no. 12 (2020): 598–613. 33319126 10.1016/j.vgie.2020.08.013 PMC7732722

[182] P. Sanga , J. Singh , A. K. Dubey , et al., “DermAI 1.0: A Robust, Generalized, and Novel Attention‐Enabled Ensemble‐Based Transfer Learning Paradigm for Multiclass Classification of Skin Lesion Images,” Diagnostics (Basel) 13, no. 19 (2023): 3159. 37835902 10.3390/diagnostics13193159 PMC10573070

[183] X. Matias‐Guiu , G. Stanta , F. Carneiro , et al., “The Leading Role of Pathology in Assessing the Somatic Molecular Alterations of Cancer: Position Paper of the European Society of Pathology,” Virchows Archiv 476, no. 4 (2020): 491–497. 32124002 10.1007/s00428-020-02757-0 PMC7156353

[184] I. Kim , K. Kang , Y. Song , and T. J. Kim , “Application of Artificial Intelligence in Pathology: Trends and Challenges,” Diagnostics (Basel) 12, no. 11 (2022): 2794. 36428854 10.3390/diagnostics12112794 PMC9688959

[185] Z. Ahmad , S. Rahim , M. Zubair , and J. Abdul‐Ghafar , “Artificial Intelligence (AI) in Medicine, Current Applications and Future Role With Special Emphasis on Its Potential and Promise in Pathology: Present and Future Impact, Obstacles Including Costs and Acceptance Among Pathologists, Practical and Philosophical Considerations. A Comprehensive Review,” Diagnostic Pathology 16, no. 1 (2021): 24. 33731170 10.1186/s13000-021-01085-4 PMC7971952

[186] B. Huang , F. Yang , M. Yin , X. Mo , and C. Zhong , “A Review of Multimodal Medical Image Fusion Techniques,” Computational and Mathematical Methods in Medicine 2020 (2020): 8279342. 32377226 10.1155/2020/8279342 PMC7195632

[187] Z. Liu , Y. Wu , H. Xu , et al., “Multimodal Fusion of Radio‐pathology and Proteogenomics Identify Integrated Glioma Subtypes With Prognostic and Therapeutic Opportunities,” Nature Communications 16, no. 1 (2025): 3510. 10.1038/s41467-025-58675-9 PMC11994800 40222975

[188] B. Lokaj , V. Durand de Gevigney , D. A. Djema , et al., “Multimodal Deep Learning Fusion of Ultrafast‐DCE MRI and Clinical Information for Breast Lesion Classification,” Computers in Biology and Medicine 188 (2025): 109721. 39978091 10.1016/j.compbiomed.2025.109721

[189] M. Li , Y. Fang , J. Shao , et al., “Vision Transformer‐based Multimodal Fusion Network for Classification of Tumor Malignancy on Breast Ultrasound: A Retrospective Multicenter Study,” International Journal of Medical Informatics 196 (2025): 105793. 39862564 10.1016/j.ijmedinf.2025.105793

[190] Y. Cho , S. Misra , R. Managuli , R. G. Barr , J. Lee , and C. Kim , “Attention‐based Fusion Network for Breast Cancer Segmentation and Classification Using Multi‐modal Ultrasound Images,” Ultrasound in Medicine & Biology 51, no. 3 (2025): 568–577. 39694743 10.1016/j.ultrasmedbio.2024.11.020

[191] S. Shirae , S. S. Debsarkar , H. Kawanaka , B. Aronow , and V. B. S. Prasath , “Multimodal Ensemble Fusion Deep Learning Using Histopathological Images and Clinical Data for Glioma Subtype Classification,” IEEE Access 13 (2025): 57780–57797. 40260100 10.1109/access.2025.3556713 PMC12011355

[192] H. Xu and R. Lv , “Rapid Diagnosis of Lung Cancer by Multi‐modal Spectral Data Combined With Deep Learning,” Spectrochimica Acta Part A, Molecular and Biomolecular Spectroscopy 335 (2025): 125997. 40073660 10.1016/j.saa.2025.125997

[193] S. J. H. Shah , A. Albishri , R. Wang , and Y. Lee , “Integrating Local and Global Attention Mechanisms for Enhanced Oral Cancer Detection and Explainability,” Computers in Biology and Medicine 189 (2025): 109841. 40056841 10.1016/j.compbiomed.2025.109841 PMC13094401

[194] Y. Wang , S. Chi , Y. Tian , et al., “Construction of an Artificially Intelligent Model for Accurate Detection of HCC by Integrating Clinical, Radiological, and Peripheral Immunological Features,” International Journal of Surgery 111, no. 4 (2025): 2942–2952. 39878177 10.1097/JS9.0000000000002281 PMC12175786

[195] S. Cabon , S. Brihi , R. Fezzani , M. Pierre‐Jean , M. Cuggia , and G. Bouzille , “Combining a Risk Factor Score Designed from Electronic Health Records with a Digital Cytology Image Scoring System to Improve Bladder Cancer Detection: Proof‐of‐Concept Study,” Journal of Medical Internet Research [Electronic Resource] 27 (2025): e56946. 39841985 10.2196/56946 PMC11799811

[196] H. Yang , M. Yang , J. Chen , G. Yao , Q. Zou , and L. Jia , “Multimodal Deep Learning Approaches for Precision Oncology: A Comprehensive Review,” Briefings in Bioinformatics 26, no. 1 (2024): bbae699. 39757116 10.1093/bib/bbae699 PMC11700660

[197] B. P. Cabral , L. A. M. Braga , S. Syed‐Abdul , and F. B. Mota , “Future of Artificial Intelligence Applications in Cancer Care: A Global Cross‐Sectional Survey of Researchers,” Current Oncology 30, no. 3 (2023): 3432–3446. 36975473 10.3390/curroncol30030260 PMC10047823

[198] D. Capper , D. T. W. Jones , M. Sill , et al., “DNA Methylation‐based Classification of central Nervous System Tumours,” Nature 555, no. 7697 (2018): 469–474. 29539639 10.1038/nature26000 PMC6093218

[199] S. Benfatto , M. Sill , D. T. W. Jones , et al., “Explainable Artificial Intelligence of DNA Methylation‐based Brain Tumor Diagnostics,” Nature Communications 16, no. 1 (2025): 1787. 10.1038/s41467-025-57078-0 PMC11842776 39979307

[200] J. Shen , L. Lu , Z. Chen , et al., “Multi‐omics Analysis Constructs a Novel Neuroendocrine Prostate Cancer Classifier and Classification System,” Scientific Reports 15, no. 1 (2025): 13901. 40263498 10.1038/s41598-025-96683-3 PMC12015331

[201] A. A. Alzahrani , J. Alsamri , M. Maashi , et al., “Deep Structured Learning With Vision Intelligence for Oral Carcinoma Lesion Segmentation and Classification Using Medical Imaging,” Scientific Reports 15, no. 1 (2025): 6610. 39994267 10.1038/s41598-025-89971-5 PMC11850820

[202] K. Lakshmi , S. Amaran , G. Subbulakshmi , S. Padmini , G. P. Joshi , and W. Cho , “Explainable Artificial Intelligence With UNet Based Segmentation and Bayesian Machine Learning for Classification of Brain Tumors Using MRI Images,” Scientific Reports 15, no. 1 (2025): 690. 39753735 10.1038/s41598-024-84692-7 PMC11699199

[203] E. Hassan and H. Ghadiri , “Advancing Brain Tumor Classification: A Robust Framework Using EfficientNetV2 Transfer Learning and Statistical Analysis,” Computers in Biology and Medicine 185 (2025): 109542. 39657446 10.1016/j.compbiomed.2024.109542

[204] V. Satushe , V. Vyas , S. Metkar , and D. Paul Singh , “Advanced CNN Architecture for Brain Tumor Segmentation and Classification Using BraTS‐GOAT 2024 Dataset,” Current Medical Imaging 21 (2025): e15734056344235. 39757669 10.2174/0115734056344235241217155930 PMC13096864

[205] S. Abirami , K. Ramesh , and K. Lalitha VaniSree , “Classification and Pixel Change Detection of Brain Tumor Using Adam Kookaburra Optimization‐Based Shepard Convolutional Neural Network,” Nmr in Biomedicine 38, no. 2 (2025): e5307. 39832921 10.1002/nbm.5307

[206] Y. Mao , J. Kim , L. Podina , and M. Kohandel , “Dilated SE‐DenseNet for Brain Tumor MRI Classification,” Scientific Reports 15, no. 1 (2025): 3596. 39875423 10.1038/s41598-025-86752-y PMC11775108

[207] S. Ganesh , R. Gomathi , and S. Kannadhasan , “Brain Tumor Segmentation and Detection in MRI Using Convolutional Neural Networks and VGG16,” Cancer Biomarkers 42, no. 3 (2025): 18758592241311184. 40183298 10.1177/18758592241311184 PMC12288401

[208] K. A. Huang , A. Alkadri , and N. Prakash , “Employing Squeeze‐and‐Excitation Architecture in a Fine‐Tuned Convolutional Neural Network for Magnetic Resonance Imaging Tumor Classification,” Cureus 17, no. 3 (2025): e80084. 40190925 10.7759/cureus.80084 PMC11970440

[209] M. A. Ilani , D. Shi , and Y. M. Banad , “T1‐weighted MRI‐based Brain Tumor Classification Using Hybrid Deep Learning Models,” Scientific Reports 15, no. 1 (2025): 7010. 40016334 10.1038/s41598-025-92020-w PMC11868382

[210] P. Jyothi and S. Dhanasekaran , “An Attention 3DUNET and Visual Geometry Group‐19 Based Deep Neural Network for Brain Tumor Segmentation and Classification From MRI,” Journal of Biomolecular Structure & Dynamics 43, no. 2 (2025): 730–741. 37979152 10.1080/07391102.2023.2283164

[211] K. Pugazharasi and K. Sakthivel , “Enhanced Glioma Tumor Detection and Segmentation Using Modified Deep Learning With Edge Fusion and Frequency Features,” Scientific Reports 15, no. 1 (2025): 6899. 40011472 10.1038/s41598-024-84661-0 PMC11865600

[212] M. Kaddes , Y. M. Ayid , A. M. Elshewey , and Y. Fouad , “Breast Cancer Classification Based on Hybrid CNN With LSTM Model,” Scientific Reports 15, no. 1 (2025): 4409. 39910136 10.1038/s41598-025-88459-6 PMC11799331

[213] G. G. Lakshmi and P. Nagaraj , “Lung Cancer Detection and Classification Using Optimized CNN Features and Squeeze‐Inception‐ResNeXt Model,” Computational Biology and Chemistry 117 (2025): 108437. 40158238 10.1016/j.compbiolchem.2025.108437

[214] R. Tanaka , Y. Tsuboshita , M. Okodo , et al., “Artificial Intelligence Recognition Model Using Liquid‐Based Cytology Images to Discriminate Malignancy and Histological Types of Non‐Small‐Cell Lung Cancer,” Pathobiology 92, no. 1 (2025): 52–62. 39197433 10.1159/000541148

[215] V. P. Gladis Pushparathi , D. Justin Xavier , P. Chitra , and G. Kannan , “Prostate Cancer Classification and Interpretation with Multiparametric Magnetic Resonance Imaging and Gleason Grade Score Using DarkNet53 Model,” Prostate 85, no. 3 (2025): 294–307. 39584618 10.1002/pros.24827

[216] J. H. Han , B. W. Kim , T. M. Kim , et al., “Fully Automated Segmentation and Classification of Renal Tumors on CT Scans via Machine Learning,” BMC Cancer 25, no. 1 (2025): 173. 39881216 10.1186/s12885-025-13582-6 PMC11781067

[217] H. Guo , J. Zhang , Y. Li , X. Pan , and C. Sun , “Advanced Pathological Subtype Classification of Thyroid Cancer Using efficientNetB0,” Diagnostic Pathology 20, no. 1 (2025): 28. 40055769 10.1186/s13000-025-01621-6 PMC11887243

[218] P. Bedi , S. Rani , B. Gupta , V. Bhasin , and P. Gole , “EpiBrCan‐Lite: A Lightweight Deep Learning Model for Breast Cancer Subtype Classification Using Epigenomic Data,” Computer Methods and Programs in Biomedicine 260 (2025): 108553. 39667144 10.1016/j.cmpb.2024.108553

[219] K. Borah , H. S. Das , R. K. Budhathoki , K. Aurangzeb , and S. Mallik , “DOMSCNet: A Deep Learning Model for the Classification of Stomach Cancer Using Multi‐layer Omics Data,” Briefings in Bioinformatics 26, no. 2 (2025): bbaf115. 40178281 10.1093/bib/bbaf115 PMC11966610

[220] L. Yin and J. Wang , “Enhancing Brain Tumor Classification by Integrating Radiomics and Deep Learning Features: A Comprehensive Study Utilizing Ensemble Methods on MRI Scans,” Journal of X‐Ray Science and Technology 33, no. 1 (2025): 47–57. 39973780 10.1177/08953996241299996

[221] D. Li , W. Hu , L. Ma , et al., “Deep Learning Radiomics Nomograms Predict Isocitrate Dehydrogenase (IDH) Genotypes in Brain Glioma: A Multicenter Study,” Magnetic Resonance Imaging 117 (2025): 110314. 39708927 10.1016/j.mri.2024.110314

[222] Y. Zeng , Y. Zhang , Z. Xiao , and H. Sui , “A Multi‐classification Deep Neural Network for Cancer Type Identification From High‐dimension, Small‐sample and Imbalanced Gene Microarray Data,” Scientific Reports 15, no. 1 (2025): 5239. 39939378 10.1038/s41598-025-89475-2 PMC11822135

[223] D. Kawahara , M. Kishi , Y. Kadooka , K. Hirose , and Y. Murakami , “Integrating Radiomics and Gene Expression by Mapping on the Image With Improved DeepInsight for Clear Cell Renal Cell Carcinoma,” Cancer Genetics 292‐293 (2025): 100–105. 10.1016/j.cancergen.2025.02.004 39983665

[224] B. Zhang , J. Zhu , R. Xu , et al., “A Combined Model Integrating Radiomics and Deep Learning Based on Multiparametric Magnetic Resonance Imaging for Classification of Brain Metastases,” Acta Radiologica 66, no. 1 (2025): 24–34. 39552295 10.1177/02841851241292528

[225] D. Bertsimas , G. A. Margonis , S. Sujichantararat , et al., “Interpretable Artificial Intelligence to Optimise Use of imatinib After Resection in Patients With Localised Gastrointestinal Stromal Tumours: An Observational Cohort Study,” The Lancet Oncology 25, no. 8 (2024): 1025–1037. 38976997 10.1016/S1470-2045(24)00259-6 PMC12051465

[226] Z. Wang , D. N. Chen , X. Y. Huang , et al., “Machine Learning‐based Autophagy‐related Prognostic Signature for Personalized Risk Stratification and Therapeutic Approaches in Bladder Cancer,” International Immunopharmacology 138 (2024): 112623. 38991630 10.1016/j.intimp.2024.112623

[227] D. Chatziisaak , P. Burri , M. Sparn , D. Hahnloser , T. Steffen , and S. Bischofberger , “Concordance of ChatGPT Artificial Intelligence Decision‐making in Colorectal Cancer Multidisciplinary Meetings: Retrospective Study,” BJS Open 9, no. 3 (2025): zraf040. 40331891 10.1093/bjsopen/zraf040 PMC12056934

[228] H. M. Kim , S. S. Byun , J. K. Kim , et al., “Machine Learning‐based Prediction Model for Late Recurrence After Surgery in Patients With Renal Cell Carcinoma,” BMC Medical Informatics and Decision Making [Electronic Resource] 22, no. 1 (2022): 241. 36100881 10.1186/s12911-022-01964-w PMC9472380

[229] W. H. Song and M. Park , “RCC‐Supporter: Supporting Renal Cell Carcinoma Treatment Decision‐making Using Machine Learning,” BMC Medical Informatics and Decision Making [Electronic Resource] 24, no. Suppl 2 (2024): 259. 39285449 10.1186/s12911-024-02660-7 PMC11403845

[230] R. M. Gomez Del Moral Herranz , M. J. Lopez Rodriguez , A. P. Seiffert , J. Soto Perez‐Olivares , M. Chiva De Agustin , and P. Sanchez‐Gonzalez , “CureMate: A Clinical Decision Support System for Breast Cancer Treatment,” International Journal of Medical Informatics 192 (2024): 105647. 39393123 10.1016/j.ijmedinf.2024.105647

[231] G. H. Choi , J. Yun , J. Choi , et al., “Development of Machine Learning‐based Clinical Decision Support System for Hepatocellular Carcinoma,” Scientific Reports 10, no. 1 (2020): 14855. 32908183 10.1038/s41598-020-71796-z PMC7481788

[232] X. Liang , R. Guan , J. Zhu , et al., “A Clinical Decision Support System to Predict the Efficacy for EGFR‐TKIs Based on Artificial Neural Network,” Journal of Cancer Research and Clinical Oncology 149, no. 13 (2023): 12265–12274. 37434091 10.1007/s00432-023-05104-3 PMC11797839

[233] G. Valdes , C. B. Simone 2nd , J. Chen , et al., “Clinical Decision Support of Radiotherapy Treatment Planning: A Data‐driven Machine Learning Strategy for Patient‐specific Dosimetric Decision Making,” Radiotherapy and Oncology 125, no. 3 (2017): 392–397. 29162279 10.1016/j.radonc.2017.10.014

[234] W. Li , Y. H. Huang , T. Zhu , et al., “Noninvasive Artificial Intelligence System for Early Predicting Residual Cancer Burden during Neoadjuvant Chemotherapy in Breast Cancer,” Annals of Surgery 281, no. 4 (2025): 645–654. 38557792 10.1097/SLA.0000000000006279 PMC11888841

[235] M. H. A. Janse , L. M. Janssen , B. H. M. van der Velden , et al., “Deep Learning‐Based Segmentation of Locally Advanced Breast Cancer on MRI in Relation to Residual Cancer Burden: A Multi‐Institutional Cohort Study,” Journal of Magnetic Resonance Imaging 58, no. 6 (2023): 1739–1749. 36928988 10.1002/jmri.28679

[236] A. J. Widman , M. Shah , A. Frydendahl , et al., “Ultrasensitive Plasma‐based Monitoring of Tumor Burden Using Machine‐learning‐guided Signal Enrichment,” Nature Medicine 30, no. 6 (2024): 1655–1666. 10.1038/s41591-024-03040-4 PMC7616143 38877116

[237] D. Grama , M. Dahele , B. Slotman , and W. Verbakel , “Explainable AI for Raising Confidence in Deep Learning‐based Tumor Tracking Models,” Medical Physics 52, no. 7 (2025): e17940. 40660895 10.1002/mp.17940 PMC12260770

[238] Y. H. Huang , Z. Y. Shi , T. Zhu , et al., “Longitudinal MRI‐Driven Multi‐Modality Approach for Predicting Pathological Complete Response and B Cell Infiltration in Breast Cancer,” Advanced Science (Weinheim) 12, no. 12 (2025): e2413702. 10.1002/advs.202413702 PMC11948082 39921294

[239] M. C. Comes , A. Fanizzi , S. Bove , et al., “Monitoring over Time of Pathological Complete Response to Neoadjuvant Chemotherapy in Breast Cancer Patients through an Ensemble Vision Transformers‐Based Model,” Cancer Medicine 13, no. 24 (2024): e70482. 39692281 10.1002/cam4.70482 PMC11653217

[240] M. Gilad , S. C. Partridge , M. Iima , R. R. Md , and M. Freiman , “Radiomics‐based Machine Learning Prediction of Neoadjuvant Chemotherapy Response in Breast Cancer Using Physiologically Decomposed Diffusion‐weighted MRI,” Radiology Imaging Cancer 7, no. 4 (2025): e240312. 40679371 10.1148/rycan.240312 PMC12304534

[241] Z. Zhang , T. Luo , M. Yan , et al., “Voxel‐level Radiomics and Deep Learning for Predicting Pathologic Complete Response in Esophageal Squamous Cell Carcinoma After Neoadjuvant Immunotherapy and Chemotherapy,” Journal for ImmunoTherapy of Cancer 13, no. 3 (2025): e011149. 40090670 10.1136/jitc-2024-011149 PMC11911808

[242] H. Shen , Z. Jin , Q. Chen , et al., “Image‐based Artificial Intelligence for the Prediction of Pathological Complete Response to Neoadjuvant Chemoradiotherapy in Patients With Rectal Cancer: A Systematic Review and Meta‐analysis,” Radiologia Medica 129, no. 4 (2024): 598–614. 38512622 10.1007/s11547-024-01796-w

[243] E. Domingo , S. Rathee , A. Blake , et al., “Identification and Validation of a Machine Learning Model of Complete Response to Radiation in Rectal Cancer Reveals Immune Infiltrate and TGFbeta as Key Predictors,” EBioMedicine 106 (2024): 105228. 39013324 10.1016/j.ebiom.2024.105228 PMC11663784

[244] Y. Wang , Z. Pan , S. Li , et al., “Prediction and Validation of Pathologic Complete Response for Locally Advanced Rectal Cancer Under Neoadjuvant Chemoradiotherapy Based on a Novel Predictor Using Interpretable Machine Learning,” European Journal of Surgical Oncology 50, no. 12 (2024): 108738. 39395242 10.1016/j.ejso.2024.108738

[245] X. Jin , X. Zheng , D. Chen , J. Jin , G. Zhu , and X. Deng , “Prediction of Response After Chemoradiation for Esophageal Cancer Using a Combination of Dosimetry and CT Radiomics,” European Radiology 29, no. 11 (2019): 6080–6088. 31028447 10.1007/s00330-019-06193-w

[246] Z. Cai , S. Li , Z. Xiong , J. Lin , and Y. Sun , “Multimodal MRI‐based Deep‐radiomics Model Predicts Response in Cervical Cancer Treated With Neoadjuvant Chemoradiotherapy,” Scientific Reports 14, no. 1 (2024): 19090. 39154103 10.1038/s41598-024-70055-9 PMC11330439

[247] G. Macchia , S. Cilla , D. Pezzulla , et al., “Efficacy of Stereotactic Body Radiotherapy and Response Prediction Using Artificial Intelligence in Oligometastatic Gynaecologic Cancer,” Gynecologic Oncology 184 (2024): 16–23. 38271773 10.1016/j.ygyno.2024.01.023

[248] A. Kedves , M. Akay , Y. Akay , et al., “Predictive Value of Magnetic Resonance Imaging Diffusion Parameters Using Artificial Intelligence in Low‐and Intermediate‐risk Prostate Cancer Patients Treated With Stereotactic Ablative Radiotherapy: A Pilot Study,” Radiography (London) 30, no. 3 (2024): 986–994. 10.1016/j.radi.2024.03.015 38678978

[249] P. Johannet , N. Coudray , D. M. Donnelly , G. Jour , I. Illa‐Bochaca , and Y. Xia , “Using Machine Learning Algorithms to Predict Immunotherapy Response in Patients With Advanced Melanoma,” Clinical Cancer Research 27, no. 1 (2021): 131–140. 33208341 10.1158/1078-0432.CCR-20-2415 PMC7785656

[250] Y. Jiang , K. Zhou , Z. Sun , et al., “Non‐invasive Tumor Microenvironment Evaluation and Treatment Response Prediction in Gastric Cancer Using Deep Learning Radiomics,” Cell Reports Medicine 4, no. 8 (2023): 101146. 37557177 10.1016/j.xcrm.2023.101146 PMC10439253

[251] Q. Sun , T. Li , Z. Wei , Z. Ye , X. Zhao , and J. Jing , “Integrating Transcriptomic Data and Digital Pathology for NRG‐based Prediction of Prognosis and Therapy Response in Gastric Cancer,” Annals of Medicine 56, no. 1 (2024): 2426758. 39527470 10.1080/07853890.2024.2426758 PMC11556273

[252] Z. Lin , W. Wang , Y. Yan , Z. Ma , Z. Xiao , and K. Mao , “A Deep Learning‐based Clinical‐radiomics Model Predicting the Treatment Response of Immune Checkpoint Inhibitors (ICIs)‐based Conversion Therapy in Potentially Convertible Hepatocelluar Carcinoma Patients: A Tumor Marker Prognostic Study,” International Journal of Surgery 111, no. 5 (2025): 3342–3355. 40085751 10.1097/JS9.0000000000002322 PMC12165573

[253] K. C. Arbour , A. T. Luu , J. Luo , et al., “Deep Learning to Estimate RECIST in Patients With NSCLC Treated With PD‐1 Blockade,” Cancer Discovery 11, no. 1 (2021): 59–67. 32958579 10.1158/2159-8290.CD-20-0419 PMC7981277

[254] X. Wang , L. Yang , C. Yu , et al., “An Integrated Computational Strategy to Predict Personalized Cancer Drug Combinations by Reversing Drug Resistance Signatures,” Computers in Biology and Medicine 163 (2023): 107230. 37418899 10.1016/j.compbiomed.2023.107230

[255] S. Park , E. Silva , A. Singhal , et al., “A Deep Learning Model of Tumor Cell Architecture Elucidates Response and Resistance to CDK4/6 Inhibitors,” Nature Cancer 5, no. 7 (2024): 996–1009. 38443662 10.1038/s43018-024-00740-1 PMC11286358

[256] H. Yan , X. Ji , and B. Li , “Advancing Personalized, Predictive, and Preventive Medicine in Bladder Cancer: A Multi‐omics and Machine Learning Approach for Novel Prognostic Modeling, Immune Profiling, and Therapeutic Target Discovery,” Frontiers in Immunology 16 (2025): 1572034. 40330458 10.3389/fimmu.2025.1572034 PMC12053186

[257] S. Wang , H. Yu , Y. Gan , et al., “Mining Whole‐lung Information by Artificial Intelligence for Predicting EGFR Genotype and Targeted Therapy Response in Lung Cancer: A Multicohort Study,” Lancet Digital Health 4, no. 5 (2022): e309–e319. 35341713 10.1016/S2589-7500(22)00024-3

[258] T. Tang , Z. Zhou , M. Chen , et al., “Plasma Metabolic Profiles‐Based Prediction of Induction Chemotherapy Efficacy in Nasopharyngeal Carcinoma: Results of a Bidirectional Clinical Trial,” Clinical Cancer Research 30, no. 14 (2024): 2925–2936. 38713248 10.1158/1078-0432.CCR-23-3608 PMC11247322

[259] N. D. Maulding , J. Zou , W. Zhou , et al., “Transformer‐based Modeling of Clonal Selection and Expression Dynamics Reveals Resistance Mechanisms in Breast Cancer,” NPJ Systems Biology and Applications 11, no. 1 (2025): 5. 39794360 10.1038/s41540-024-00485-8 PMC11723929

[260] Y. Zhong , X. Chen , S. Wu , et al., “Deciphering Colorectal Cancer Radioresistance and Immune Microrenvironment: Unraveling the Role of EIF5A Through Single‐cell RNA Sequencing and Machine Learning,” Frontiers in Immunology 15 (2024): 1466226. 39290702 10.3389/fimmu.2024.1466226 PMC11405197

[261] J. Wang , J. Feng , X. Chen , et al., “Integrated Multi‐omics Analysis and Machine Learning Identify Hub Genes and Potential Mechanisms of Resistance to Immunotherapy in Gastric Cancer,” Aging (Albany NY) 16, no. 8 (2024): 7331–7356. 38656888 10.18632/aging.205760 PMC11087130

[262] C. Wu , G. Zhang , L. Wang , et al., “Spatial Proteomic Profiling Elucidates Immune Determinants of Neoadjuvant Chemo‐immunotherapy in Esophageal Squamous Cell Carcinoma,” Oncogene 43, no. 37 (2024): 2751–2767. 39122893 10.1038/s41388-024-03123-z

[263] L. Gerratana , A. A. Davis , L. Foffano , et al., “Integrating Machine Learning‐predicted Circulating Tumor Cells (CTCs) and Circulating Tumor DNA (ctDNA) in Metastatic Breast Cancer: A Proof of Principle Study on Endocrine Resistance Profiling,” Cancer Letters 609 (2025): 217325. 39577685 10.1016/j.canlet.2024.217325

[264] S. Sahni , B. Wang , D. Wu , et al., “A Machine Learning Model Reveals Expansive Downregulation of Ligand‐receptor Interactions That Enhance Lymphocyte Infiltration in Melanoma With Developed Resistance to Immune Checkpoint Blockade,” Nature Communications 15, no. 1 (2024): 8867. 10.1038/s41467-024-52555-4 PMC11473774 39402030

[265] G. Che , J. Yin , W. Wang , et al., “Circumventing Drug Resistance in Gastric Cancer: A Spatial Multi‐omics Exploration of Chemo and Immuno‐therapeutic Response Dynamics,” Drug Resistance Updates 74 (2024): 101080. 38579635 10.1016/j.drup.2024.101080

[266] Q. Zhu , H. Dai , F. Qiu , et al., “Heterogeneity of Computational Pathomic Signature Predicts Drug Resistance and Intra‐tumor Heterogeneity of Ovarian Cancer,” Translational Oncology 40 (2024): 101855. 38185058 10.1016/j.tranon.2023.101855 PMC10808968

[267] D. Zeng , Y. Yu , W. Qiu , et al., “Immunotyping the Tumor Microenvironment Reveals Molecular Heterogeneity for Personalized Immunotherapy in Cancer,” Advanced Science (Weinheim) 12, no. 25 (2025): e2417593. 10.1002/advs.202417593 PMC12224993 40433880

[268] H. Wang , Y. Yang , J. Zhang , et al., “Integrating Single‐cell RNA Sequencing and Artificial Intelligence for Multitargeted Drug Design for Combating Resistance in Liver Cancer,” NPJ Precision Oncology 9, no. 1 (2025): 309. 40897921 10.1038/s41698-025-00952-3 PMC12405525

[269] A. Singh , A. Miranda Bedate , H. J. von Richthofen , et al., “A Novel Bioinformatics Pipeline for the Identification of Immune Inhibitory Receptors as Potential Therapeutic Targets,” Elife 13 (2024): RP92870. 39377459 10.7554/eLife.92870 PMC11460946

[270] L. Zhao , H. Han , X. Zhou , et al., “Integrating Bulk and Single‐Cell Transcriptomics With Machine Learning Reveals a Heme Metabolism‐Based Panel for Lung Adenocarcinoma Chemotherapy Resistance,” International Journal of Molecular Sciences 26, no. 10 (2025): 4685. 40429829 10.3390/ijms26104685 PMC12112545

[271] N. Tavakoli , E. J. Fong , A. Coleman , et al., “Merging Metabolic Modeling and Imaging for Screening Therapeutic Targets in Colorectal Cancer,” NPJ Systems Biology and Applications 11, no. 1 (2025): 12. 39875420 10.1038/s41540-025-00494-1 PMC11775273

[272] Y. Chen , L. He , A. Ianevski , et al., “A Machine Learning‐Based Strategy Predicts Selective and Synergistic Drug Combinations for Relapsed Acute Myeloid Leukemia,” Cancer Research 85, no. 14 (2025): 2753–2768. 40354625 10.1158/0008-5472.CAN-24-3840 PMC12260508

[273] Z. Chen , Y. Chen , Y. Sun , et al., “Predicting Gastric Cancer Response to Anti‐HER2 Therapy or Anti‐HER2 Combined Immunotherapy Based on Multi‐modal Data,” Signal Transduction and Targeted Therapy 9, no. 1 (2024): 222. 39183247 10.1038/s41392-024-01932-y PMC11345439

[274] S. A. Brown , B. Y. Chung , K. Doshi , et al., “Patient Similarity and Other Artificial Intelligence Machine Learning Algorithms in Clinical Decision Aid for Shared Decision‐making in the Prevention of Cardiovascular Toxicity (PACT): A Feasibility Trial Design,” Cardiooncology 9, no. 1 (2023): 7. 36691060 10.1186/s40959-022-00151-0 PMC9869606

[275] F. Balazard , A. Bertaut , E. Bordet , et al., “Adjuvant Endocrine Therapy Uptake, Toxicity, Quality of Life, and Prediction of Early Discontinuation,” JNCI: Journal of the National Cancer Institute 115, no. 9 (2023): 1099–1108. 37434306 10.1093/jnci/djad109 PMC10483331

[276] T. Yamanaka , J. Ukita , D. Xue , et al., “Artificial Intelligence System for Predicting Hand‐foot Skin Reaction Induced by Vascular Endothelial Growth Factor Receptor Inhibitors,” Scientific Reports 15, no. 1 (2025): 9843. 40119079 10.1038/s41598-025-93471-x PMC11928579

[277] E. M. Froicu , O. M. Oniciuc , V. A. Afrasanie , et al., “The Use of Artificial Intelligence in Predicting Chemotherapy‐Induced Toxicities in Metastatic Colorectal Cancer: A Data‐Driven Approach for Personalized Oncology,” Diagnostics (Basel) 14, no. 18 (2024): 2074. 39335752 10.3390/diagnostics14182074 PMC11431340

[278] S. Y. Zhu , T. T. Yang , Y. Z. Zhao , Y. Sun , X. M. Zheng , and H. B. Xu , “Interpretable Machine Learning Model Predicting Immune Checkpoint Inhibitor‐induced Hypothyroidism: A Retrospective Cohort Study,” Cancer Science 115, no. 11 (2024): 3767–3775. 39313863 10.1111/cas.16352 PMC11531944

[279] D. S. Smith , L. Lippenszky , M. L. LeNoue‐Newton , et al., “Radiomics and Deep Learning Prediction of Immunotherapy‐Induced Pneumonitis from Computed Tomography,” JCO Clinical Cancer Informatics 9 (2025): e2400198. 39977708 10.1200/CCI-24-00198 PMC11867800

[280] Z. Zhang , Z. Wang , T. Luo , et al., “Computed Tomography and Radiation Dose Images‐based Deep‐learning Model for Predicting Radiation Pneumonitis in Lung Cancer Patients After Radiation Therapy,” Radiotherapy and Oncology 182 (2023): 109581. 36842666 10.1016/j.radonc.2023.109581

[281] Y. Zeng , Y. Hu , L. Wang , et al., “Control of Dental Calculus Prevents Severe Radiation‐Induced Oral Mucositis in Patients Undergoing Radiotherapy for Nasopharyngeal Carcinoma,” Radiotherapy and Oncology 207 (2025): 110872. 40157544 10.1016/j.radonc.2025.110872

[282] N. Lin , F. Abbas‐Aghababazadeh , J. Su , et al., “Development of Machine Learning Models for Predicting Radiation Dermatitis in Breast Cancer Patients Using Clinical Risk Factors, Patient‐Reported Outcomes, and Serum Cytokine Biomarkers,” Clinical Breast Cancer 25, no. 5 (2025): e622–e634. e6. 40155248 10.1016/j.clbc.2025.03.002

[283] X. Jiang , H. Zhao , O. L. Saldanha , et al., “An MRI Deep Learning Model Predicts Outcome in Rectal Cancer,” Radiology 307, no. 5 (2023): e222223. 37278629 10.1148/radiol.222223

[284] E. Wulczyn , D. F. Steiner , M. Moran , et al., “Interpretable Survival Prediction for Colorectal Cancer Using Deep Learning,” NPJ Digital Medicine 4, no. 1 (2021): 71. 33875798 10.1038/s41746-021-00427-2 PMC8055695

[285] D. Bychkov , N. Linder , R. Turkki , et al., “Deep Learning Based Tissue Analysis Predicts Outcome in Colorectal Cancer,” Scientific Reports 8, no. 1 (2018): 3395. 29467373 10.1038/s41598-018-21758-3 PMC5821847

[286] Z. Wu , A. E. Trevino , E. Wu , K. Swanson , H. J. Kim , and H. B. D'Angio , “Graph Deep Learning for the Characterization of Tumour Microenvironments From Spatial Protein Profiles in Tissue Specimens,” Nature Biomedical Engineering 6, no. 12 (2022): 1435–1448. 10.1038/s41551-022-00951-w 36357512

[287] S. Foersch , C. Glasner , A. C. Woerl , et al., “Multistain Deep Learning for Prediction of Prognosis and Therapy Response in Colorectal Cancer,” Nature Medicine 29, no. 2 (2023): 430–439. 10.1038/s41591-022-02134-1 36624314

[288] J. Keyl , P. Keyl , G. Montavon , et al., “Decoding Pan‐cancer Treatment Outcomes Using Multimodal Real‐world Data and Explainable Artificial Intelligence,” Nature Cancer 6, no. 2 (2025): 307–322. 39885364 10.1038/s43018-024-00891-1 PMC11864985

[289] J. Liao , Z. Xu , Y. Xie , et al., “Assessing Axillary Lymph Node Burden and Prognosis in cT1‐T2 Stage Breast Cancer Using Machine Learning Methods: A Retrospective Dual‐Institutional MRI Study,” Journal of Magnetic Resonance Imaging 61, no. 3 (2025): 1221–1231. 39175033 10.1002/jmri.29554

[290] H. Chen , Z. Wang , J. Shi , and J. Peng , “Integrating Mitochondrial and Lysosomal Gene Analysis for Breast Cancer Prognosis Using Machine Learning,” Scientific Reports 15, no. 1 (2025): 3320. 39865118 10.1038/s41598-025-86970-4 PMC11770110

[291] H. Chen , M. Chen , C. Yang , T. Tang , W. Wang , and W. Xue , “Machine Learning Based Intratumor Heterogeneity Related Signature for Prognosis and Drug Sensitivity in Breast Cancer,” Scientific Reports 15, no. 1 (2025): 10828. 40155597 10.1038/s41598-025-92695-1 PMC11953232

[292] A. H. Hounye , L. Xiong , and M. Hou , “Integrated Explainable Machine Learning and Multi‐omics Analysis for Survival Prediction in Cancer With Immunotherapy Response,” Apoptosis 30, no. 1‐2 (2025): 364–388. 39633110 10.1007/s10495-024-02050-4

[293] H. Lin , J. Hua , Z. Gong , et al., “Multimodal Radiopathological Integration for Prognosis and Prediction of Adjuvant Chemotherapy Benefit in Resectable Lung Adenocarcinoma: A Multicentre Study,” Cancer Letters 616 (2025): 217557. 39954935 10.1016/j.canlet.2025.217557

[294] T. Luo , M. Yan , M. Zhou , A. Dekker , A. L. Appelt , and Y. Ji , “Improved Prognostication of Overall Survival After Radiotherapy in Lung Cancer Patients by an Interpretable Machine Learning Model Integrating Lung and Tumor Radiomics and Clinical Parameters,” Radiologia Medica 130, no. 1 (2025): 96–109. 39542968 10.1007/s11547-024-01919-3

[295] I. Seven , D. Bayram , H. Arslan , et al., “Predicting Hepatocellular Carcinoma Survival With Artificial Intelligence,” Scientific Reports 15, no. 1 (2025): 6226. 39979406 10.1038/s41598-025-90884-6 PMC11842547

[296] Y. Xiao , S. Sun , N. Zheng , et al., “Development of PDAC Diagnosis and Prognosis Evaluation Models Based on Machine Learning,” BMC Cancer 25, no. 1 (2025): 512. 40114140 10.1186/s12885-025-13929-z PMC11924714

[297] L. Dai , K. Ye , G. Yao , et al., “Using Machine Learning for Predicting Cancer‐specific Mortality in Bladder Cancer Patients Undergoing Radical Cystectomy: A SEER‐based Study,” BMC Cancer 25, no. 1 (2025): 523. 40119324 10.1186/s12885-025-13942-2 PMC11929216

[298] Q. Deng , S. Li , Y. Zhang , Y. Jia , and Y. Yang , “Development and Validation of Interpretable Machine Learning Models to Predict Distant Metastasis and Prognosis of Muscle‐invasive Bladder Cancer Patients,” Scientific Reports 15, no. 1 (2025): 11795. 40189676 10.1038/s41598-025-96089-1 PMC11973202

[299] H. Liu , L. Ying , X. Song , X. Xiang , and S. Wei , “Development of Metastasis and Survival Prediction Model of Luminal and Non‐luminal Breast Cancer With Weakly Supervised Learning Based on Pathomics,” PeerJ 13 (2025): e18780. 39866573 10.7717/peerj.18780 PMC11759606

[300] Y. Yu , L. Cao , B. Shen , et al., “Deep Learning Radiopathomics Models Based on Contrast‐enhanced MRI and Pathologic Imaging for Predicting Vessels Encapsulating Tumor Clusters and Prognosis in Hepatocellular Carcinoma,” Radiology Imaging Cancer 7, no. 2 (2025): e240213. 40084948 10.1148/rycan.240213 PMC11966553

[301] Q. Li , W. Zhang , T. Liao , Y. Gao , Y. Zhang , and A. Jin , “An Artificial Intelligence‐Driven Preoperative Radiomic Subtype for Predicting the Prognosis and Treatment Response of Patients With Papillary Thyroid Carcinoma,” Clinical Cancer Research 31, no. 1 (2025): 139–150. 39535738 10.1158/1078-0432.CCR-24-2356

[302] F. Y. Feng , M. R. Smith , F. Saad , et al., “Digital Pathology‐Based Multimodal Artificial Intelligence Scores and Outcomes in a Randomized Phase III Trial in Men with Nonmetastatic Castration‐Resistant Prostate Cancer,” JCO Precision Oncology 9 (2025): e2400653. 39889245 10.1200/PO-24-00653

[303] J. H. Wang , M. P. Deek , A. A. Mendes , et al., “Validation of an Artificial Intelligence‐based Prognostic Biomarker in Patients With Oligometastatic Castration‐Sensitive Prostate Cancer,” Radiotherapy and Oncology 202 (2025): 110618. 39510141 10.1016/j.radonc.2024.110618 PMC11663099

[304] Y. Zhao , L. Li , S. Yuan , et al., “Artificial Intelligence‐assisted RNA‐binding Protein Signature for Prognostic Stratification and Therapeutic Guidance in Breast Cancer,” Frontiers in Immunology 16 (2025): 1583103. 40308601 10.3389/fimmu.2025.1583103 PMC12040944

[305] F. Tian , X. He , S. Wang , et al., “Integrating Single‐cell Sequencing and Machine Learning to Uncover the Role of Mitophagy in Subtyping and Prognosis of Esophageal Cancer,” Apoptosis 30, no. 3‐4 (2025): 1021–1041. 39948301 10.1007/s10495-024-02061-1

[306] J. Zhu , B. Xu , Z. Wu , Z. Yu , S. Ji , and J. Lian , “Integrative Analysis of semaphorins family Genes in Colorectal Cancer: Implications for Prognosis and Immunotherapy,” Frontiers in Immunology 16 (2025): 1536545. 40103807 10.3389/fimmu.2025.1536545 PMC11913869

[307] Y. Zhao , D. Xun , J. Chen , and X. Qi , “A Novel Machine Learning‐based Immune Prognostic Signature for Improving Clinical Outcomes and Guiding Therapy in Colorectal Cancer: An Integrated Bioinformatics and Experimental Study,” BMC Cancer 25, no. 1 (2025): 65. 39794799 10.1186/s12885-025-13437-0 PMC11724613

[308] Y. Liu , B. Bian , S. Chen , et al., “Identification and Validation of Four Serum Biomarkers with Optimal Diagnostic and Prognostic Potential for Gastric Cancer Based on Machine Learning Algorithms,” Cancer Medicine 14, no. 6 (2025): e70659. 40084401 10.1002/cam4.70659 PMC11907202

[309] X. Yan , M. Wang , L. Ji , X. Li , and B. Gao , “Machine Learning and Molecular Subtyping Reveal the Impact of Diverse Patterns of Cell Death on the Prognosis and Treatment of Hepatocellular Carcinoma,” Computational Biology and Chemistry 115 (2025): 108360. 39874853 10.1016/j.compbiolchem.2025.108360

[310] X. Liu , X. Wang , J. Ren , et al., “Machine Learning Based Identification of an Amino Acid Metabolism Related Signature for Predicting Prognosis and Immune Microenvironment in Pancreatic Cancer,” BMC Cancer 25, no. 1 (2025): 6. 39754071 10.1186/s12885-024-13374-4 PMC11697724

[311] M. Kuang , Y. Liu , H. Chen , G. Chen , T. Gao , and K. You , “Big Data Analysis and Machine Learning of the Role of Cuproptosis‐related Long Non‐coding RNAs (CuLncs) in the Prognosis and Immune Landscape of Ovarian Cancer,” Frontiers in Immunology 16 (2025): 1555782. 40070821 10.3389/fimmu.2025.1555782 PMC11893572

[312] C. Fan , Z. Huang , H. Xu , et al., “Machine Learning‐based Identification of co‐expressed Genes in Prostate Cancer and CRPC and Construction of Prognostic Models,” Scientific Reports 15, no. 1 (2025): 5679. 39956870 10.1038/s41598-025-90444-y PMC11830771

[313] J. Jiang , Y. C. Hu , N. Tyagi , et al., “Tumor‐aware, Adversarial Domain Adaptation From CT to MRI for Lung Cancer Segmentation,” Medical Image Computing and Computer‐Assisted Intervention 11071 (2018): 777–785. 30294726 10.1007/978-3-030-00934-2_86 PMC6169798

[314] T. Wang , Y. Lei , Z. Tian , et al., “Deep Learning‐based Image Quality Improvement for Low‐dose Computed Tomography Simulation in Radiation Therapy,” Journal of Medical Imaging (Bellingham, Wash) 6, no. 4 (2019): 043504. 31673567 10.1117/1.JMI.6.4.043504 PMC6811730

[315] C. Davatzikos , J. S. Barnholtz‐Sloan , S. Bakas , et al., “AI‐based Prognostic Imaging Biomarkers for Precision Neuro‐oncology: The ReSPOND Consortium,” Neuro‐Oncology 22, no. 6 (2020): 886–888. 32152622 10.1093/neuonc/noaa045 PMC7283022

[316] J. Lipkova , R. J. Chen , B. Chen , et al., “Artificial Intelligence for Multimodal Data Integration in Oncology,” Cancer Cell 40, no. 10 (2022): 1095–1110. 36220072 10.1016/j.ccell.2022.09.012 PMC10655164

[317] M. Elhaddad and S. Hamam , “AI‐Driven Clinical Decision Support Systems: An Ongoing Pursuit of Potential,” Cureus 16, no. 4 (2024): e57728. 38711724 10.7759/cureus.57728 PMC11073764

[318] B. Zhang , H. Shi , and H. Wang , “Machine Learning and AI in Cancer Prognosis, Prediction, and Treatment Selection: A Critical Approach,” Journal of Multidisciplinary Healthcare 16 (2023): 1779–1791. 37398894 10.2147/JMDH.S410301 PMC10312208

[319] G. K. Savova , I. Danciu , F. Alamudun , et al., “Use of Natural Language Processing to Extract Clinical Cancer Phenotypes From Electronic Medical Records,” Cancer Research 79, no. 21 (2019): 5463–5470. 31395609 10.1158/0008-5472.CAN-19-0579 PMC7227798

[320] V. Hernstrom , V. Josefsson , H. Sartor , et al., “Screening Performance and Characteristics of Breast Cancer Detected in the Mammography Screening With Artificial Intelligence Trial (MASAI): A Randomised, Controlled, Parallel‐group, Non‐inferiority, Single‐blinded, Screening Accuracy Study,” Lancet Digital Health 7, no. 3 (2025): e175–e183. 39904652 10.1016/S2589-7500(24)00267-X

[321] Y. Mitsuyama , H. Tatekawa , H. Takita , et al., “Comparative Analysis of GPT‐4‐based ChatGPT's Diagnostic Performance With Radiologists Using Real‐world Radiology Reports of Brain Tumors,” European Radiology 35, no. 4 (2025): 1938–1947. 39198333 10.1007/s00330-024-11032-8 PMC11913992

[322] K. Nawaz , A. Zanib , I. Shabir , et al., “Skin Cancer Detection Using Dermoscopic Images With Convolutional Neural Network,” Scientific Reports 15, no. 1 (2025): 7252. 40021731 10.1038/s41598-025-91446-6 PMC11871080

[323] V. P. G. Pushparathi , J. Shajeena , T. Kamalam , and M. Revathi , “Early Colon Cancer Prediction From Histopathological Images Using Enhanced Deep Learning With Confidence Scoring,” Cancer Investigation 43, no. 3 (2025): 205–223. 40178023 10.1080/07357907.2025.2483302

[324] S. M. Reincke , C. Espinosa , P. Chung , T. James , E. Berson , and N. Aghaeepour , “Mitigation of Outcome Conflation in Predicting Patient Outcomes Using Electronic Health Records,” Journal of the American Medical Informatics Association 32, no. 5 (2025): 920–927. 40056434 10.1093/jamia/ocaf033 PMC12012356

[325] E. J. Gong , C. S. Bang , and J. J. Lee , “Edge Artificial Intelligence Device in Real‐Time Endoscopy for Classification of Gastric Neoplasms: Development and Validation Study,” Biomimetics (Basel) 9, no. 12 (2024): 783. 39727787 10.3390/biomimetics9120783 PMC11672907

[326] M. Sevilla‐Ayensa , A. Perez‐Arruti , M. Alberich‐Inchausti , E. Larruscain‐Sarasola , J. A. Gonzalez‐Garcia , and J. A. Sistaga‐Suarez , “Validation of Step Oncology, a Platform for Head and Neck Cancer Patients Support During and After Treatment: Preliminary Results,” European Archives of Oto‐Rhino‐Laryngology 282, no. 5 (2025): 2597–2605. 39724240 10.1007/s00405-024-09170-2

[327] N. Arjmandi , M. A. Mosleh‐Shirazi , S. Mohebbi , et al., “Evaluating the Dosimetric Impact of Deep‐learning‐based Auto‐segmentation in Prostate Cancer Radiotherapy: Insights Into Real‐world Clinical Implementation and Inter‐observer Variability,” Journal of Applied Clinical Medical Physics [Electronic Resource] 26, no. 3 (2025): e14569. 39616629 10.1002/acm2.14569 PMC11905246

[328] C. Sako , C. Duan , K. Maresca , et al., “Real‐World and Clinical Trial Validation of a Deep Learning Radiomic Biomarker for PD‐(L)1 Immune Checkpoint Inhibitor Response in Advanced Non‐Small Cell Lung Cancer,” JCO Clinical Cancer Informatics 8 (2024): e2400133. 39671539 10.1200/CCI.24.00133 PMC11658027

[329] E. Wegener , M. Ng , M. Guerrieri , et al., “A Multicentre Implementation Trial of an Artificial Intelligence‐driven Biomarker to Inform Shared Decisions for Androgen Deprivation Therapy in Men Undergoing Prostate Radiotherapy: The ASTuTE Protocol,” BMC Cancer 25, no. 1 (2025): 250. 39948585 10.1186/s12885-025-13622-1 PMC11827432

[330] Y. Yu , W. Ouyang , Y. Huang , et al., “Artificial Intelligence‐based Multi‐modal Multi‐tasks Analysis Reveals Tumor Molecular Heterogeneity, Predicts Preoperative Lymph Node Metastasis and Prognosis in Papillary Thyroid Carcinoma: A Retrospective Study,” International Journal of Surgery 111, no. 1 (2025): 839–856. 38990290 10.1097/JS9.0000000000001875 PMC11745641

[331] X. Q. Zhang , Z. N. Huang , J. Wu , et al., “Machine Learning Prediction of Early Recurrence in Gastric Cancer: A Nationwide Real‐World Study,” Annals of Surgical Oncology 32, no. 4 (2025): 2637–2650. 39738899 10.1245/s10434-024-16701-y

[332] C. Schukow , S. C. Smith , E. Landgrebe , et al., “Application of ChatGPT in Routine Diagnostic Pathology: Promises, Pitfalls, and Potential Future Directions,” Advances in Anatomic Pathology 31, no. 1 (2024): 15–21. 37501529 10.1097/PAP.0000000000000406

[333] A. Mohammed and R. Kora , “A Comprehensive Review on Ensemble Deep Learning: Opportunities and Challenges,” Journal of King Saud University—Computer and Information Sciences 35, no. 2 (2023): 757–774.

[334] R. P. Singh , G. L. Hom , M. D. Abramoff , J. P. Campbell , and M. F. Chiang , “Intelligence AAOTFoA. Current Challenges and Barriers to Real‐World Artificial Intelligence Adoption for the Healthcare System, Provider, and the Patient,” Translational Vision Science & Technology 9, no. 2 (2020): 45. 10.1167/tvst.9.2.45 PMC7443115 32879755

[335] H. Hua , Y. Deng , J. Zhang , X. Zhou , T. Zhang , and B. L. Khoo , “AIEgen‐deep: Deep Learning of Single AIEgen‐imaging Pattern for Cancer Cell Discrimination and Preclinical Diagnosis,” Biosensors & Bioelectronics 253 (2024): 116086. 38422811 10.1016/j.bios.2024.116086

[336] M. Calvarese , E. Corbetta , J. Contreras , et al., “Endomicroscopic AI‐driven Morphochemical Imaging and Fs‐laser Ablation for Selective Tumor Identification and Selective Tissue Removal,” Science Advances 10, no. 50 (2024): eado9721. 39661684 10.1126/sciadv.ado9721 PMC11633757

[337] J. Rappaport , Q. Chen , T. McGuire , A. Daugherty‐Lopes , and R. Goldszmid , “Machine Learning Approach to Assess Brain Metastatic Burden in Preclinical Models,” Methods in Cell Biology 190 (2024): 25–49. 39515881 10.1016/bs.mcb.2024.10.001

[338] J. Rodrigues , A. Amin , S. Chandra , et al., “Machine Learning Enabled Photoacoustic Spectroscopy for Noninvasive Assessment of Breast Tumor Progression in Vivo: A Preclinical Study,” ACS Sensors 9, no. 2 (2024): 589–601. 38288735 10.1021/acssensors.3c01085 PMC10897932

[339] N. Fraunhoffer , P. Hammel , T. Conroy , et al., “Development and Validation of AI‐assisted Transcriptomic Signatures to Personalize Adjuvant Chemotherapy in Patients With Pancreatic Ductal Adenocarcinoma,” Annals of Oncology 35, no. 9 (2024): 780–791. 38906254 10.1016/j.annonc.2024.06.010

[340] S. Benzekry , M. Mastri , C. Nicolo , and J. M. L. Ebos , “Machine‐learning and Mechanistic Modeling of Metastatic Breast Cancer After Neoadjuvant Treatment,” Plos Computational Biology 20, no. 5 (2024): e1012088. 38701089 10.1371/journal.pcbi.1012088 PMC11095706

[341] N. R. Thiruvengadam , P. Solaimani , M. Shrestha , S. Buller , R. Carson , and B. Reyes‐Garcia , “The Efficacy of Real‐time Computer‐aided Detection of Colonic Neoplasia in Community Practice: A Pragmatic Randomized Controlled Trial,” Clinical Gastroenterology and Hepatology 22, no. 11 (2024): 2221–2230. e15. 38437999 10.1016/j.cgh.2024.02.021

[342] R. Djinbachian , M. Taghiakbari , A. Barkun , et al., “Optimized Computer‐assisted Technique for Increasing Adenoma Detection During Colonoscopy: A Randomized Controlled Trial,” Surgical Endoscopy 39, no. 2 (2025): 1120–1127. 39702564 10.1007/s00464-024-11466-7

[343] T. K. Lui , C. P. Lam , E. W. To , et al., “Endocuff with or without Artificial Intelligence‐Assisted Colonoscopy in Detection of Colorectal Adenoma: A Randomized Colonoscopy Trial,” American Journal of Gastroenterology 119, no. 7 (2024): 1318–1325. 38305278 10.14309/ajg.0000000000002684 PMC11208055

[344] M. Desai , K. Ausk , D. Brannan , R. Chhabra , W. Chan , and M. Chiorean , “Use of a Novel Artificial Intelligence System Leads to the Detection of Significantly Higher Number of Adenomas during Screening and Surveillance Colonoscopy: Results from a Large, Prospective, US Multicenter, Randomized Clinical Trial,” American Journal of Gastroenterology 119, no. 7 (2024): 1383–1391. 38235741 10.14309/ajg.0000000000002664

[345] L. Yao , X. Li , Z. Wu , et al., “Effect of Artificial Intelligence on Novice‐performed Colonoscopy: A Multicenter Randomized Controlled Tandem Study,” Gastrointestinal Endoscopy 99, no. 1 (2024): 91–99. e9. 37536635 10.1016/j.gie.2023.07.044

[346] K. Miyaguchi , Y. Tsuzuki , N. Hirooka , et al., “Linked‐color Imaging With or Without Artificial Intelligence for Adenoma Detection: A Randomized Trial,” Endoscopy 56, no. 5 (2024): 376–383. 38191000 10.1055/a-2239-8145 PMC11038826

[347] X. L. Yuan , W. Liu , Y. X. Lin , et al., “Effect of an Artificial Intelligence‐assisted System on Endoscopic Diagnosis of Superficial Oesophageal Squamous Cell Carcinoma and Precancerous Lesions: A Multicentre, Tandem, Double‐blind, Randomised Controlled Trial,” Lancet Gastroenterology and Hepatology 9, no. 1 (2024): 34–44. 37952555 10.1016/S2468-1253(23)00276-5

[348] S. W. Li , L. H. Zhang , Y. Cai , et al., “Deep Learning Assists Detection of Esophageal Cancer and Precursor Lesions in a Prospective, Randomized Controlled Study,” Science Translational Medicine 16, no. 743 (2024): eadk5395. 38630847 10.1126/scitranslmed.adk5395

[349] W. T. Tang , C. Q. Su , J. Lin , Z. W. Xia , S. S. Lu , and X. N. Hong , “T2‐FLAIR Mismatch Sign and Machine Learning‐based Multiparametric MRI Radiomics in Predicting IDH Mutant 1p/19q Non‐co‐deleted Diffuse Lower‐grade Gliomas,” Clinical Radiology 79, no. 5 (2024): e750–e758. 38360515 10.1016/j.crad.2024.01.021

[350] M. Salim , Y. Liu , M. Sorkhei , et al., “AI‐based Selection of Individuals for Supplemental MRI in Population‐based Breast Cancer Screening: The Randomized ScreenTrustMRI Trial,” Nature Medicine 30, no. 9 (2024): 2623–2630. 10.1038/s41591-024-03093-5 PMC11405258 38977914

[351] A. Saha , J. S. Bosma , J. J. Twilt , et al., “Artificial Intelligence and Radiologists in Prostate Cancer Detection on MRI (PI‐CAI): An International, Paired, Non‐inferiority, Confirmatory Study,” The Lancet Oncology 25, no. 7 (2024): 879–887. 38876123 10.1016/S1470-2045(24)00220-1 PMC11587881

[352] R. Deng , Y. Liu , K. Wang , et al., “Comparison of MRI Artificial Intelligence‐guided Cognitive Fusion‐targeted Biopsy versus Routine Cognitive Fusion‐targeted Prostate Biopsy in Prostate Cancer Diagnosis: A Randomized Controlled Trial,” BMC Medicine [Electronic Resource] 22, no. 1 (2024): 530. 39533250 10.1186/s12916-024-03742-z PMC11559106

[353] P. Papachristou , M. Soderholm , J. Pallon , et al., “Evaluation of an Artificial Intelligence‐based Decision Support for the Detection of Cutaneous Melanoma in Primary Care: A Prospective Real‐life Clinical Trial,” British Journal of Dermatology 191, no. 1 (2024): 125–133. 38234043 10.1093/bjd/ljae021

[354] O. Ortiz , M. Daca‐Alvarez , L. Rivero‐Sanchez , A. Z. Gimeno‐Garcia , M. Carrillo‐Palau , and V. Alvarez , “An Artificial Intelligence‐assisted System versus White Light Endoscopy Alone for Adenoma Detection in Individuals With Lynch Syndrome (TIMELY): An International, Multicentre, Randomised Controlled Trial,” Lancet Gastroenterology and Hepatology 9, no. 9 (2024): 802–810. 39033774 10.1016/S2468-1253(24)00187-0

[355] E. Nakao , T. Yoshio , Y. Kato , et al., “Randomized Controlled Trial of an Artificial Intelligence Diagnostic System for the Detection of Esophageal Squamous Cell Carcinoma in Clinical Practice,” Endoscopy 57, no. 3 (2025): 210–217. 39317205 10.1055/a-2421-3194

[356] J. Scholer , M. Alavanja , T. de Lange , S. Yamamoto , P. Hedenstrom , and J. Varkey , “Impact of AI‐aided Colonoscopy in Clinical Practice: A Prospective Randomised Controlled Trial,” BMJ Open Gastroenterology 11, no. 1 (2024): e001247. 10.1136/bmjgast-2023-001247 PMC10870789 38290758

[357] H. Zheng , L. Jian , L. Li , W. Liu , and W. Chen , “Prior Clinico‐Radiological Features Informed Multi‐Modal MR Images Convolution Neural Network: A Novel Deep Learning Framework for Prediction of Lymphovascular Invasion in Breast Cancer,” Cancer Medicine 13, no. 3 (2024): e6932. 38230837 10.1002/cam4.6932 PMC10905682

[358] C. van Dooijeweert , R. N. Flach , N. D. Ter Hoeve , C. P. H. Vreuls , R. Goldschmeding , and J. E. Freund , “Clinical Implementation of Artificial‐intelligence‐assisted Detection of Breast Cancer Metastases in Sentinel Lymph Nodes: The CONFIDENT‐B Single‐center, Non‐randomized Clinical Trial,” Nature Cancer 5, no. 8 (2024): 1195–1205. 38937624 10.1038/s43018-024-00788-z PMC11358151

[359] X. Luo , Y. Yang , S. Yin , et al., “Automated Segmentation of Brain Metastases With Deep Learning: A Multi‐center, Randomized Crossover, Multi‐reader Evaluation Study,” Neuro‐Oncology 26, no. 11 (2024): 2140–2151. 38991556 10.1093/neuonc/noae113 PMC11639187

[360] H. Wang , Y. Chen , J. Qiu , et al., “Machine Learning Based on SPECT/CT to Differentiate Bone Metastasis and Benign Bone Lesions in Lung Malignancy Patients,” Medical Physics 51, no. 4 (2024): 2578–2588. 37966123 10.1002/mp.16839

[361] Y. F. Zhang , C. Zhou , S. Guo , et al., “Deep Learning Algorithm‐based Multimodal MRI Radiomics and Pathomics Data Improve Prediction of Bone Metastases in Primary Prostate Cancer,” Journal of Cancer Research and Clinical Oncology 150, no. 2 (2024): 78. 38316655 10.1007/s00432-023-05574-5 PMC10844393

[362] M. B. Zhang , Z. L. Meng , Y. Mao , et al., “Cervical Lymph Node Metastasis Prediction From Papillary Thyroid Carcinoma US Videos: A Prospective Multicenter Study,” BMC Medicine [Electronic Resource] 22, no. 1 (2024): 153. 38609953 10.1186/s12916-024-03367-2 PMC11015607

[363] Y. Du , Y. Xiao , W. Guo , et al., “Development and Validation of an Ultrasound‐based Deep Learning Radiomics Nomogram for Predicting the Malignant Risk of Ovarian Tumours,” Biomedical Engineering Online [Electronic Resource] 23, no. 1 (2024): 41. 38594729 10.1186/s12938-024-01234-y PMC11003110

[364] K. R. Quan , W. R. Lin , J. B. Hong , et al., “A Machine Learning Approach for Predicting Radiation‐induced Hypothyroidism in Patients With Nasopharyngeal Carcinoma Undergoing Tomotherapy,” Scientific Reports 14, no. 1 (2024): 8436. 38600141 10.1038/s41598-024-59249-3 PMC11006930

[365] L. Xu , H. Si , F. Zhuang , et al., “Predicting Therapeutic Response to Neoadjuvant Immunotherapy Based on an Integration Model in Resectable Stage IIIA (N2) Non‐small Cell Lung Cancer,” Journal of Thoracic and Cardiovascular Surgery 169, no. 1 (2025): 242–253. e4. 38763304 10.1016/j.jtcvs.2024.05.006

[366] F. Schon , A. Kieslich , H. Nebelung , et al., “Comparative Analysis of Radiomics and Deep‐learning Algorithms for Survival Prediction in Hepatocellular Carcinoma,” Scientific Reports 14, no. 1 (2024): 590. 38182664 10.1038/s41598-023-50451-3 PMC10770355

[367] K. Atsou , A. Auperin , J. Guigay , S. Salas , and S. Benzekry , “Mechanistic Learning for Predicting Survival Outcomes in Head and Neck Squamous Cell Carcinoma,” CPT Pharmacometrics and System Pharmacology 14, no. 3 (2025): 540–550. 10.1002/psp4.13294 PMC11919269 39722558

[368] C. M. Benedum , A. Sondhi , E. Fidyk , et al., “Replication of Real‐World Evidence in Oncology Using Electronic Health Record Data Extracted by Machine Learning,” Cancers (Basel) 15, no. 6 (2023): 1853. 36980739 10.3390/cancers15061853 PMC10046618

[369] P. Abrisqueta‐Costa , J. A. Garcia‐Marco , A. Gutierrez , et al., “Real‐World Evidence on Adverse Events and Healthcare Resource Utilization in Patients With Chronic Lymphocytic Leukaemia in Spain Using Natural Language Processing: The SRealCLL Study,” Cancers (Basel) 16, no. 23 (2024): 4004. 39682190 10.3390/cancers16234004 PMC11639754

[370] J. Loscertales , P. Abrisqueta‐Costa , A. Gutierrez , et al., “Real‐World Evidence on the Clinical Characteristics and Management of Patients With Chronic Lymphocytic Leukemia in Spain Using Natural Language Processing: The SRealCLL Study,” Cancers (Basel) 15, no. 16 (2023): 4047. 37627075 10.3390/cancers15164047 PMC10452602

[371] D. S. Bitterman , E. Goldner , S. Finan , et al., “An End‐to‐End Natural Language Processing System for Automatically Extracting Radiation Therapy Events from Clinical Texts,” International Journal of Radiation and Oncology in Biology and Physics 117, no. 1 (2023): 262–273. 10.1016/j.ijrobp.2023.03.055 PMC10522797 36990288

[372] G. Varma , R. K. Yenukoti , M. P. Kumar , et al., “A Deep Learning‐Enabled Workflow to Estimate Real‐World Progression‐Free Survival in Patients with Metastatic Breast Cancer: Study Using Deidentified Electronic Health Records,” JMIR Cancer 11 (2025): e64697. 40372953 10.2196/64697 PMC12097284

[373] R. Moulson , J. Law , A. Sacher , et al., “Real‐World Outcomes of Patients With Advanced Epidermal Growth Factor Receptor‐Mutated Non‐Small Cell Lung Cancer in Canada Using Data Extracted by Large Language Model‐Based Artificial Intelligence,” Current Oncology 31, no. 4 (2024): 1947–1960. 38668049 10.3390/curroncol31040146 PMC11049467

[374] S. A. van Laar , K. B. Gombert‐Handoko , H. J. Guchelaar , and J. Zwaveling , “An Electronic Health Record Text Mining Tool to Collect Real‐World Drug Treatment Outcomes: A Validation Study in Patients with Metastatic Renal Cell Carcinoma,” Clinical Pharmacology & Therapeutics 108, no. 3 (2020): 644–652. 32575147 10.1002/cpt.1966 PMC7484987

[375] Y. H. Karimi , D. W. Blayney , A. W. Kurian , et al., “Development and Use of Natural Language Processing for Identification of Distant Cancer Recurrence and Sites of Distant Recurrence Using Unstructured Electronic Health Record Data,” JCO Clinical Cancer Informatics 5 (2021): 469–478. 33929889 10.1200/CCI.20.00165 PMC8462655

[376] X. Wang , H. Lee , B. Haaland , et al., “A Matching‐based Machine Learning Approach to Estimating Optimal Dynamic Treatment Regimes With Time‐to‐event Outcomes,” Statistical Methods in Medical Research 33, no. 5 (2024): 794–806. 38502008 10.1177/09622802241236954

[377] T. Yamashita , Y. Wakata , H. Nakaguma , et al., “Machine Learning for Classification of Postoperative Patient Status Using Standardized Medical Data,” Computer Methods and Programs in Biomedicine 214 (2022): 106583. 34959156 10.1016/j.cmpb.2021.106583

[378] L. Hu , J. Ji , H. Liu , and R. Ennis , “A Flexible Approach for Assessing Heterogeneity of Causal Treatment Effects on Patient Survival Using Large Datasets With Clustered Observations,” International Journal of Environmental Research and Public Health 19, no. 22 (2022): 14903. 36429621 10.3390/ijerph192214903 PMC9690785

[379] O. Tredan , M. Laurent , M. Gilberg , et al., “Innovative Approach for a Typology of Treatment Sequences in Early Stage HER2 Positive Breast Cancer Patients Treated with Trastuzumab in the French National Hospital Database,” Cancer Informatics 21 (2022): 11769351221135134. 36386278 10.1177/11769351221135134 PMC9661546

[380] Y. P. V. Mbous , Z. A. Siddiqui , M. Bharmal , et al., “Type of Pre‐existing Chronic Conditions and Their Associations With Merkel Cell Carcinoma (MCC) Treatment: Prediction and Interpretation Using Machine Learning Methods,” PLoS ONE 20, no. 7 (2025): e0327964. 40680081 10.1371/journal.pone.0327964 PMC12274004

[381] R. Knevel and K. P. Liao , “From Real‐world Electronic Health Record Data to Real‐world Results Using Artificial Intelligence,” Annals of the Rheumatic Diseases 82, no. 3 (2023): 306–311. 36150748 10.1136/ard-2022-222626 PMC9933153

[382] M. M. Voets , J. Veltman , C. H. Slump , S. Siesling , and H. Koffijberg , “Systematic Review of Health Economic Evaluations Focused on Artificial Intelligence in Healthcare: The Tortoise and the Cheetah,” Value in Health: The Journal of the International Society for Pharmacoeconomics and Outcomes Research 25, no. 3 (2022): 340–349. 35227444 10.1016/j.jval.2021.11.1362

[383] P. S. Aravazhi , P. Gunasekaran , N. Z. Y. Benjamin , et al., “The Integration of Artificial Intelligence Into Clinical Medicine: Trends, Challenges, and Future Directions,” Disease‐A‐Month 71, no. 6 (2025): 101882. 40140300 10.1016/j.disamonth.2025.101882

[384] A. Aggarwal , S. Bharadwaj , G. Corredor , T. Pathak , S. Badve , and A. Madabhushi , “Artificial Intelligence in Digital Pathology—time for a Reality Check,” Nature Reviews Clinical Oncology 22, no. 4 (2025): 283–291. 10.1038/s41571-025-00991-6 39934323

[385] G. Duwe , D. Mercier , C. Wiesmann , et al., “Challenges and Perspectives in Use of Artificial Intelligence to Support Treatment Recommendations in Clinical Oncology,” Cancer Medicine 13, no. 12 (2024): e7398. 38923826 10.1002/cam4.7398 PMC11196383

[386] K. Akimoto , K. Taparra , T. Brown , and M. I. Patel , “Diversity in Cancer Care: Current Challenges and Potential Solutions to Achieving Equity in Clinical Trial Participation,” Cancer Journal (Sudbury, Massachusetts) 29, no. 6 (2023): 310–315. 10.1097/PPO.0000000000000675 37963364

[387] M. G. Hanna , L. Pantanowitz , B. Jackson , et al., “Ethical and Bias Considerations in Artificial Intelligence/Machine Learning,” Modern Pathology 38, no. 3 (2025): 100686. 39694331 10.1016/j.modpat.2024.100686

[388] L. H. Nazer , R. Zatarah , S. Waldrip , et al., “Bias in Artificial Intelligence Algorithms and Recommendations for Mitigation,” PLOS Digital Health 2, no. 6 (2023): e0000278. 37347721 10.1371/journal.pdig.0000278 PMC10287014

[389] C. Elendu , D. C. Amaechi , T. C. Elendu , et al., “Ethical Implications of AI and Robotics in Healthcare: A Review,” Medicine 102, no. 50 (2023): e36671. 38115340 10.1097/MD.0000000000036671 PMC10727550

[390] W. Lotter , M. J. Hassett , N. Schultz , K. L. Kehl , E. M. Van Allen , and E. Cerami , “Artificial Intelligence in Oncology: Current Landscape, Challenges, and Future Directions,” Cancer Discovery 14, no. 5 (2024): 711–726. 38597966 10.1158/2159-8290.CD-23-1199 PMC11131133

[391] S. I. Lambert , M. Madi , S. Sopka , et al., “An Integrative Review on the Acceptance of Artificial Intelligence Among Healthcare Professionals in Hospitals,” NPJ Digital Medicine 6, no. 1 (2023): 111. 37301946 10.1038/s41746-023-00852-5 PMC10257646

[392] A. Kiseleva , D. Kotzinos , and P. De Hert , “Transparency of AI in Healthcare as a Multilayered System of Accountabilities: Between Legal Requirements and Technical Limitations,” Frontiers in Artificial Intelligence 5 (2022): 879603. 35707765 10.3389/frai.2022.879603 PMC9189302

[393] E. Marcus and J. Teuwen , “Artificial Intelligence and Explanation: How, Why, and When to Explain Black Boxes,” European Journal of Radiology 173 (2024): 111393. 38417186 10.1016/j.ejrad.2024.111393

[394] D. Muhammad and M. Bendechache , “Unveiling the Black Box: A Systematic Review of Explainable Artificial Intelligence in Medical Image Analysis,” Computational and Structural Biotechnology Journal 24 (2024): 542–560. 39252818 10.1016/j.csbj.2024.08.005 PMC11382209

[395] R. Lavelle‐Hill , G. Smith , H. Deininger , and K. Murayama , “An Explainable Artificial Intelligence Handbook for Psychologists: Methods, Opportunities, and Challenges,” Psychological Methods (2025). 10.1037/met0000772 40742683

[396] V. Hassija , V. Chamola , A. Mahapatra , et al., “Interpreting Black‐Box Models: A Review on Explainable Artificial Intelligence,” Cognitive Computation 16, no. 1 (2024): 45–74.

[397] M. Harishbhai Tilala , P. Kumar Chenchala , A. Choppadandi , et al., “Ethical Considerations in the Use of Artificial Intelligence and Machine Learning in Health Care: A Comprehensive Review,” Cureus 16, no. 6 (2024): e62443. 39011215 10.7759/cureus.62443 PMC11249277

[398] A. Arora , J. E. Alderman , J. Palmer , et al., “The Value of Standards for Health Datasets in Artificial Intelligence‐based Applications,” Nature Medicine 29, no. 11 (2023): 2929–2938. 10.1038/s41591-023-02608-w PMC10667100 37884627

[399] N. Norori , Q. Hu , F. M. Aellen , F. D. Faraci , and A. Tzovara , “Addressing Bias in Big Data and AI for Health Care: A Call for Open Science,” Patterns (N Y) 2, no. 10 (2021): 100347. 34693373 10.1016/j.patter.2021.100347 PMC8515002

[400] I. W. Price , S. Gerke , and I. G. Cohen . Liability for Use of Artificial Intelligence in medicine. In: B. Solaiman , and I. G. Cohen , editors. Research Handbook on Health, AI and the Law″ (Cheltenham, UK 2024): 150–166.

[401] C. Cestonaro , A. Delicati , B. Marcante , L. Caenazzo , and P. Tozzo , “Defining Medical Liability When Artificial Intelligence Is Applied on Diagnostic Algorithms: A Systematic Review,” Frontiers in Medicine (Lausanne) 10 (2023): 1305756. 10.3389/fmed.2023.1305756 PMC10711067 38089864

[402] F. Boge and A. Mosig , “Causality and Scientific Explanation of Artificial Intelligence Systems in Biomedicine,” Pflugers Archiv: European Journal of Physiology 477, no. 4 (2025): 543–554. 39470762 10.1007/s00424-024-03033-9 PMC11958387

[403] Y. Abas Mohamed , B. Ee Khoo , M. Shahrimie Mohd Asaari , M. Ezane Aziz , and F. Rahiman Ghazali , “Decoding the Black Box: Explainable AI (XAI) for Cancer Diagnosis, Prognosis, and Treatment Planning‐A state‐of‐the Art Systematic Review,” International Journal of Medical Informatics 193 (2025): 105689. 39522406 10.1016/j.ijmedinf.2024.105689

[404] B. Mueller , T. Kinoshita , A. Peebles , M. A. Graber , and S. Lee , “Artificial Intelligence and Machine Learning in Emergency Medicine: A Narrative Review,” Acute Medicine & Surgery 9, no. 1 (2022): e740. 35251669 10.1002/ams2.740 PMC8887797

[405] H. Patel , A. Chopra , and D. S. Rajput , “Myint Aung D. Interactive XAI for Personalized and Trusted Healthcare: Need of the Hour,” International Journal of Surgery 110, no. 9 (2024): 5869–5870. 38781038 10.1097/JS9.0000000000001643 PMC11392082

[406] B. A. van Dort , J. Lopez Canizares , R. Marcilly , L. Peute , and D. Sent , “Enhancing Clinical Decision Support: A Heuristic Evaluation of Explainable AI in Healthcare Dashboards,” Studies in Health Technology and Informatics 327 (2025): 343–347. 40380452 10.3233/SHTI250341

[407] N. Mitra , J. Roy , and D. Small , “The Future of Causal Inference,” American Journal of Epidemiology 191, no. 10 (2022): 1671–1676. 35762132 10.1093/aje/kwac108 PMC9991894

[408] P. Linardatos , V. Papastefanopoulos , S. Kotsiantis , and A. I. Explainable , “A Review of Machine Learning Interpretability Methods,” Entropy (Basel) 23, no. 1 (2020): 18. 33375658 10.3390/e23010018 PMC7824368

[409] X. Yang , J. Jia , X. Zhou , and S. Wang , “The Future of Artificial Intelligence: Time to Embrace More International Collaboration,” Innovation (Cambridge) 5, no. 6 (2024): 100703. 10.1016/j.xinn.2024.100703 PMC11472620 39403172

[410] Q. Liu , C. Huang , G. Zhan , Y. Guan , and S. Li , “The Need for Expansion of Global Collaborations on AI in Oncology,” Lancet 405, no. 10487 (2025): 1339. 10.1016/S0140-6736(25)00634-8 40253093

[411] M. Tajabadi , L. Grabenhenrich , A. Ribeiro , M. Leyer , and D. Heider , “Sharing Data with Shared Benefits: Artificial Intelligence Perspective,” Journal of Medical Internet Research [Electronic Resource] 25 (2023): e47540. 37642995 10.2196/47540 PMC10498316

[412] C. Mennella , U. Maniscalco , G. De Pietro , and M. Esposito , “Ethical and Regulatory Challenges of AI Technologies in Healthcare: A Narrative Review,” Heliyon 10, no. 4 (2024): e26297. 38384518 10.1016/j.heliyon.2024.e26297 PMC10879008

[413] N. Schwalbe and B. Wahl , “Artificial Intelligence and the Future of Global Health,” Lancet 395, no. 10236 (2020): 1579–1586. 32416782 10.1016/S0140-6736(20)30226-9 PMC7255280

[414] F. Olan , E. Ogiemwonyi Arakpogun , J. Suklan , F. Nakpodia , N. Damij , and U. Jayawickrama , “Artificial Intelligence and Knowledge Sharing: Contributing Factors to Organizational Performance,” Journal of Business Research 145 (2022): 605–615.

[415] V. S. Viswanathan , V. Parmar , and A. Madabhushi , “Towards Equitable AI in Oncology,” Nature Reviews Clinical Oncology 21, no. 8 (2024): 628–637. 10.1038/s41571-024-00909-8 38849530

[416] D. Ueda , T. Kakinuma , S. Fujita , et al., “Fairness of Artificial Intelligence in Healthcare: Review and Recommendations,” Japanese Journal of Radiology 42, no. 1 (2024): 3–15. 37540463 10.1007/s11604-023-01474-3 PMC10764412

[417] A. Singhal , N. Neveditsin , H. Tanveer , and V. Mago , “Toward Fairness, Accountability, Transparency, and Ethics in AI for Social Media and Health Care: Scoping Review,” JMIR Medical Informatics Medical Informatics 12 (2024): e50048. 10.2196/50048 PMC11024755 38568737

[418] M. M. Mir , G. M. Mir , N. T. Raina , et al., “Application of Artificial Intelligence in Medical Education: Current Scenario and Future Perspectives,” Journal of Advances in Medical Education & Professionalism 11, no. 3 (2023): 133–140. 37469385 10.30476/JAMP.2023.98655.1803 PMC10352669

[419] S. Garcia‐Saiso , M. Marti , K. Pesce , et al., “Artificial Intelligence as a Potential Catalyst to a More Equitable Cancer Care,” JMIR Cancer 10 (2024): e57276. 39133537 10.2196/57276 PMC11347894

[420] A. A. Funa and R. A. E. Gabay , “Policy Guidelines and Recommendations on AI Use in Teaching and Learning: A Meta‐synthesis Study,” Social Sciences & Humanities Open 11 (2025): 101221.

